# Robust estimates of biodiversity change require high-resolution time series

**DOI:** 10.1038/s41467-026-75321-0

**Published:** 2026-07-07

**Authors:** Daniela Cortés-Guzmán, James S. Sinclair, Jukka Aroviita, Iker Azpiroz, Milo L. de Baat, Ignacio Bañares, Elmar Becker, Miguel Cañedo-Argüelles, Eddy Cosson, David Cunillera-Montcusí, Rémi Escaffre, Martial Ferréol, Marie Anne Eurie Forio, Peter Goethals, Alexia M. González-Ferreras, Kaisa-Leena Huttunen, Aitor Larrañaga, Eva S. López, Manu Rubio, Rudy Vannevel, Martin Wilkes, Peter Haase

**Affiliations:** 1 Senckenberg – Leibniz Institution for Biodiversity and Earth System Research, Senckenberg Research Institute and Natural History Museum Frankfurt, Gelnhausen, Germany; 2 Marine and Freshwater Solutions, Finnish Environment Institute, Oulu, Finland; 3 Ekolur, C° de Astigarraga 2, 4D, Oiartzun, Spain; 4 Institute for Biodiversity and Ecosystem Dynamics, University of Amsterdam, Amsterdam, the Netherlands; 5 Department of Sustainability, Gipuzkoa Provincial Council, Donostia-San Sebastián, Spain; 6 SHE2 group (Surface Hydrology, Ecology and Erosion), FEHM-Lab, Institute of Environmental Assessment and Water Research, IDAEA, CSIC, Barcelona, Catalonia Spain; 7 Department for Water and Aquatic Ecosystems, French Biodiversity Agency, Vincennes, France; 8 Institute of Aquatic Ecology, HUN-REN Centre for Ecological Research, Budapest, Hungary; 9 Data & Methodology Support Unit, French Biodiversity Agency, Vincennes, France; 10 INRAE, UR RiverLy, Centre de Lyon-Villeurbanne, Villeurbanne, France; 11 Department of Animal Sciences and Aquatic Ecology, Ghent University, Gent, Belgium; 12 IHCantabria—Instituto de Hidráulica Ambiental de la Universidad de Cantabria, Santander, Spain; 13 Nature Solutions, Finnish Environment Institute, Oulu, Finland; 14 Ecology and Genetics Research Unit, University of Oulu, Oulu, Finland; 15 Department of Plant Biology and Ecology, University of the Basque Country (UPV/EHU), Bilbao, Spain; 16 Anbiotek, Erandio, Spain; 17 Flanders Environment Agency, Aalst, Belgium; 18 School of Life Sciences, University of Essex, Colchester, UK; 19 Faculty of Biology, University of Duisburg-Essen, Essen, Germany

**Keywords:** Biodiversity, Community ecology, Freshwater ecology

## Abstract

Robust estimates of biodiversity change are essential to inform management and conservation, and to address global biodiversity loss. However, common temporal data limitations may compromise trend accuracy. Here, we test how temporal resolution influences biodiversity trends using 1,353 river invertebrate time series collected annually for ≥ 10 years across 18 European countries. We simulate reduced sampling frequencies and durations and compare these trends to the complete time series. Reducing frequency from annual to every 2–6 years resulted in 87–73% of sites matching in trend directions, but only 78–39% matching in magnitude. Reducing duration from 10 to 9–2 years resulted in 88–52% direction matches and 86–8% magnitude matches. Similar results were observed for longer time series ( ≥ 20 years). Additionally, a comparison of two real-world monitoring datasets with different temporal resolutions shows that 53% of sites matched in direction, but only 12% in magnitude. Our findings indicate that biodiversity change magnitude is more sensitive to temporal resolution than direction. Consequently, accurate estimates of both direction and magnitude require high-resolution time series, whereas lower-resolution data may only reliably capture direction. These results highlight the value and limitations of temporal biodiversity data, and help plan future monitoring.

## Abstract (web summary)

Temporal data limitations can bias estimates of biodiversity change needed to address biodiversity loss. This study shows that reliable estimates of trend direction and magnitude require high resolution time series, whereas low-resolution data often only capture direction.

## Introduction

Warnings of biodiversity loss^1,2^ and the threatened status of many species^3–5^ have spurred global efforts to study biodiversity change. These efforts are exemplified by expanded global monitoring (e.g., as part of the Kunming-Montreal Global Biodiversity Framework, KM GBF; see CBD/COP/15/5) and by the growing number of broad-scale (i.e., global or continental) studies of biodiversity change in terrestrial, marine, and freshwater ecosystems. However, such studies commonly report finding an approximately equal mixture of neutral, increasing, and decreasing site-level biodiversity trends^6–15^. This discrepancy between concerns of global losses versus studies finding no overall trends raises a key question: are the results of these studies reliable, or are limitations in biodiversity datasets compromising our ability to quantify trends accurately^16–22?^

**Fig. 1:**
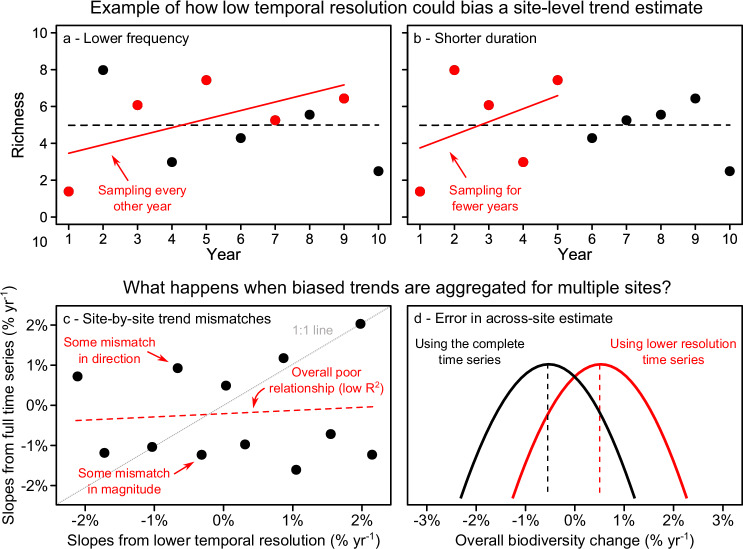
Theoretical examples of how temporal data limitations may affect biodiversity trends. Reducing the **a** sampling frequency or **b** sampling duration of a time series can cause estimated trends (red lines) to deviate markedly from those quantified using a higher temporal resolution (black, dashed lines). These biases could lead to **c** an accumulation of numerous trend direction and magnitude errors when trends are aggregated among multiple sites, driving **d** differences in across-site estimates of biodiversity change (e.g., the average and distribution of trend values).

Biodiversity data often suffer from a variety of limitations that could lead to disagreement and unreliability in trend estimates, including spatial biases and heterogeneity in data characteristics^16,23^. A common limitation is low temporal resolution, often arising from opportunistic data collection or constraints on monitoring resources, including funding, time, and personnel. Consequently, many broad-scale biodiversity studies include time series with a low sampling frequency or duration, even sometimes just two or three sampling years per site^7,8,10,14^. Such sparse time series could bias biodiversity trends in two principal ways. First, temporal trends quantified from a low number of samples have low statistical power, defined in this context as a low probability of detecting the correct trend value^24,25^. Second, because communities exhibit natural interannual variability, using low-resolution time series can produce trends that reflect stochastic fluctuations rather than real change^19,26^ (Fig. 1a, b). These two sources of trend bias have important implications because they may accumulate when trends are aggregated across multiple sites (Fig. 1c, d), inflating error rates and leading to incorrect conclusions about biodiversity change that waste limited conservation resources and misinform management recommendations^20,27,28^. Various strategies have been developed to counteract these challenges, including statistical methods to address uncertainties and reduce biases arising from low-resolution data (e.g., gap filling^29,30^). However, such approaches may only partly compensate for low temporal data resolution, particularly given that obtaining accurate temporal trends can require consistent monitoring for at least a decade^31,32^ or even longer^28,33–36^.

While collecting higher-resolution data would likely reduce bias in biodiversity trend estimates, this does not necessarily mean that conclusions drawn from low-resolution data are incorrect. For instance, several studies show that trends quantified from lower-resolution time series can still broadly match those from higher-resolution data^37–39^, and can detect general differences among sites^40^ and periods^41^. Additionally, some biodiversity studies have revisited their results to determine the influence of data limitations on estimated trends and found no substantial effect^42,43^. Conclusions from studies using temporally limited datasets have also been supported by more robust research in recent years (see similar conclusions between ref. ^14^ and ref. ^44^). These points suggest that certain data limitations may be acceptable without biasing biodiversity trend estimates. However, systematic research is still needed to determine the influence of different data limitations, to inform which are and are not acceptable, and to evaluate the validity of broad-scale biodiversity trend research, thereby helping delineate minimum data requirements for future research, monitoring, and management.

Here, we test how different temporal data limitations affect biodiversity trend estimates using European-scale data for 1353 river invertebrate communities sampled almost annually for 10–29 years across 18 countries. This dataset presents a unique opportunity to investigate the effect of temporal limitations on biodiversity trends, given the general scarcity of biodiversity data that combines community-level information across a broad spatial scale with high temporal resolution. Additionally, freshwater ecosystems tend to be underrepresented in global biodiversity datasets (with some exceptions^14,44^), and conservation in general^45^, thus our study provides insights into how temporal limitations affect biodiversity assessments in a generally underrepresented realm.

Using the annual time series for each site, we simulate lower sampling frequencies, shorter sampling durations, and combinations of lower frequency and shorter duration. We then compare how these changes alter site-by-site and across-site estimates of linear biodiversity trends. Next, we supplement our simulated analyses with an examination of how real temporal differences in monitoring data affect biodiversity trends. We compare trends for 485 site pairs across 8 European countries between two monitoring datasets that differ in temporal resolution: one collected for this study and one from the European Environment Agency (EEA). Our results show how infrequent or short-duration sampling can misrepresent biodiversity trends, providing empirical evidence of the value of long-term, high-resolution data. We further discuss the implications of these findings for broad-scale biodiversity monitoring (e.g., as part of the KM GBF or EU Water Framework Directive), which must consider how best to allocate limited resources to obtain accurate perspectives of biodiversity change.

## Results

### Influence of temporal resolution on biodiversity trends

We examined the influence of lower sampling frequency and duration on trends in four metrics that can indicate change in community diversity or composition: (1) abundance (total number of individuals); (2) richness (total number of taxa); (3) coverage-based richness (here the expected number of species if 80% of each community was sampled); and (4) ecological quality, quantified as the Ecological Quality Ratio (EQR). Ecological quality is a widely used European measure of anthropogenic impacts on freshwater communities^46,47^ based on the compositional similarity of sensitive and tolerant taxa to those from least-impacted reference communities in each region. We quantified trends in the above metrics for 1353 European river sites using annual data derived from nearly annual sampling with some gap filling (see “Annual time series” section). We then compared these trends to those from simulated monitoring schemes with lower sampling frequency, lower duration, or both lower frequency and duration. The frequency simulations were performed using the complete time series for each site, which ranged from 10 to 29 years, whereas the duration simulations used only the first 10 years of data, as this was the only comparable duration across all sites. We present results for abundance, richness, and EQRs in the main text, whereas those for coverage-based richness are provided in Supplementary Figs. 1 and 2 because they consistently mirrored the richness results.

**Fig. 2:**
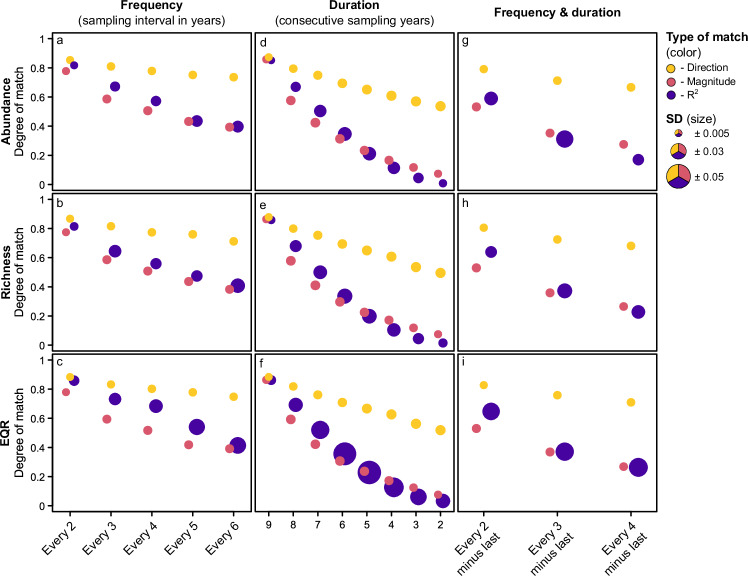
Influence of lower sampling frequency and duration on biodiversity trends. Lower **a**–**c** sampling frequency, **d**–**f** sampling duration, and **g**–**i** a combination of lower frequency and duration increased error rates in biodiversity trends, based on declines in the degree of match between site-by-site trends from the simulated monitoring schemes and those from the complete, annual time series (or first 10 years for duration alone). The degree of match is shown as the mean and standard deviation (SD; circle size) across all simulations for trend direction matches (yellow circles), trend magnitude matches (pink circles), and *R*^2^ of the trend relationships (blue circles; based on generalized linear mixed models) for (**a**, **d**, **g**) abundance, (**b**, **e**, **h**) richness, and (**c**, **f**, **i**) Ecological Quality Ratios (EQRs). Source data are provided as a Source Data file.

Across all community metrics, decreasing sampling frequency increased the site-level trend ‘error rate’, meaning a decrease in the degree to which estimated trend values for each site matched those obtained from annual sampling frequencies for the same sites. For example, decreasing sampling from annual to once every 2–6 years resulted in trends that matched the direction (i.e., positive or negative) of the annual trends in, respectively, 87–73% of sites (percentages reflect the average across metrics; Fig. 2a–c). Compared to the direction matches, error rates increased more severely for the magnitude matches, determined as whether simulations fell inside the standard error of the annual trends, with matches decreasing from 78 to 39%. Likewise, the *R*^2^ of the relationship between the simulated and annual trends (i.e., whether the simulated trends accurately captured among-site trend variation) also declined substantially as frequency declined, from 0.83 to 0.41. These values reflect the potential to mischaracterize biodiversity change in many individual sites, even for sampling frequencies that exhibited the least error. For example, the highest match percentage (87% for direction at a frequency of every 2 years) still corresponds to obtaining the wrong trend direction for 176 sites out of 1353 total. Additionally, we can infer that the more severe increases in magnitude error rates (and declines in *R*^2^) as frequency declined were driven by large magnitude shifts that were in the same direction as the annual trends, given that trend directions were less affected, meaning the magnitude errors were generally not caused by positive trends shifting to negative or vice versa. The magnitude shifts also had to be large to fall outside the standard errors of the annual trends. However, the increase in error rate for magnitude and *R*^2^ exhibited an apparent asymptote whereby error no longer increased as severely once frequency declined to around once every 6 years (e.g., compare the higher degree to which matches declined from every 4 to every 5 years versus every 5 to every 6 years in Fig. 2a–c). This pattern suggests that, after sampling frequency has declined to a certain point, further data loss will not have as strong an influence on the error rate.

The influence of decreasing sampling duration on error rates was generally similar to that of decreasing sampling frequency. Matches between trends from 10-year time series and trends estimated using lower durations of 9–2 years decreased as duration decreased, particularly for magnitude and *R*^2^ (Fig. 2d–f). For example, in comparison to 10-year trends, the direction of 9-year trends matched for 88% of sites (averaged across metrics), but it decreased to 52% when using 2-year trends. Matches for magnitudes and *R*^2^ decreased more severely, from 86% to 8% for magnitude, and from 0.86 to 0.02 for *R*^2^. We also found a similar asymptote for the duration error rate as we observed for frequency, with error rates reaching close to 0 around a total duration of 4 years and thus being unable to decline much further when duration declined to a total of 3 or 2 years.

Combining lower sampling frequency with lower duration (performed by removing the final data point in each frequency simulation) led to further increases in error rates, particularly for magnitude and *R*^2^. These differences were most evident when compared to the individual frequency results (compare Fig. 2a–c, g–i). For example, sampling every 4 years resulted in an average direction match of 78%, which decreased to 68% when combined with reduced duration. In contrast, the magnitude matches and *R*^2^ were more severely affected, decreasing from an average magnitude match of 51% to 27% and from *R*^2^ values of 0.60 to 0.22.

Reducing sampling frequency, duration, or both also tended to increase the *R*^2^ standard deviation (SD), whereas the SD for direction and magnitude showed minimal variation (Fig. 2). Higher SD indicates less consistency among simulations, meaning the degree to which the simulated trends captured the same, among-site variation as the ‘complete’ trends (i.e., annual or 10-year) varied more among simulations compared to trend directions and magnitudes. This effect was most obvious for EQRs at the lowest sampling frequencies (e.g., every 5–6 years; Fig. 2c) and at intermediate durations (e.g., 4–6 years; Fig. 2f). Note, however, that the R^2^ SDs became smaller at the shortest durations because these simulations consistently explained close to 0% variation.

### Across-site differences in trend estimates

**Fig. 3:**
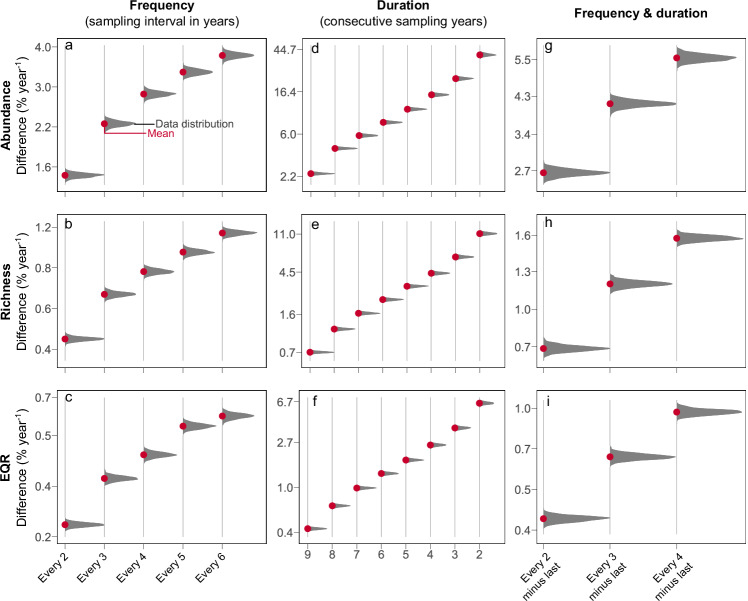
Influence of lower sampling frequency and duration on differences in biodiversity trends. Lower **a**–**c** sampling frequency, **d**–**f** sampling duration, and **g**–**i** a combination of lower frequency and duration tended to increase the differences in trend values between the simulated monitoring schemes and those from the complete time series (i.e., annual or the first 10 years for duration alone). Trend differences are shown using density plots of the average across-site absolute difference for each simulation (shaded polygons) and the average of these differences across simulations (red circles). Note that all y-axes are on the log-scale and that the range of this scale varies across panels. Source data are provided as a Source Data file.

Lower sampling frequency and duration also led to greater error in biodiversity trend estimates, represented as the average of the absolute difference between the simulated and complete trend estimates (i.e., % change per year in abundance, richness, or EQR). This comparison assesses, across all sites, exactly how much the simulated trends deviated from those derived from the complete time series, complementing the direction, magnitude, and *R*^2^ results presented above. Reducing sampling frequency, duration, or both increased the differences between the simulated and complete trends, particularly for abundance and for the duration simulations (e.g., see Fig. 3d). For example, the average, across-site estimates of biodiversity change from the annual time series were 0.37%, 0.79%, and 1.11% year^−1^ for abundance, richness, and EQR, respectively. When sampling frequency was reduced from annual to every 2 years, the simulated trends differed only slightly, by an average of 1.55%, 0.44%, and 0.27% year^−1^, respectively (Fig. 3a, b, c). However, the degree of error became more pronounced as sampling became less frequent, with sampling every 6 years corresponding to average differences of 3.80%, 1.10%, and 0.64% year^−1^. To help contextualize these errors, overall biodiversity change across realms or taxonomic groups often falls within just 0–2% per year^10,14,44^, meaning deviations of even 1% can translate to a large misestimate of the overall degree of biodiversity change.

The difference between trend estimates was relatively small when duration was shortened by just a few years, but it became more pronounced as duration decreased further. For example, the average, across-site trend estimates for the first 10 years were 1.56%, 1.14%, and 1.44% year^−1^ for abundance, richness, and EQRs, respectively. Sampling for 9 years produced estimates that deviated from these 10-year values by average differences of 2.37%, 0.67%, and 0.40% year^−1^, respectively (Fig. 3d–f). However, decreasing the sampling duration to just 2 years resulted in dramatically larger differences of 39.35%, 11.12%, and 6.49% year^−1^.

When combining lower frequency and duration, the differences between the simulated and complete trends tended to increase, particularly compared to the lower frequency simulations alone (compare Fig. 3a–c, g–i). For example, the differences for the 4-year frequency simulations were, on average, 2.84%, 0.79%, and 0.47% year^−1^ (left panels in Fig. 3) for abundance, richness, and EQRs, respectively. When the 4-year frequencies were combined with shorter duration, these differences increased to 5.53%, 1.61%, and 0.97% year^−1^ (right panels in Fig. 3).

While our dataset provides broad spatial coverage, many sites are represented by time series of only ten years in length. To test whether longer time series are more robust to data loss, we repeated our analyses for the subset of 255 sites out of 1353 total with at least 20 years of data, and expanded the duration simulations to 2–19 years in comparison to the first 20 years. We found the same overall results (Supplementary Figs. 3–5), indicating our conclusions were generally consistent whether shorter or longer time series were used. A notable exception was the lower sensitivity of *R*^2^ to declines in frequency and duration. Specifically, changes in *R*^2^ tended to mirror changes in the magnitude matches in our full analyses (Fig. 2). However, when limited to time series with at least 20 years of data, *R*^2^ did not decline as sharply as magnitude (Supplementary Figs. 3 and 4).

### Comparing trends from two European datasets

To support our results using simulated monitoring schemes, we investigated how real temporal differences between two biodiversity datasets influenced estimated trends. To do so, we compared trends in EQR data collected from 2010 to 2023 between two large European monitoring datasets: 485 sites from our annual river invertebrate time series, hereafter referred to as the ‘European River Invertebrate Time Series’ (ERITS), and 485 sites from the river invertebrate dataset of the European Environment Agency (www.eea.europa.eu/data-and-maps/data/waterbase-biology), hereafter referred to as the EEA dataset. We focused on EQRs because the EEA dataset does not provide data on abundance nor richness. We compared trends between paired, proximate sites based on direction matches, magnitude matches, *R*^2^, and differences as above, and based on the density distribution and the across-site average and SD of their trend values. We ensured that the paired ERITS and EEA sites were located in the same rivers, were spatially proximate (average distance of 3.1 m), and that the sampling years for the EEA sites always fell within the sampling years of their paired ERITS sites. On average, the ERITS sites were sampled every 1.3 years across a total duration of 8.7 years, resulting in an average of 6.8 years of data per site. In comparison, the EEA sites were sampled on average every 1.1 years across a total duration of 2.8 years, resulting in an average of 2.6 years of data per site.

**Fig. 4:**
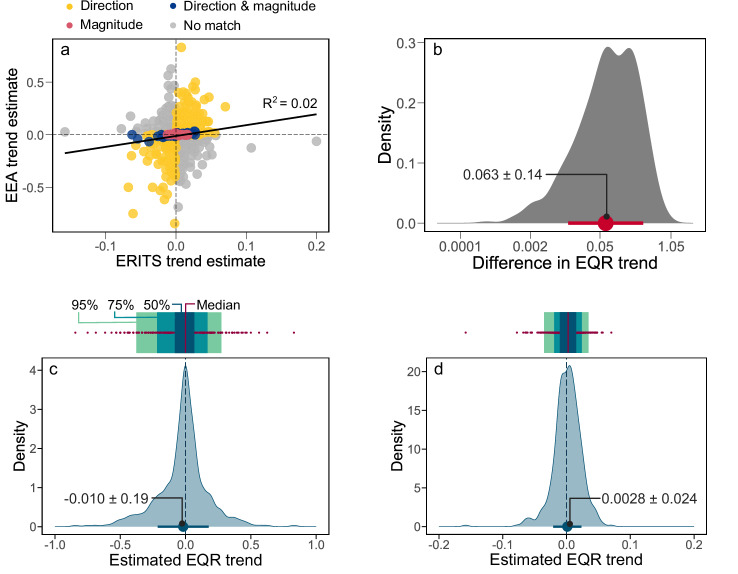
Comparison of estimated EQR trends between two datasets that differ in temporal resolution. Differences in temporal resolution between the EEA and ERITS site pairs (*n* = 485) influenced: **a** matches between the direction and magnitude of site-level ecological quality trends (EQR change year^−1^), with the fitted regression line and the corresponding *R*^2^ from the GLMM; **b** trend estimates, represented as the absolute differences between the EEA and ERITS slope values; and **c**, **d** the average, across-site estimates of ecological quality change. Density plots in (**c**, **d**) show the probability distribution of trend estimates for the 485 sites. Dots and error bars represent the mean and standard deviation. Box plots show the median (center line), the 25th–75th percentiles (50% of the data), with extended boxes indicating the 12.5th–87.5th (75%) and 2.5th–97.5th (95%) percentiles. Red dots show data outside the 25th–75th percentile range. Source data are provided as a Source Data file.

Trend directions matched for 258 out of 485 site pairs (53%; Fig. 4a), whereas trend magnitudes only matched for 57 site pairs (12%). Moreover, the EEA slopes only captured 2% of the variation in the ERITS slopes (*R*^2^ = 0.02), indicating minimal agreement between datasets that differ primarily in temporal resolution. Furthermore, EQR trend estimates differed between site pairs by an average of 0.063 year^−1^ (note this is change in the raw EQR values and not transformed to % year^−1^; Fig. 4b). This difference translates to a misestimate of change in community similarity to reference conditions by up to 0.6 (60% similarity) over a decade, which is over half the total EQR range of 0–1, reflecting a substantial potential to mischaracterize the degree to which a site has degraded or recovered. The density distribution of the EEA trends was also noticeably wider compared to the ERITS trends (note the narrower scale on the x-axes and the smaller size of the interquartile ranges in Fig. 4c versus d), indicating a wider range and greater variation in trend values in the EEA dataset. Additionally, while the average, across-site estimate of biodiversity change was similar between datasets (0.0030 ± 0.024% year^−1^ for ERITS and −0.010 ± 0.19% year^−1^ for EEA), their signs may differ, with the positive average trend in the ERITS dataset indicating slight temporal improvement in ecological quality, whereas the negative average trend in the EEA dataset indicated slight degradation.

## Discussion

Our results indicated that robust estimates of both biodiversity trend direction and magnitude required time series with a high temporal resolution, both in terms of frequency and duration. Such robust estimates are essential when providing specific recommendations for conservation and management. For example, accurate estimates of the extent or rate of biodiversity change are needed to evaluate progress towards global conservation targets, such as determining whether losses have been reduced to near zero in areas of high biodiversity importance (i.e., Target 1 of the Kunming-Montreal Global Biodiversity Framework; CBD 2022). Additionally, accurate magnitude estimates are important for comparing the degree of biodiversity loss among regions or ecosystems^10,14^, thus informing the urgency of action and helping target conservation actions and resource allocation to communities experiencing the greatest losses^48,49^. The importance of accurate estimates and their applications underscores the value of high-resolution temporal data and echoes repeated calls for more long-term ecological research^32,34^.

We also found that even moderate limitations in temporal resolution led to incorrect trend estimates for many individual sites. In our dataset of European river invertebrate communities, for example, the least limited simulations (i.e., frequency of every two years or duration of nine years in a 10-year time series) still resulted in trend mismatches for around 10–20% of sites, which were further magnified when frequency and duration limitations were combined. Similarly, the same simulations led to estimates of biodiversity change that were incorrect by up to 4% year^-1^, equivalent to 40% per decade. Considering the generally small magnitude of overall biodiversity change often reported across realms or taxonomic groups (e.g., 0–2% per year^10,14,44^), such inaccuracies are substantial and may lead to erroneous decisions. For instance, inaccurate estimates might indicate that biodiversity in a particular community is stable or increasing when it is not, resulting in a lack of conservation actions where needed. Conversely, if biodiversity is mistakenly concluded to be declining when it is not, then limited resources may be misallocated to communities of less concern. These errors could lower credibility with stakeholders, thus potentially compromising future research and management that requires and benefits from stakeholder engagement^50^. Consequently, although a particular biodiversity dataset may be deemed more robust (e.g., having more than just a few years of data collected over a short time period), any consistent gaps may warrant caution when using such data to make management and conservation decisions for individual sites.

Differences in ecological quality trends between the ERITS and EEA datasets further highlighted how the temporal resolution of biodiversity data can affect real-world management decisions. Although both datasets sampled proximate sites from the same rivers experiencing the same stressors, they exhibited markedly different site-level trend directions (53% match), magnitudes (12% match), and differences in EQR trend estimates (average of 0.063 year^−1^). While differences in the overall outcomes were minimal, their potentially contrasting directions and the generally differing perspectives of change between individual site pairs indicated the potential for data temporal resolution to influence local-scale management recommendations. For example, site-level ecological quality data reported as part of the EU Water Framework Directive (WFD) provide periodic updates on progress towards achieving a ‘good’ ecological status for European waterbodies. Within this framework, where site-level assessments feed into regional and continental evaluations, trend discrepancies risk misdirecting local interventions and biasing broader status reports. We recognize that the subset of sites used in our comparison is not comprehensive, as it includes only eight European countries, 32% of the total ERITS sites, and 4% of the total EEA sites. However, our goal is not to provide a complete assessment of European river status, nor to argue for the validity of one dataset over another. Instead, we seek to highlight the critical need to consider temporal resolution in biodiversity datasets because of its potential to affect management recommendations, which impacts future conservation actions and resource allocation.

Despite the recognized value of high-resolution temporal data^34^, we do not want to be misconstrued as implying that low-resolution data has no value, particularly because they are often the only data available. Our results suggest that the direction of trends obtained from such data is relatively robust to low sampling frequency and duration, which makes sense because it is more difficult for data gaps to entirely reverse the direction of a trend compared to altering its exact value. Direction estimates offer important insights into overall biodiversity status because they indicate whether biodiversity has generally increased or decreased across sites^7,8,12^, thereby informing research, policy, strategic coordination, and planning^51^. Such insights can also help identify general biodiversity responses to broad-scale stressors like urbanization or climate change^44,52^, and assess whether broad-scale legislation or conservation actions have effectively halted declines^53,54^. Thus, while limited in resolution, these data and studies can meaningfully progress our understanding of historical biodiversity change.

Our study addresses how linear trends in river invertebrate time series collected annually over at least a decade compare to those with lower temporal resolution. This approach uses a benchmark that is typically of a higher resolution compared to a variety of broad-scale biodiversity datasets and monitoring schemes. However, depending on the taxonomic group and ecosystem in question, biodiversity change may be better represented by non-linear dynamics (e.g., in ecosystems that experienced a regime shift^55,56^), annual sampling may not be necessary^37^, and multiple decades of monitoring may be needed to fully represent biodiversity change^32,35,36^. Lastly, our analyses did not consider other important sources of uncertainty that commonly affect biodiversity data. Such sources include whether the underlying data is even suitable for trend analysis, referred to as ‘indirect’ uncertainties (*sensu* ref. ^57^). Indirect uncertainties can arise for numerous reasons, such as datasets aggregating information from incomparable study designs or sampling methods^16–18,23^, or combining taxonomic groups with different population growth patterns^20,26,58^. Our dataset provided a valuable opportunity to minimize these issues because sites used similar methods to sample the same general taxonomic group from the same ecosystem type (i.e., invertebrates sampled from river bottoms). However, we acknowledge the need to evaluate and control indirect uncertainties in other datasets, taxonomic groups, and realms, and for biodiversity research and monitoring in general.

Our findings have broader practical implications for future monitoring and general implications for our ecological understanding of biodiversity change. From a practical standpoint, our study can help inform and design temporal global monitoring efforts (e.g., GBiOS^59^, or see elter-ri.eu), which must balance accurate trend estimation with realistic constraints in funding, time, and personnel. For example, our results suggest that programs limited to low-resolution monitoring can still obtain an accurate, broad-scale overview of trend directions, but may fail to accurately estimate the rate of biodiversity change over time. We also found that trend estimates were less affected by declines in frequency than by duration, as evidenced by higher site-by-site matches, higher explained variances, and lower differences in the frequency compared to the duration simulations. We acknowledge that the duration and frequency results are not always directly comparable because the former was restricted to 10 years, whereas the latter could encompass longer time periods, but some generalizable differences were still apparent. These differences likely reflect the greater sensitivity of linear temporal trends to the loss of either the initial or final years of a time series^26,60^. Reducing frequency still maintains coverage of both the early and late years, whereas reducing duration excludes either the earlier or later years entirely. Thus, reducing sampling frequency may be a cost-saving measure that provides higher trend accuracy compared to reducing sampling duration. Efficient planning of the temporal monitoring scheme can be further complemented by systematic planning in other aspects, including sampling network design^61,62^, choice of taxonomic resolution^63,64^, tools to estimate the minimum required sampling effort^65–67^, or the implementation of more cost-effective approaches (e.g., eDNA and metabarcoding^68,69^). Additionally, if logistical or financial constraints preclude higher-resolution monitoring, then complementary statistical approaches may help mitigate these issues, including gap filling^29^ (as we used) or model-based methods to improve trend estimates from sparse data (e.g., hierarchical^70^ or state-space^71^ models). However, these approaches can also inflate errors in some trend estimates^72^.

In addition to planning, our results can help estimate uncertainty in already collected monitoring data, which can then be addressed or at least clearly communicated. For example, biodiversity data collected to meet the requirements of the EU WFD can have three- to six-year gaps, which our results suggest could lead to up to 60% uncertainty in site-level estimates of change and introduce large errors in estimates of the overall percent of sites that have improved or deteriorated compared to previous years. An increase in the EU WFD sampling frequency to every two years would reduce the current uncertainty from between 40% (every 3 years) and 60% (every six years), down to around 20%. If gathering additional data is not feasible, then clear and transparent communication of the current degree of uncertainty can help inform conservation actions and build trust among stakeholders and decision-makers^49^.

Regarding general ecological implications, our results outline potential strengths and weaknesses in current global- and continental-scale research on biodiversity change across freshwater, marine, and terrestrial ecosystems. We show that trend directions can be robust to moderate limitations in temporal resolution, although more severe limitations can substantially alter estimates. This lower sensitivity was consistent across metrics of abundance, richness, coverage-based richness, and ecological quality, suggesting it may apply to other measures of biodiversity or ecological status, although this requires further confirmation. Consequently, trends from broad-scale studies reporting an equal mixture of negative, positive, and neutral biodiversity changes^7–10,14^ may indeed be accurate, with some areas experiencing losses, whereas others are unaffected or even gain biodiversity due to factors such as recovery^13,73^, species range shifts^11^, and non-native introductions^8,9^. However, our findings also emphasize that trend magnitudes are highly sensitive to temporal data resolution. This higher sensitivity suggests that many of these same studies may provide incorrect estimates of the exact magnitude of change and could misestimate trends for many individual sites, potentially making them unsuitable for informing targeted management.

Data quality determines the lens through which we view biodiversity change. Temporal resolution is a principal component of this quality; thus, identifying the most appropriate resolution, and the limitations of current data, are key aspects in obtaining the most accurate perspective. Our study aids these efforts by providing researchers and stakeholders with a framework to select optimal monitoring frequencies and durations that meet defined uncertainty criteria. We also outline the potential for error when using low-resolution time series for anything beyond broad-scale assessments of the directions of change. These contributions help guide effective research, monitoring, and conservation actions aimed at bending the curve of biodiversity loss.

## Methods

### Annual time series

We obtained a total of 4050 time series of European river invertebrate communities from ref. ^74^ and ref. ^75^, and from further requests to ecologists and environmental managers. Our data requirements were: (1) abundance and community composition information was available for quantifying different community metrics; (2) at least seven individual years of data were available spanning at least a decade, with a decade typically encompassing several generations of change in most freshwater invertebrates (which have life cycles ranging from days to years^76^, but a majority are less than 1 year^77^); (3) all samples within a site were collected during the same season, defined as any three consecutive months; and (4) sampling methods and taxonomic resolution were consistent within each site across years. In some countries, identification levels were inconsistent through time, and in these cases, we used the most temporally consistent level. For example, if a taxon was initially identified to the family level and then later to the genus or species level, we raised all identifications of this taxon to the family level. We refer to all time series data that meet our criteria as the ‘European River Invertebrate Time Series’ (ERITS) dataset.

To produce annual time series for quantifying biodiversity trends, we selected time series from the ERITS dataset that were sampled generally annually but may have missing data in some years, which is the best quality data typically available. To address concerns with the amount of missing data, we further selected sites where missing data occurred in non-consecutive years and with a maximum of 33% missing data (e.g., a maximum of 3 non-consecutive missing years in a 10-year time series or 6 years in a 20-year series). Our inclusion criteria provided 1353 sites from the ERITS dataset encompassing 18 countries (Supplementary Fig. 6a). These time series spanned an average total duration of 15.9 years with an average of 14.6 sampled years. We refer to this subset of 1353 sites as the ERITS annual time series (see Supplementary Table 1 for further site information). Taxa in 56% of these sites (761 out of 1353) were identified only to the family level or higher, with 44% identified to a mixed resolution, generally a combination of families, genera, and species, although the exact resolution can vary among countries. Such mixed-level identifications are common in invertebrate research, where many taxa cannot be readily identified to the species or even genus level. Excluding sites with only family-level or higher identifications did not influence our results (Supplementary Figs. 7 and 8), indicating our findings were consistent whether we did or did not restrict our dataset to sites with only finer-scale taxonomic information.

We represented biodiversity for each site and year based on three community metrics: (1) abundance (total number of individuals); (2) richness (total number of taxa); and (3) ecological quality. Temporal changes in these metrics are, however, potentially affected by the need to adjust sampling effort, such as sites or periods with greater abundance requiring greater sampling effort to collect the same proportion of taxa as communities with lower abundance^78^. Ecological quality can suffer from similar biases as it can include metrics of abundance and richness in its calculation (detailed in Supplementary Table 2). To partly test for these issues, we also quantified coverage-based richness for each site and year, which estimates taxon diversity if each sample was collected to the same degree of completeness. We calculated coverage-based richness using the iNEXT package in R^79,80^ with coverage set to 80% and *q* = 0. Ecological quality was measured as the Ecological Quality Ratio (EQR) of each invertebrate community, calculated using country- and river-specific methods and software (Supplementary Table 2). After calculating all community metrics, we interpolated the values for missing years by averaging values from the previous and following years.

### Influence of reduced sampling

To evaluate how reducing sampling affected biodiversity trend estimation, we simulated time series with lower frequency, shorter duration, or a combination of both, and compared them to the complete time series. We calculated trends by fitting linear models of abundance, richness, coverage-based richness, or EQRs in relation to year. We initially tested for significant (*P* < 0.05) temporal autocorrelation between successive years within sites using the DHARMa package^81^ but only found evidence for autocorrelation in 6.9–8.6% of sites (depending on the metric), so we did not control for this in our final models. All slopes were converted to percent change per year by log-transforming the metrics prior to modeling, then exponentiating the slopes and subtracting 1. This conversion ensured that slopes were comparable among sites regardless of differences in abundance units, taxonomic resolution, or total richness.

**Fig. 5:**
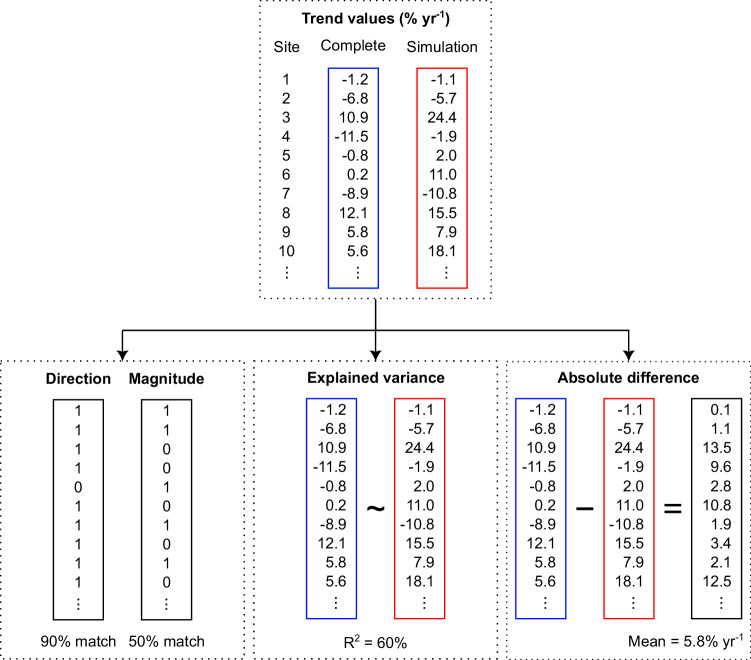
Illustrative example of how the simulation results were produced. Methods used to quantify the trend direction and magnitude matches, *R*^2^, and differences for each of 1000 simulations for the different frequency, duration, and frequency & duration simulations. Direction matched (0 = no match, 1 = match) if the ‘complete’ trends (i.e., trends from the annual time series or the first 10 years for duration alone) and simulated trends had the same direction. Magnitudes matched if the simulated trends fell within the standard error. *R*^2^ reflects the proportion of trend variance captured by the simulations, quantified using GLMMs. Differences represent the average deviation in the simulated trend values, quantified as the across-site geometric mean of the absolute differences.

We first compared the complete versus simulated slopes using a vote-counting method whereby we compared the proportion of sites for which the simulated trends matched the direction or magnitude of the complete trends (Fig. 5). We considered the directions as matching if the complete versus simulated slopes had the same sign (i.e., positive or negative). Magnitudes matched if the simulated slope was within the standard error of the complete slope, which allowed for slight changes in direction to still match in magnitude. We could not compare slope significance because many frequency simulations provided only two years of data.

In addition to direction and magnitude matches, we related all the complete, site-level slopes to the simulated slopes to determine the degree to which variation in the annual slopes was reflected in the simulated slopes (i.e., the *R*^2^ relationship shown in Figs. 1c and 5). We did so using generalized linear mixed models (GLMM) performed with the glmmTMB package^82^. All models included a random intercept term for ‘country’ to control for potential non-independence among slopes within the same countries, and we extracted the marginal *R*^2^ of these relationships using the MuMIn package^83^. Furthermore, we compared differences between the complete and simulated slope estimates by taking the absolute value of the difference for each site in each simulation and calculating the geometric mean of these differences across sites. We used the geometric mean to reduce the influence of a small number of sites with extreme trend estimates.

We simulated different sampling frequencies by only including data from every 2–6 years for each site (see Table 1 for a summary of analyses). Given we set the minimum time series duration to ten years, these frequencies meant that each simulation included at least two years of data to quantify trends. We chose these frequencies because they span a range commonly used in broad-scale monitoring programs (e.g., the EU Water Framework Directive recommends minimum sampling frequencies of every 3–6 years). For each site, we randomly selected whether sampling began in the first or second year of each time series and repeated this process 1000 times.

**Table 1:** Summary of analyses

Analysis	Dataset(s)	Comparison
Frequency	ERITS annual time series (*n* = 1353)	Trends for the annual time series versus only using data from every 2–6 years
Duration	ERITS annual time series (*n* = 1353)	Annual trends for the first 10 years versus only using 2–9 years of data
Frequency and duration	ERITS annual time series (*n* = 1353)	Trends for the annual time series versus only using data from every 2–4 years and removing the last year of data
Comparison between datasets	ERITS (*n* = 485) versus EEA (*n* = 485)	Trend comparison between two datasets with different temporal resolutions

We simulated shorter sampling durations by quantifying trends for the first 10 years in each annual time series, then re-quantifying these trends using only 2–9 years of the same time series. We focused on the first 10 years because this is the maximum comparable duration across all time series, all of which spanned at least a decade. For each site, we randomly selected the starting year from all possible options within the first 10 years and repeated this 1000 times.

Lastly, we examined the influence of combining reduced sampling frequency with reduced duration by repeating our frequency simulations but removing the last year of data in each, thus reducing duration as well. This approach restricted the simulations to frequencies of only every 2–4 years to ensure all time series provided at least two years of data.

For all the above simulations, we compared slope direction, magnitude, *R*^2^, and trend differences, ensuring that comparisons were always made using slopes quantified from the same starting years.

### Comparing datasets differing in temporal resolution

We supplemented our simulation analyses by examining how real differences in temporal resolution between monitoring datasets affect site-by-site and across-site biodiversity trends (Table 1). We did so by comparing paired, proximate river invertebrate communities from the ERITS dataset to those from the European Environment Agency (EEA; www.eea.europa.eu/data-and-maps/data/waterbase-biology). These datasets differ in their sampling frequency and duration (detailed in the *Results* and in Supplementary Table 3), but both provide time series data collected between 2010 and 2023. To determine the site pairs, we first used a cutoff distance threshold of 1 km to identify sites in the same geographic area. We then selected only site pairs that were the closest to one another and within the same waterbody (average distance between pairs of 3.1 m; range 1.2–114.6 m; Supplementary Fig. 6b).

We followed the same procedures detailed for the simulation analyses to compare slope direction and magnitude matches, *R*^2^, and trend differences among site pairs. Briefly, we first estimated site-level EQR trends. We tested for significant (*P* < 0.05) temporal autocorrelation between successive years within sites and found only weak evidence of such (7.4% of the ERITS sites and 10.9% of the EEA sites), so we did not control for temporal autocorrelation in these models. We then extracted the slope direction, magnitude, and standard error from each model and compared the proportion of sites where slope directions and magnitudes matched between datasets. Numerous sites in the EEA dataset provided only two sampling years, preventing the estimation of standard errors, whereas all sites in the ERITS dataset provided at least three sampling years. Therefore, we used the ERITS slopes as the baseline for direction and magnitude comparisons to the EEA slopes. Additionally, we related the slopes between datasets using a GLMM, which included a random intercept term for ‘country’. From this model, we extracted the marginal *R*^2^ to assess how well variation in the ERITS slopes (i.e., the reference sites) was reflected in the EEA slopes. We also calculated the absolute difference of the trend estimates between each site pair and the geometric mean of these differences.

Lastly, because site pairs spanned a range of distances between one another that could influence the relationship between the EEA and ERITS trend slopes, we included an interaction term for distance (m) between site pairs in the GLMM. We then determined the significance (*P* < 0.05) of this interaction and the individual distance term by comparing models with and without these terms using Likelihood Ratio (LR) tests. The interaction was marginally non-significant (LR test, *L* = 3.73, df = 1, *P* = 0.053), and the individual distance term was also non-significant (LR test, *L* = 1.01, df = 1, *P* = 0.31), indicating that controlling for the distance between site pairs would not substantially affect our results, so we excluded the distance term from the final model.

### Reporting summary

Further information on research design is available in the Nature Portfolio Reporting Summary linked to this article.

## Supplementary information

Supplementary Information

Reporting Summary

Transparent Peer Review file

## Source data

Source data

## Data Availability

The data used in this study are publicly available from Figshare under the accession code 10.6084/m9.figshare.28920428. These data include the time series information for each site, the community metric values for each site and year required to repeat the simulations, and the trend estimates required to repeat the paired ERITS and EEA trend comparisons. Source data are provided with this paper.

## Code availability

All R code needed to repeat our analyses is available from Figshare under the accession code 10.6084/m9.figshare.28920428.
