# Supplementary material for: Robust estimates of biodiversity change require high-resolution time series
Source: Nat Commun. 2026 Jul 7;17:5941. doi: 10.1038/s41467-026-75321-0 (PMC13342590; doi:10.1038/s41467-026-75321-0)
Supplement: Supplementary file 1 — Supplementary Information [file 41467_2026_75321_MOESM1_ESM.pdf]

Robust estimates of biodiversity change require high-resolution time series

Supplementary Information

Supplementary Tables

Supplementary Table 1: Descriptors of time series by country used to test the influence of temporal limitations in biodiversity data.

Country	No. Sites	Initial year	Final year	No. Sampling years	Time series length	Lag between sampling years
Belgium	12	1994	2022	9.08	11.83	1.34
Bulgaria	1	2010	2019	9.00	10.00	1.13
Czechia	26	2006	2021	12.08	14.42	1.21
Denmark	193	1992	2020	20.80	22.32	1.08
Finland	24	2000	2021	12.96	13.96	1.08
France	265	1992	2022	10.00	12.23	1.25
Germany	14	1992	2019	11.57	12.50	1.09
Hungary	72	2005	2019	11.22	12.04	1.08
Ireland	16	2003	2019	16.81	16.81	1.00
Latvia	3	1996	2015	19.00	19.67	1.04
Lithuania	1	2010	2020	8.00	11.00	1.43
Luxembourg	20	2007	2017	10.00	11.00	1.11
Netherlands	8	1992	2019	16.88	17.75	1.06
Norway	22	2003	2020	13.55	16.59	1.24
Portugal	2	1993	2019	27.00	27.00	1.00
Spain	140	1992	2021	17.35	17.81	1.03
Sweden	85	1995	2019	14.35	14.59	1.02
UK	449	1994	2019	15.09	16.18	1.08

Supplementary Table 2: References to the methods, which can require interfacing with country-specific software, used to produce the Ecological Quality Ratios (EQRs).

Country	Method	Metrics	Method reference
Belgium	Multimetric Macroinvertebrate Index Flanders	(1) Total taxon richness; (2) number of EPT (Ephemeroptera, Plecoptera and Trichoptera); (3) Shannon–Wiener diversity; (4) mean tolerance score (taxa scored from 1-10 with lower values indicating pollution tolerant taxa); and (5) the number of sensitive taxa (number of taxa with tolerance score >5 not including EPT).	1
Bulgaria	Biotic Index	Taxa are scored from 1-5 (lower values for taxa indicative of polluted conditions). The final metric value is based on the relative abundance of taxa with scores 1/2 versus 3/4/5.	2
Czechia	Multimetric index using river type-specific metrics	Included metrics depend on river type. Can include: (1) % EPT; (2) total family richness; (3) Margalef diversity; (4) Czech saprobic index; (5) lithal microhabitat preference; (6) epirhithral, metarhithral, or hyporhithral, and/or epipotamal stream zone preferences; (7) % of different feeding types; (8) SPEAR organic sensitivity index; and (9) reconstructed taxocenoses (REKO) index.	3, 4
Denmark	Danish Streamfauna Index	The presence of six indicator taxa groups categorized by their resistance or sensitivity to organic pollution.	5

Finland	Finnish Multimetric Index	(1) Occurrence of type-specific reference taxa; (2) occurrence of type-specific reference EPT families; and (3) Percent Model Affinity (similarity of community composition to reference composition).	6–8
France	I_2M_2	(1) Total taxon richness; (2) Shannon-Wiener diversity; (3) relative abundance of polyvoltine species; (4) relative abundance of ovoviviparous species; and (5) the Average Score Per Taxon index (ASPT; average score among taxa scored from 1-10, with lower values indicating taxa with greater pollution tolerance).	9
Germany	Multimetric index using river type-specific metrics	Included metrics depend on river type. Can include: (1) the German Fauna Index (taxa assigned scores of -2 to +2, with lower values for taxa indicative of polluted conditions); (2) the Potamon Typie Index (affinity to the potamal zone); (3) the Rhithron Typie Index (affinity to the rhithral region); (4) the Lake Outlet Index (score 1-5 with higher values indicating taxa that prefer lake outlets); (3) % epirhithral taxa; (4) % metarhithral taxa; (5) % epipotamal taxa; (6) % littoral taxa; (7) % pelal taxa; (8) % phytal taxa; (9) ratio of rheophilic and rheobiont taxa; (10) total number of EPT; (11) total number of EPTCOB (EPT plus Coleoptera, Odonata, and Bivalvia); (12) % EPT; (13) % Oligochaeta.	10

Hungary	Hungarian Multimetric Index	Included metrics depend on river type. Always includes the ASPT index. Can include: (1) total number of EPT; (2) ratio of EPT; (3) total number of EPTCOB; (4) Shannon–Wiener diversity; (5) ratio of crenal to hyporhithral stream zonation preferences; (6) ratio of rheophil to rheobiont current preferences; (7) ratio of limno-to-rheophil to rheobiont current preferences.	11
Ireland	Quality Rating System	Taxa are scored from 1-5 (lower values for taxa indicative of polluted conditions). The final metric value is based on the relative abundance of taxa with scores 1/2 versus 3/4/5.	12
Latvia	Latvian Macroinvertebrate Index	(1) Total taxon richness; (2) Shannon-Wiener diversity; (3) number of EPT; (4) Danish Streamfauna Index (DSFI; presence of six indicator taxa groups categorised by their resistance or sensitivity to organic pollution); and (5) the ASPT index.	13
Lithuania	Lithuanian River Macroinvertebrate Index	(1) Richness of Diptera, Ephemeroptera, and Plecoptera; (2) DSFI; (3) the ASPT index; and (4) the difference between the combined relative abundances of Ephemeroptera, Heteroptera, and Plecoptera versus Crustacea and Hirudinea	https://e-seimas.lrs.lt/portal/legalAct/lt/TAD/TAIS.296626/asr; 14
Luxembourg	I_2M_2	(1) Total taxon richness; (2) Shannon-Wiener diversity; (3) relative abundance of polyvoltine species; (4) relative abundance of ovoviviparous species; and (5) the Average Score Per Taxon index (ASPT; average score among taxa scored from 1-10, with lower values indicating taxa with greater pollution tolerance).	9

Netherlands	KRW-maatlatten	The abundance of positive and negative water type specific indicator taxa.	15
Norway	Poorest out of the Average Score Per Taxon index and the River Acidification Macroinvertebrate Index	(1) ASPT index; (2) River Acidification Macroinvertebrate Index, which uses: (i) number of EPT and (ii) taxa scores based on tolerance to low pH.	6
Portugal	South Portugal macroinvertebrate biotic index	(1) Total number of families; (2) number of EPT; (3) the ASPT index (derived from Iberian BMWP scores); (4) total abundance of Chloroperlidae, Nemouridae, Leptophlebiidae, Ephemerellidae, Philipotamidae, Elmidae, Leuctridae, Limnephilidae, Sericostomatidae, Dryopidae and Athericidae.	16
Spain	Basque: Multimetric Basque index family level (MBf index)<br><br>Elsewhere: Iberian Biological Monitoring Working Party (IBMWP)	MBf index: (1) Total number of families; (2) number of EPT families; (3) abundance of a selection of 29 families of Ephemeroptera, Trichoptera, and Diptera; (4) abundance of a selection of 14 families of Ephemeroptera, Plecoptera, Trichoptera, and Diptera; (5) number of families from a selection of 12 families of Ephemeroptera, Trichoptera, and Diptera; and (6) IBMWP adapted to Basque rivers (see below)<br><br>IBMWP: Sum of scores across present families scored from 1–10 based on their known sensitivities to organic pollution, with higher scores for more sensitive families.	17–19
Sweden	Average Score Per Taxon and the DJ index	(1) The ASPT index; and (2) the DJ index, which uses: (i) number of EPT, (ii) % EPT, (iii) and % Crustacea.	6, 20, 21

UK	Whalley Hawkes Paisley Trigg (WHPT) Average Score Per Taxon and number of scored taxa	Families are scored from -1.3–13 based on their known sensitivities to oxygen depletion, with higher scores for more sensitive families. $ASPT_{WHPT}$ is the sum of these scores divided by the number of scoring taxa.	22, 23

Supplementary Table 3: Descriptors of the time series compared between the ERITS versus EEA datasets to assess the influence of differences in temporal resolution.

Country	No Sites	Initial year	Final year	No Sampling years	Time series length	Lag between sampling years
**ERITS**	**485**	**2010**	**2023**	**6.79**	**8.73**	**1.34**
Belgium	8	2010	2023	4.38	10.00	2.67
Bulgaria	1	2010	2019	9.00	10.00	1.13
France	325	2010	2022	6.28	8.59	1.44
Hungary	74	2010	2019	7.55	7.95	1.06
Italy	1	2010	2017	8.00	8.00	1.00
Latvia	1	2010	2015	6.00	6.00	1.00
Netherlands	6	2010	2019	8.50	9.50	1.13
Norway	1	2010	2020	6.00	11.00	2.00
Spain	57	2010	2021	8.44	10.21	1.24
Sweden	11	2010	2019	9.00	9.18	1.02
**EEA**	**485**	**2010**	**2020**	**2.62**	**2.82**	**1.12**
Belgium	8	2010	2017	4.25	4.88	1.19
Bulgaria	1	2011	2018	6.00	8.00	1.40
France	325	2010	2011	2.00	2.00	1.00
Hungary	74	2013	2019	4.89	5.01	1.03
Italy	1	2012	2015	2.00	4.00	3.00
Latvia	1	2014	2015	2.00	2.00	1.00
Netherlands	6	2010	2015	2.50	4.33	2.22
Norway	1	2018	2020	3.00	3.00	1.00
Spain	57	2016	2020	2.32	3.11	1.60
Sweden	11	2010	2018	6.09	7.91	1.36

Supplementary Figures

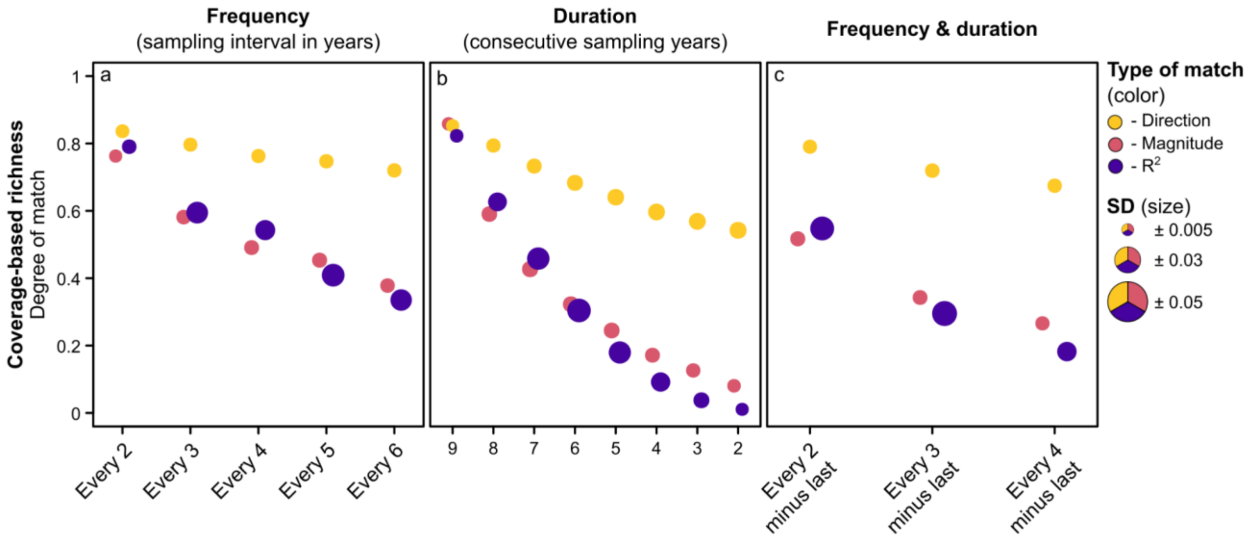

Supplementary Figure 1: Influence of lower sampling frequency and duration on site-level trends in coverage-based richness. Lower (**a**) sampling frequency, (**b**) sampling duration, and (**c**) a combination of lower frequency and duration led to increased trend error rates, based on declines in the degree of match between site-level trends from the simulated monitoring schemes and those from the complete, annual time series (or first 10 years for duration alone). The degree of match is shown as the mean and standard deviation (SD; shape size) of trend direction matches (yellow circles), trend magnitude matches (pink circles), and R^2 of the trend relationships (blue circles; based on generalized linear mixed models). Source data are provided as a Source Data file.

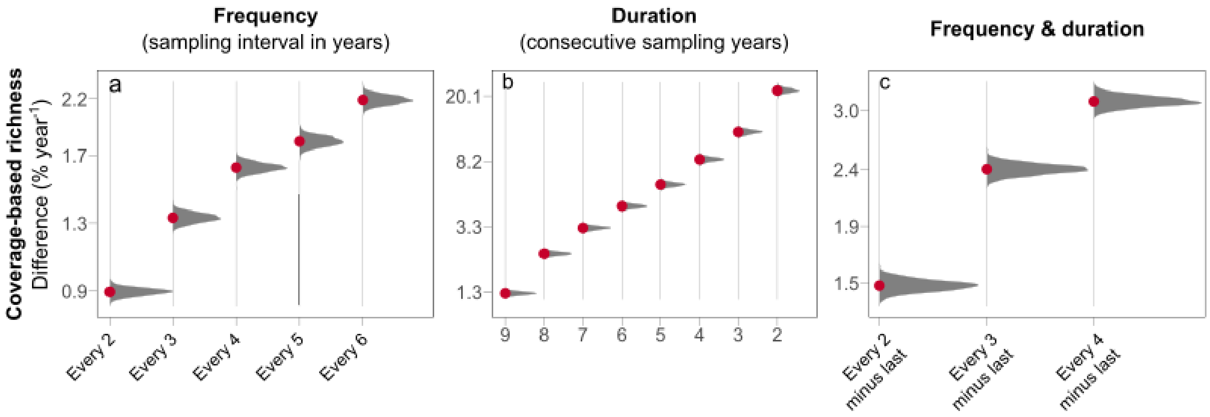

Supplementary Figure 2: Influence of lower sampling frequency and duration on differences in trends of coverage-based richness. Lower (a) sampling frequency, (b) sampling duration, and (c) a combination of lower frequency and duration tended to increase the differences in trend values for coverage-based richness between the simulated monitoring schemes and those from the complete time series (i.e., annual or the first 10 years for duration alone). Trend differences are shown using density plots of the average, across-site absolute difference for each simulation (shaded polygons) and the average of these differences across simulations (filled circles). Note that all y-axes are on the log-scale and that the range of this scale varies across panels. Source data are provided as a Source Data file.

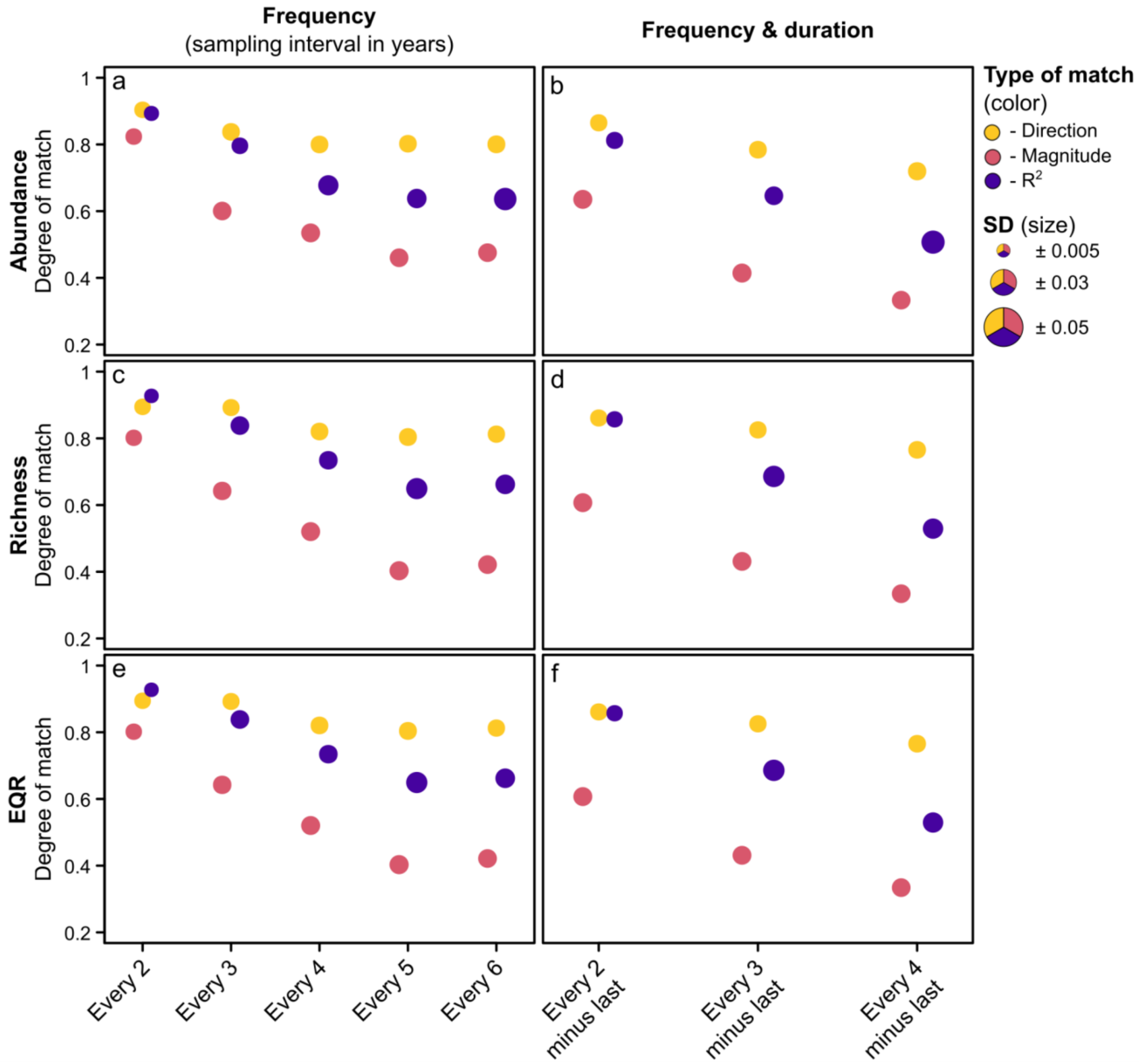

Supplementary Figure 3: Influence of sampling frequency on site-level biodiversity trends for 20-year time series. Lower (**a, c, e**) sampling frequency, and lower (**b, d, f**) frequency and duration, increased error rates in biodiversity trends based on declines in the degree of match between site-level trends from the simulated monitoring schemes and those from a subset of 255 annual time series with at least 20 years of data. The degree of match is shown as the mean and standard deviation (SD; shape size) of trend direction matches (yellow circles), trend magnitude matches (pink circles), and R^2 of the trend relationships (blue circles; based on generalized linear mixed models) for (**a, b**) abundance, (**c, d**) richness, and (**e, f**) Ecological Quality Ratios (EQRs). Source data are provided as a Source Data file.

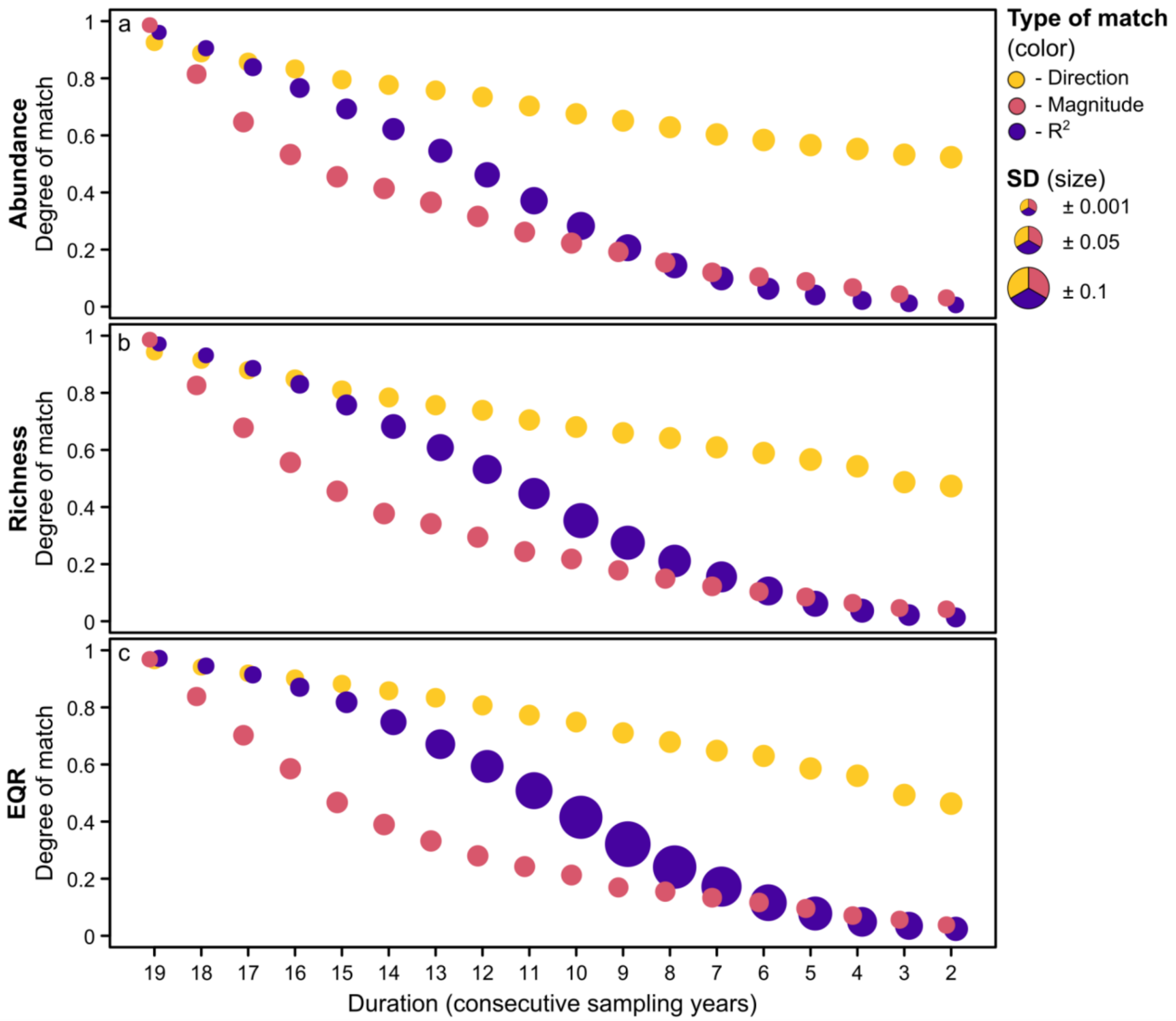

Supplementary Figure 4: Influence of sampling duration on site-level biodiversity trends for 20-year time series. Lower sampling duration increased error rates in biodiversity trends, based on declines in the degree of match between site-level trends from the simulated monitoring schemes and those from a subset of 255 annual time series with at least 20 years of data. The degree of match is shown as the mean and standard deviation (SD) of trend direction matches (purple circles), trend magnitude matches (orange), and R^2 of the trend relationships (black; based on generalized linear mixed models) for (**a**) abundance, (**b**) richness, and (**c**) Ecological Quality Ratios (EQRs). Source data are provided as a Source Data file.

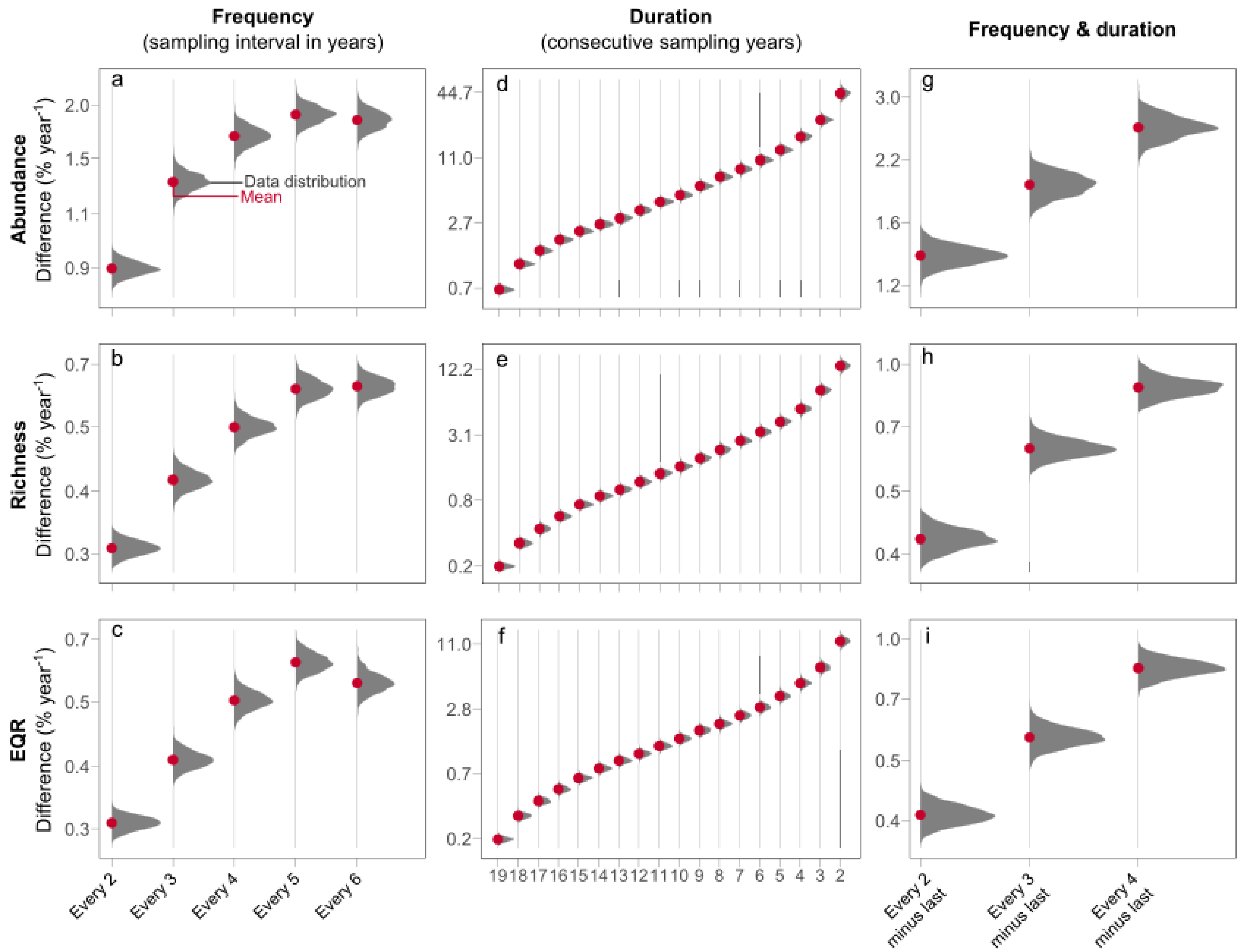

Supplementary Figure 5: Influence of lower sampling frequency and duration on differences in biodiversity trends for 20-year time series. Lower (**a–c**) sampling frequency, (**d–f**) sampling duration, and (**g–i**) a combination of lower frequency and duration tended to increase the differences in trend values between the simulated monitoring schemes and those from a subset of 255 annual time series with at least 20 years of data. Trend differences are shown using density plots of the average, across-site absolute difference for each simulation (shaded polygons) and the average of these differences across simulations (filled circles). Note that all y-axes are on the log-scale and that the range of this scale varies across panels. Source Data are provided as a Source Data file.

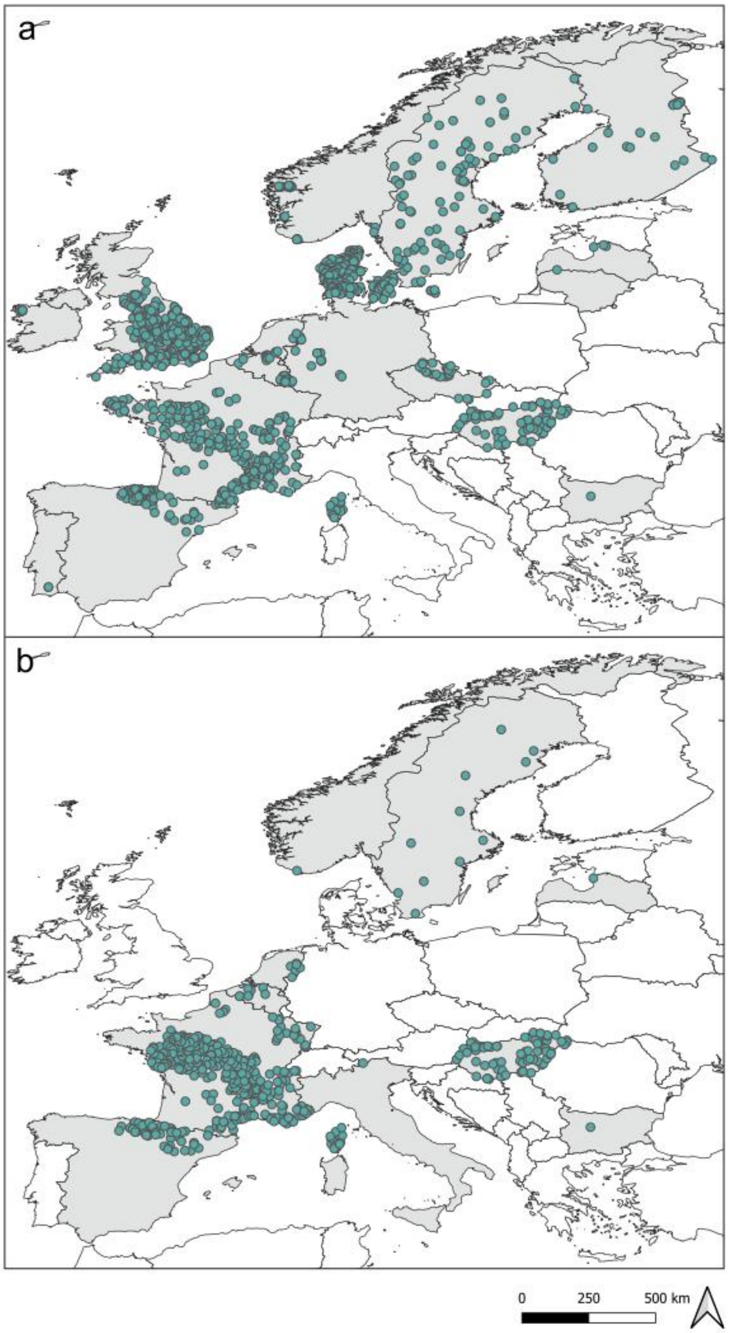

Supplementary Figure 6: Sampling sites. Geographic distribution of (**a**) the 1,353 ERITS sites with annual time series used to run simulations to test the influence of temporal resolution; and (**b**) the 485 paired ERITS and EEA sites used in the comparison between datasets differing in their temporal resolution. Countries with data are shaded in gray.

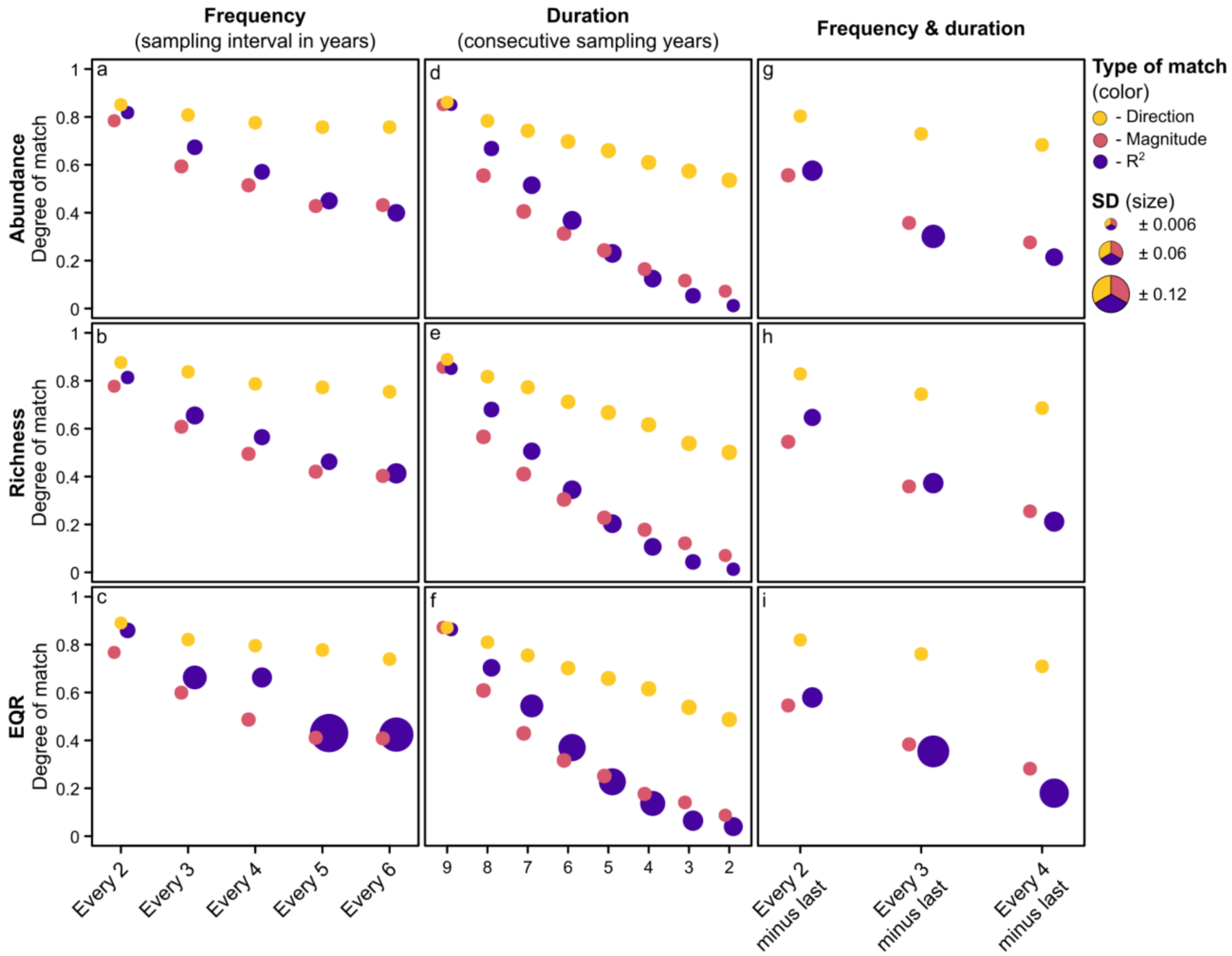

Supplementary Figure 7: Influence of lower sampling frequency and duration on site-level trends for time series with mixed taxonomic resolution data. Lower (**a–c**) sampling frequency, (**d–f**) sampling duration, and (**g–i**) a combination of lower frequency and duration led to increased trend error rates, based on declines in the degree of match between site-level trends from the simulated monitoring schemes and those from a subset of 592 annual time series with finer taxonomic resolution (a combination of family-, genus-, and species-level identifications). The degree of match is shown as the mean and standard deviation (SD; shape size) of trend direction matches (yellow circles), trend magnitude matches (pink circles), and R^2 of the trend relationships (blue circles; based on generalized linear mixed models). Source data are provided as a Source Data file.

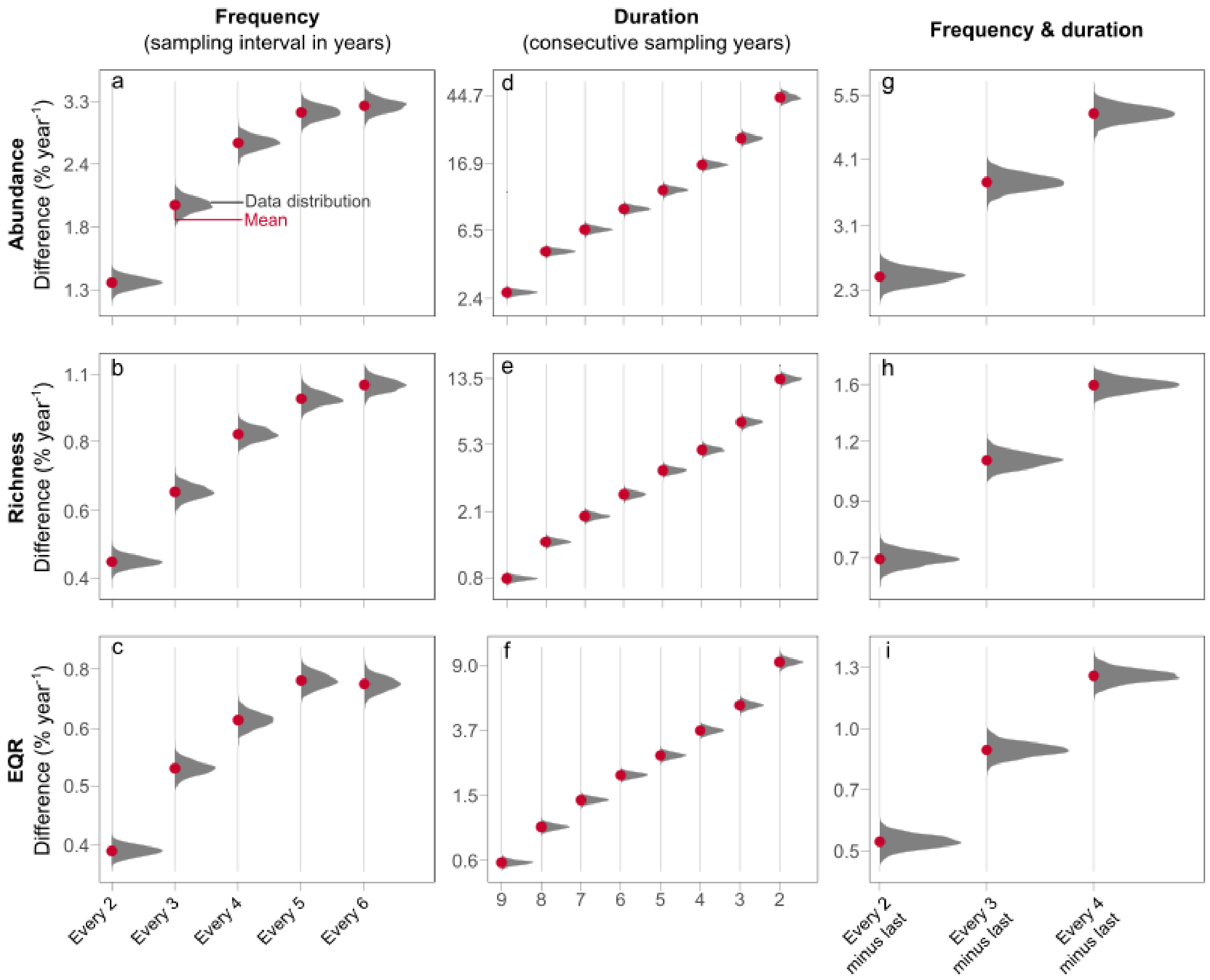

Supplementary Figure 8: Influence of lower sampling frequency and duration on differences in biodiversity trends for time series with mixed taxonomic resolution data. Lower (**a–c**) sampling frequency, (**d–f**) sampling duration, and (**g–i**) a combination of lower frequency and duration tended to increase the differences in trend values between the simulated monitoring schemes and those from a subset of 592 annual time series with finer taxonomic resolution (a combination of family-, genus-, and species-level identifications). Trend differences are shown using density plots of the average, across-site absolute difference for each simulation (shaded polygons) and the average of these differences across simulations (filled circles). Note that all y-axes are on the log-scale and that the range of this scale varies across panels. Source data are provided as a Source Data file.

Supplementary references

1. Gabriels, W., Lock, K., De Pauw, N. & Goethals, P. L. M. Multimetric Macroinvertebrate Index Flanders (MMIF) for biological assessment of rivers and lakes in Flanders (Belgium). *Limnologica - Ecology and Management of Inland Waters* **40**, 199–207 (2010).
2. Cheshmedjiev, S. & Varadinova, E. Bottom macroinvertabrates. in *Biological analysis and ecological assessment of surface water types in Bulgaria* (eds. Belkinova, D. et al.) 147–162 (P. Hilendarski University Publishing House, 2013).
3. Opatřilová, L., Němejcová, D., Zahrádková, S. & Kokeš, J. Assessment method for ecological status of rivers based on biological quality element benthic invertebrates in the Czech Republic. in 3 (2015).
4. Mičaník, T. *et al.* Hodnocení stavu útvarů povrchových vod v České republice pro 3. plánovací období plánů povodí. *Vodohospodářské technicko-ekonomické informace* **62**, 4–18 (2020).
5. Skriver, J., Friberg, N., & J. Kirkegaard. Biological assessment of running waters in Denmark: introduction of the Danish Stream Fauna Index (DSFI). *SIL Proceedings, 1922-2010*, **27**(4), 1822–1830 (2000) doi.org/10.1080/03680770.1998.11901556
6. Andersen, J. H. *et al.* Approaches for integrated assessment of ecological and eutrophication status of surface waters in Nordic Countries. *Ambio* **45**, 681–691 (2016).
7. Aroviita, J., Koskenniemi, E., Kotanen, J. & Hämäläinen, H. A priori typology-based prediction of benthic macroinvertebrate fauna for ecological classification of rivers. *Environmental Management*, *42*, 894–906 (2008).
8. Aroviita, J., Mitikka, S. & Vienonen, S. Status classification and assessment criteria of surface waters in the third river basin management cycle. *Suomen ympäristökeskuksen raportteja*, No. 37/2019 (2019).
9. Mondy, C. P., Villeneuve, B., Archaimbault, V. & Usseglio-Polatera, P. A new macroinvertebrate-based multimetric index (I2M2) to evaluate ecological quality of French wadeable streams fulfilling the WFD demands: A taxonomical and trait approach. *Ecological Indicators* **18**, 452–467 (2012).
10. Berger, E. *et al.* Water quality variables and pollution sources shaping stream macroinvertebrate communities. *Science of The Total Environment* **587–588**, 1–10 (2017).
11. Várbíró, G., Boda, P., Csányi, B., & J. Szekeres. Methodological Guide for the Collection and Processing of Macroscopic Aquatic Invertebrate Groups according to the WFD. 2nd River Basin Management Planning, RBMP 2, Government Decision 1127/2010 (V. 21.). Complete Version of the 2nd National River Basin Management Plan, Annex 6.1, 34 pp. (2015).
12. Donohue, I., McGarrigle, M. L. & Mills, P. Linking catchment characteristics and water chemistry with the ecological status of Irish rivers. *Water Research* **40**, 91–98 (2006).
13. Ozoliņš, D. *et al.* The impact of forest fertilization on the ecological quality of two hemiboreal streams. *Forests* **13**, 196 (2022).
14. Šidagytė-Copilas, E. & Arbačiauskas, K. A multimetric macroinvertebrate index for the assessment of the ecological status of Lithuanian rivers. *Limnologica* **97**, 126010 (2022).
15. Altenburg, W. *et al.* *Referenties en maatlatten voor natuurlijke watertypen voor de Kaderrichtlijn Water 2021-2027*. https://library.wur.nl/WebQuery/wurpubs/547103 (2018).
16. Leitão, F. *et al.* Habitat-oriented sampling of macroinvertebrates affects the determination of ecological status in temporary mediterranean river systems. *River Research and Applications* **30**, 1233–1247 (2014).

17. Alba-Tercedor, J. et al. Caracterización del estado ecológico de ríos mediterráneos ibéricos mediante el índice IBMWP (antes BMWP'). Limnetica, 21(3-4): 175-185 (2002).
18. Gartzia de Bikuña; B & López, E. & Leonardo, J. & Arrate, J. A. & Martínez, A. & Manzanos, A. Development of a multimetric benthic macroinvertebrate index for assessing the ecological condition of Basque streams (north of Spain). Fundamental and Applied Limnology / Archiv für Hydrobiologie. 187. 10.1127/fa1/2015/0741. (2015).
19. Munné, A. & Prat, N. Use of macroinvertebrate-based multimetric indices for water quality evaluation in Spanish Mediterranean rivers: an intercalibration approach with the IBMWP index. Hydrobiologia 628, 203–225 (2009).
20. Bighiu, M. A., Höss, S., Traunspurger, W., Kahlert, M. & Goedkoop, W. Limited effects of pesticides on stream macroinvertebrates, biofilm nematodes, and algae in intensive agricultural landscapes in Sweden. *Water Research* **174**, 115640 (2020).
21. Dahl, J. & Johnson, R. K. A multimetric macroinvertebrate index for detecting organic pollution of streams in southern Sweden. *Archiv für Hydrobiologie* **160**, 487–513 (2004) doi:10.1127/0003-9136/2004/0160-0487.
22. Paisley, M. F., Trigg, D. J. & Walley, W. J. Revision of the Biological Monitoring Working Party (BMWP) score system: derivation of present-only and abundance-related scores from field data. *River Research and Applications* **30**, 887–904 (2014).
23. Murray-Bligh, J., & M. Griffiths. Freshwater Biology and Ecology Handbook. Foundation for Water Research & Freshwater Biological Association. (2022). Available from: https://fwrinformationcentre.co.uk/biology-and-ecology-handbook/
