# Supplementary material for: Robust estimates of biodiversity change require high-resolution time series
Source: Nat Commun. 2026 Jul 7;17:5941. doi: 10.1038/s41467-026-75321-0 (PMC13342590; doi:10.1038/s41467-026-75321-0)
Supplement: Supplementary file 2 — Reporting Summary [file 41467_2026_75321_MOESM2_ESM.pdf]

nature portfolio

Corresponding author(s): Daniela Cortés-Guzmán, James S. Sinclair

Last updated by author(s): Mar 12, 2026

Reporting Summary

Nature Portfolio wishes to improve the reproducibility of the work that we publish. This form provides structure for consistency and transparency in reporting. For further information on Nature Portfolio policies, see our Editorial Policies and the Editorial Policy Checklist.

Statistics

For all statistical analyses, confirm that the following items are present in the figure legend, table legend, main text, or Methods section.

n/a	Confirmed	
☐	☒	The exact sample size (*n*) for each experimental group/condition, given as a discrete number and unit of measurement
☐	☒	A statement on whether measurements were taken from distinct samples or whether the same sample was measured repeatedly
☐	☒	The statistical test(s) used AND whether they are one- or two-sided<br>*Only common tests should be described solely by name; describe more complex techniques in the Methods section.*
☐	☒	A description of all covariates tested
☐	☒	A description of any assumptions or corrections, such as tests of normality and adjustment for multiple comparisons
☐	☒	A full description of the statistical parameters including central tendency (e.g. means) or other basic estimates (e.g. regression coefficient) AND variation (e.g. standard deviation) or associated estimates of uncertainty (e.g. confidence intervals)
☐	☒	For null hypothesis testing, the test statistic (e.g. *F*, *t*, *r*) with confidence intervals, effect sizes, degrees of freedom and *P* value noted<br>*Give P values as exact values whenever suitable.*
☒	☐	For Bayesian analysis, information on the choice of priors and Markov chain Monte Carlo settings
☒	☐	For hierarchical and complex designs, identification of the appropriate level for tests and full reporting of outcomes
☒	☐	Estimates of effect sizes (e.g. Cohen's *d*, Pearson's *r*), indicating how they were calculated

Our web collection on statistics for biologists contains articles on many of the points above.

Software and code

Policy information about availability of computer code

Data collection	All data was collated for analyses using R version 4.5.1.
Data analysis	All data analyses were performed using R version 4.5.1. All code needed to repeat our analyses is publicly available from https://doi.org/10.6084/m9.figshare.28920428.

For manuscripts utilizing custom algorithms or software that are central to the research but not yet described in published literature, software must be made available to editors and reviewers. We strongly encourage code deposition in a community repository (e.g. GitHub). See the Nature Portfolio guidelines for submitting code & software for further information.

Data

Policy information about availability of data

All manuscripts must include a data availability statement. This statement should provide the following information, where applicable:

- Accession codes, unique identifiers, or web links for publicly available datasets
- A description of any restrictions on data availability
- For clinical datasets or third party data, please ensure that the statement adheres to our policy

The data used in this study are publicly available from Figshare under the accession code https://doi.org/10.6084/m9.figshare.28920428.

Research involving human participants, their data, or biological material

Policy information about studies with human participants or human data. See also policy information about sex, gender (identity/presentation), and sexual orientation and race, ethnicity and racism.

Reporting on sex and gender	NA
Reporting on race, ethnicity, or other socially relevant groupings	NA
Population characteristics	NA
Recruitment	NA
Ethics oversight	NA

Note that full information on the approval of the study protocol must also be provided in the manuscript.

Field-specific reporting

Please select the one below that is the best fit for your research. If you are not sure, read the appropriate sections before making your selection.

☐ Life sciences ☐ Behavioural & social sciences ☒ Ecological, evolutionary & environmental sciences

For a reference copy of the document with all sections, see nature.com/documents/nr-reporting-summary-flat.pdf

Ecological, evolutionary & environmental sciences study design

All studies must disclose on these points even when the disclosure is negative.

Study description	We collated annual time series data on riverine invertebrate community composition at 1,353 sites across 18 European countries.
Research sample	The time series each consisted of at least seven individual years of data spanning a total duration of at least ten years, with sampling conducted at the same sites, during the same seasons (any three consecutive months), and using the same methods within sites through time.
Sampling strategy	Sites were selected based on which invertebrate communities were being monitored in their respective countries. The data collected from these sites also needed to satisfy our data requirements listed in the "Research sample" section above.
Data collection	Invertebrates were generally collected via multi-habitat kick-net samples collected from the river bottom.
Timing and spatial scale	Sites encompassed 18 European countries spanning across northern, central, and southern Europe. The time series across sites ranged between 1992 and 2022. Many sites provided data collected almost annually, although it is still common for time series to exhibit some one-year gaps between samples.
Data exclusions	We excluded any time series that did not meet our "Research sample" requirements, and that did not provide the necessary information to quantify community abundance, richness, and ecological quality.
Reproducibility	No experiments were performed as part of this study.
Randomization	We checked for temporal autocorrelation, and controlled for spatial/methodological differences among sites. We examined temporal relationships by first checking for temporal autocorrelation among successive samples collected from the same site, but concluded that it was too infrequent to need to be controlled. We controlled for spatial/methodological similarities by grouping data by country, and by converting analyzed metrics to % change per year.
Blinding	Blinding was not relevant to this study because data acquisition required open contact among all of the ecologists and environmental managers that provided data.

Did the study involve field work? ☐ Yes ☒ No

Reporting for specific materials, systems and methods

We require information from authors about some types of materials, experimental systems and methods used in many studies. Here, indicate whether each material, system or method listed is relevant to your study. If you are not sure if a list item applies to your research, read the appropriate section before selecting a response.

Materials & experimental systems

n/a	Involved in the study
☒	☐ Antibodies
☒	☐ Eukaryotic cell lines
☒	☐ Palaeontology and archaeology
☐	☒ Animals and other organisms
☒	☐ Clinical data
☒	☐ Dual use research of concern
☒	☐ Plants

Methods

n/a	Involved in the study
☒	☐ ChIP-seq
☒	☐ Flow cytometry
☒	☐ MRI-based neuroimaging

Animals and other research organisms

Policy information about studies involving animals; ARRIVE guidelines recommended for reporting animal research, and Sex and Gender in Research

Laboratory animals	NA
Wild animals	No animals were collected for this specific study. Only data that had already been collected for other purposes in each region was used.
Reporting on sex	NA
Field-collected samples	No samples were collected or stored specifically for this study.
Ethics oversight	No ethical approval or guidance was required because no animals were collected for this study.

Note that full information on the approval of the study protocol must also be provided in the manuscript.

Plants

Seed stocks	NA
Novel plant genotypes	NA
Authentication	NA
