# Supplementary material for: Robust estimates of biodiversity change require high-resolution time series
Source: Nat Commun. 2026 Jul 7;17:5941. doi: 10.1038/s41467-026-75321-0 (PMC13342590; doi:10.1038/s41467-026-75321-0)
Supplement: Supplementary file 3 — Transparent Peer Review file [file 41467_2026_75321_MOESM3_ESM.pdf]

nature portfolio

# Peer Review File

## Robust estimates of biodiversity change require high-resolution time series

Corresponding Author: Dr James Sinclair

**This file contains all reviewer reports in order by version, followed by all author rebuttals in order by version.**

**Attachments originally included by the reviewers as part of their assessment can be found at the end of this file.**

Version 0:

Reviewer comments:

Reviewer #1

(Remarks to the Author)
See attached comments

Reviewer #2

(Remarks to the Author)
Cortés-Guzmán et al. evaluate the effects of variability in time series duration and sample frequency on biodiversity metrics using large aquatic invertebrate datasets. The main result of their analyses is that trends of estimated measures of biodiversity (abundance, richness, EQR) do not always agree when original time series were degraded by removing samples from those time series. They evaluate these changes using slope direction, magnitude, and R2 values, all taken from generalized linear mixed models. In their first analysis, time series are degraded using a simulation approach to repeatedly truncate time series durations or remove a subset of samples. In their second analysis, similar (invertebrate time series collected in proximity to each other) but separately collected (ERITS vs. EEA) time series are compared based on their differences in sample coverage. Based on their findings that time series with lower sampling frequencies and shorter durations (whether simulated or real) show different patterns, the authors advocate for the importance of annual sampling and assert that these findings can improve conservation practice.

The significance of this work that is highlighted by the authors is in the potential to affect conservation decisions and sampling strategies. For example, they discuss the Water Framework Directive and its recommendation for sampling every 3 – 6 years. The results of the study suggest that sampling at this frequency may yield a different biodiversity trend than if sampling was conducted more frequently. Whereas the manner in which this conclusion is reached is interesting (e.g., using real data), it is not a particularly novel insight in and of itself. Researchers have been making these arguments for decades, with considerable discussion on the value and challenges of maintaining long-term ecological research and monitoring (e.g., Likens, 1989; Lindenmayer et al., 2012, and references therein). Putting that aside, the authors may also wish to consider the literature on sampling theory, as the outcome of the analyses here are intuitive with respect to sample coverage. Simply put, it is a natural conclusion that better sampling coverage over the duration of interest will more faithfully reflect the ecological reality. Though this reduces the “novelty” and “sensationalism” often required by journals like Nat Comms, it might serve the authors to acknowledge the long history of these topics.

Likens GE (ed) (1989) Long-term studies in ecology: approaches and alternatives. Springer, New York
Lindenmayer DB, Likens GE, Andersen A et al (2012) Value of long-term ecological studies. Austral Ecol 37:745–757

The data and analyses in the manuscript do support some of the claims made by the authors but I am not yet convinced on all conclusions. The first set of analyses (comparing raw and degraded time series data) is compelling. The authors clearly show that reducing sample frequency and duration has a substantial effect on the estimated biodiversity metrics. That said, I would have enjoyed more dissection of these results by the authors. For example, only reducing frequency had a smaller effect on estimates. To me, this was good news! Being able to reliably evaluate the direction of a metric is useful and, from an applied perspective, could mean that certain variables or systems need to be evaluated less frequently. In a world where conservation scientists must often perform triage, that could be a helpful, practical insight (a case-by-case scenario).

Similarly, the pattern for Duration has an interesting SD pattern (Figure 2) and the longer time series in the supplement show more complicated degradation relationships. These are the aspects that would set this work apart for me, but their discussion is absent.

I struggled somewhat with the ERITS across-site biodiversity estimates. It is not clear how the data were compiled to allow for this analysis (e.g., at what taxonomic level was biodiversity evaluated? Where there issues in harmonizing across countries? Were data summed by year? Or averaged?). It also becomes worrisome when considering the breadth in data sources. Perhaps this is covered in the papers where the data were originally published but they need some coverage in this manuscript so that it can stand on its own. The section describing these results (lines 118 – 133) was one of the most challenging of the manuscript for me. The text gives multiple sequences of percentages with a mix of raw output values and "overestimates." Many of these differences are also very small, <0.05% change, suggesting relatively good fidelity.
The final analysis in the manuscript, "Comparing trends from two European datasets," is where I have the most concern. To start, I think this is a cool idea and is the most novel part of the manuscript. This section goes beyond what I would consider to be expected outcomes of sample degradation, which characterizes the first analysis. That said, I would have greater confidence in the analysis if the authors spent more time convincing the reader that these samples are comparable. For example, within a site pair, it is not clear if the samples from ERITS and EEA were actually taken over the same period of time. There is vague text about timeframes for ERITS and EEA, generally, but dataset-wide averages are not all that compelling in this case (e.g., what if the ERITS site was collected 2010 – 2017 but EEA was 2017 – 2023? Can you rule this out?). Likewise, the authors compare sites within 1km of each other, with a mean distance of 3.1m. Admittedly, my work is mostly with marine invertebrates, but such ecosystems are characterized by patchiness across the landscape, including at the meter scale. So, can the authors convince the reader that riverine habitats are not patchy? A mean of 3.1m separation seems solid, but the described limit of 1km suggests at least some sites could have been rather far apart. There is some discussion of this in the methods section but without the code or the model outputs, it is not possible to evaluate further.
Assuming the data are comparable (which I am willing to believe, I would just like to see the authors explain this better), my single largest criticism of the manuscript is in the comparison of EQR between the EEA and ERITS datasets. The results shown in Figure 4 show that EQR trends for both datasets are indistinguishable from zero, with a slightly negative estimate for EEA and slightly positive for ERITS. This difference is interpreted as a meaningful result showing the pitfalls of worse sample coverage. I would strongly encourage the authors to revisit this interpretation. In the absence of an actual statistical test, the values of 0.003 and -0.010 appear similar. Considering also that both have confidence intervals that substantially overlap zero and each other, I would expect a statistical test to show that these are not meaningfully different from each other, or zero. The authors go on to discuss this result as meaningful to their overall conclusion, which is concerning (unless I missed something in this analysis).

Overall, the methods section and provided data would allow for approximate but not complete reproduction of the results. Most methodological choices are described but only vaguely. For example, 18 EQR methods are mentioned (in the supplement) but not the specifics of their equations, which may be meaningful given variable treatment and weighting of abundance, richness, etc. in those indices. The R code is conspicuously absent, which limits the transparency of the methods. Similarly, the authors have not provided model fits or equations for the various tests they describe in the methods section. These should be included, minimally in the supplement.

A few other comments:
- The authors appear to have written this for a general audience, which is appreciated. That said, the text reads as somewhat "arm wavy" in a few places. For example, lines 58 – 63 talk about the conservation implications, claiming new insights and sweeping implications but do little to support this grandiose language with evidence or specifics. This is something that carried on throughout the manuscript when discussing conservation implications: sweeping, broad statements about new insights into sampling strategy, with little consideration for on the ground consequences. I suggest either toning these statements down or adding specifics to show concrete implementation. Otherwise, this is a classic case of academics dictating actions to practitioners (e.g., see literature on the knowing-doing gap, research-implementation gap, etc.).

- Interpolation of missing values with the mean of the neighboring values: This is artificially going to increase the apparent quality of the "raw" data. Biological trend data always have noise, which will reduce the goodness of fit for a line and increase the variation around the slope. Interpolation likely decreases the variability inherent to the data in a time series. Reducing sample frequency and time series duration increases variability (such as in the simulations). This may not overturn the overall outcome of the analysis, but it is worth noting, especially given that the authors are claiming this analysis is grounds for influencing continent-wide monitoring protocols. My hunch is that the results would be in stronger agreement (perhaps only moderately) with non-interpolated data (i.e., take the data as it is, no artificial corrections) or by choosing a random value to fill gaps (e.g., from the range for the time series or within the bounds of neighboring points). The main text does not inform a reader that the time series being used as the basis of comparison (vs. simulated data) has been modified and this is somewhat misleading.

- Alternative approaches to manage low data coverage: The authors focus on a single recommendation for how to address the data degradation effects, which is to increase sampling frequency. I, and I imagine just about every other researcher, would be thrilled to see this become a reality but it is an unlikely outcome (broadly, at least). So, what other strategies can be used? What about more advanced statistical modeling options? Relating to the comment about interpolation, as well, the authors could consider approaches that account for missing samples and zeros in the data:
Blasco-Moreno, A., M. Pérez-Casany, P. Puig, M. Morante, and E. Castells. 2019. What does a zero mean? Understanding false, random and structural zeros in ecology. Methods in Ecology and Evolution 10:949–959.
Martin, T. G., B. A.Wintle, J. R. Rhodes, P. M. Kuhnert, S. A. Field, S. J. Low-Choy, A. J. Tyre, and H. P. Possingham. 2005. Zero tolerance ecology: improving ecological inference by modeling the source of zero observations. Ecology Letters

8:1235–1246.

- Another area absent from the manuscript is the ever-growing literature on how to measure biodiversity (e.g., work by Dr. Anne Chao and colleagues). Abundance as a measure is fraught and tightly linked to sampling effort. To a lesser but still substantial degree, so is richness. As a function of abundance and richness, EQR metrics have the potential to amplify biases inherent to these measures (but this is hidden by the lack of transparency on what the metric formulations entail – it is not reasonable to expect readers to go hunting for 18 different formulations in the literature). The authors give a general statement that they do not think these types of biases matter, but the treatment is unconvincing. This circles back to a theme, which is a general lack of specificity on many of the topics covered in the manuscript.

- Lines 29 – 36 are vague and fail to acknowledge the many ways that people try to counteract these challenges with sampling bias. This paragraph also fails to consider that many processes happen at scales beyond several years or a decade. Ironically, paleobiologists write these exact same sentences to critique biological time scales and justify the need for an even more extensive temporal perspective (an issue that at least a few of the example studies do acknowledge, e.g., Dornelas et al. 2014 on the topic of shifting baselines).

A few comments on figures:
Figure 1 – I like the schematic to help with interpretation. Perhaps add comparable visuals for the analytical metrics: direction, magnitude, R2? Though it may vary from reader to reader, the existing version of Figure 1 includes the components I found easiest to understand (time series sampling duration and frequency).

Figure 2 – This is a nice, visually pleasing figure. In the legend, perhaps change the color of the SD circles. Because they are black, and because R2 is also black and shows the largest values, it appears as if SD only applies to R2. Changes in SD for direction and magnitude (assuming there are any) are washed out. Size of points also is not mentioned in the caption, but colors are, so it might be worth adding. In this figure, SD shows a pattern of reaching max values at intermediate Duration levels. This does not appear to have been mentioned by the authors but may be worth some interpretation. I also was surprised to see a more complicated relationship (sigmoidal?) in the longer time series included in the supplement. This also was not discussed, which seems like a missed opportunity.

Figure 3 – Again, a visually appealing figure, however, I found the data challenging to evaluate because of the differences in the scales on the x-axis. Scales go from tenths to tens, with everything in between. I understand (or assume) that the point is to show reduced agreement with sample degradation but it is hard to judge the extent to which this is meaningful when neighboring plots show one value dropped by 0.05% and another by 50%, but visually they appear identical. A log-transformation could help with this.
More minor: consider removing the interspersed y-axis labels (frequency, duration, frequency & duration). This would match figure 2 and improve the readability.

Figure 4 – Panel (a) would benefit from more explanation. Taken independently, I am not sure what I am supposed to take from this part of the figure.

Overall, I enjoyed this manuscript and look forward to seeing it published. A few of the analyses could use some little fine tuning (especially EQR trends for EEA vs ERITS) and I think the manuscript (including methods and supplement) would benefit from more concrete language, specifically in the introduction and discussion. That said, the manuscript is well written and logically structure, and the authors have processed an impressive amount of data. Critiques aside, I whole-heartedly agree with the message in the manuscript! - Jansen Smith

Reviewer #3

(Remarks to the Author)
The manuscript entitled 'Robust estimates of biodiversity change require high-resolution time series' presents an extensive collection of freshwater time series to investigate the impact of two temporal sampling biases on biodiversity measures assessment: frequency and duration. The author demonstrates the impact of such biases using extensive simulation analyses from the existing time series they collected. Additionally, they compared biodiversity trends between some of the sites from their time series with those of the EEA. The authors also make a bridge beyond their research work to connect to decision-making bodies that depend on such research work by providing guidelines and uncertainty considerations.
Overall, I found the article well-organized and well-written. It highlights very well existing temporal data biases in biodiversity surveying that can heavily impact the conclusions of time series analyses. I believe that such work provides a clear and good structure in the analysis of freshwater data, which could open perspectives for other realms.

I have first general comments or questions that focus on the general understanding and the method, given that I am not familiar with freshwater ecology:

1) This is a remark for consideration: I agree with you and with your work questioning the reliability of data. I think there are times where the authors could have been slightly more cautious in their statement because not all papers showing no trends or discrepancies use the same methodology or the data in the same way. There is complexity in statistical analyses as well as in the context where the study is done (spatio-temporal extent, for example), see Boennec et al (2024).

Boënnec, M., Dakos, V. and Devictor, V. (2024), Sources of confusion in global biodiversity trends. Oikos, 2024: e10732. https://doi.org/10.1111/oik.10732

2) Another aspect that I wished the authors had pointed out is the difference between realms. I agree that the generalization is also important, but thinking about the differences (collection, identification of species difficulties) and the lack of global studies on freshwater, this is something that could have been discussed more and thus highlighting the novelty of the extensive work done here. If possible, the authors could consider adding a few sentences in the introduction and discussion about this.

3) All main analyses are done on an extensive number of sites, but testing is only gradually done every 2 to 6 years, 9 to 2 consecutive years of sampling, but is this done only for 10-year time series. This is only mentioned in the method section (lines 480-482), which is a bit late, I think. Why choose this 10-year extent and not 20 years (even if it meant fewer sites)? The 10-year extent is one of the limitations since previous work from the literature mentions that longer time spans are needed to highlight temporal changes. This is not discussed, if I recall as well.

4) The term ‘across sites’ is extensively used for part of the analyses I wonder if changing it to regional or national, since it is country-wise, would make more sense. This will also help the authors to highlight results at two spatial scale, local (site) and national (across sites).

5) Finally, did the author also consider other biodiversity metrics such as taxa turnover? The results shown in the current manuscript could be even more dramatic for such ma etric. Another reason for this question is that for readers not familiar with freshwater realms, EQRs are very uncommon diversity measures.

I have some small suggestions that might help to understand part of the work below. Note that some are suggestions and do not require strict changes if you disagree. There is a part of subjectivity in the terminology and the interpretation we have of them; I am only highlighting the ones that stood out for me.

Abstract:

line 3: ‘poor availability’ has a very negative and strong connotation to me that goes beyond the capabilities and the limitations that could exist when researchers collect data (or if they are actually open access). I encourage the reviewers to find an alternative term or to revise the sentence to highlight more temporal biases rather than the availability.

lines 7-10: Compared to the real example, the summary of the simulation results is quite vague. I suggest having a comparable statement or giving a similar level of detail.

line 16: Why local scale management only, you also have evaluated more global biodiversity impact.

Introduction:

lines 23-25, I believe this statement could be stronger with references, even if it is linked to the previous sentence.

line 30: ‘collation’ to be changed to ‘collection’

line 42: correct the citation: ‘refs. 13 versus 26’

Results

line 70: In this result section the author mentions (1) abundance (total number of individuals). I think for a better understanding and to avoid any questions before going to the method section, it should be stated the same way as it is in the method: (1) total community abundance (number of individuals).
The same goes for the second measures: in results: (2) richness (total number of taxa) and in the method section: (2) taxon richness (number of taxa).

line 71: As mentioned above, I am not familiar with EQR, but other measures of community composition change could have been considered here. Turnover has been, for example, extensively used in that context for other taxa and realms.

There is some overlap between the results presented in the second and third paragraphs. You could potentially simplify and or combine these. This is a suggestion to improve the readability of the results.

line 130: I am not sure adding the estimate per decade in addition to the ones per year is necessary.

line 147: how spatially proximate? (Maybe this information could be summarized from the method section, for example, less than 1km)

Discussion

line 180: If considering a terminology change, I mention in the method section (across site to regional or national), it will help highlight the multi-scale aspect of this study.

line 224: I think it is good that you have acknowledged some potential limitations, consider adding the one I have mentioned as well in this review, and add them here or later in the discussion.

Method:

Generally, a couple of method parts are presented but not the results themselves (see below).

line 400: If possible, add the number of time series.

lines 406-408: Does this mixed identification resolution also impact the result? This could be added to the discussion.

lines 428-429: although this is a sensible way to do it, this could be a problem and disregard extreme changes that could happen (such as a heat wave, for example). Again, this could be discussed as a limitation.

line 460-465: Maybe I missed this or misunderstood, but I don’t see these results for the simulation.

This is just a subjective but the paragraphs ‘Influence of reduced sampling frequency’ and ‘Influence of reduced sampling’ are quite repetitive and could potentially be reorganized.

line 496-497: 1km maximum of distance does not seem big, but out of curiosity (which could be of the reader as well), what does it mean for invertebrates? Is it big for invertebrate communities, or is there some reference that could back up his choice?

line 516-517: Mentioning the interaction term, maybe you should conclude that you did not keep it in the final model? Was this also tested for the method part on the simulation data (or for consistency, either stated for both or not at all, maybe)?

Version 1:

Reviewer comments:

Reviewer #1

(Remarks to the Author)
I appreciated the additional context the authors now provide in the introduction regarding the theoretical background of the work.

Thanks for examining the sensitivity of the time series lengths used and providing better linkage between the main article and the supplementary material.

I also read the responses to other reviewers and appreciate the overall care the authors provided at this point in the review stage. I'm happy with the manuscript at this point and believe it is an important contribution to the field.

(Remarks on code availability)

Reviewer #3

(Remarks to the Author)
First of all, I would like to thank the authors for considering and answering my comments and questions. I think you have provided an extensive work to reflect and modify reviewers’ comments in addition to extra analyses presented in the supplementary material. I also thank the author for providing their code, which of up most importance in our research work! I do have some comments and suggestions regarding this which I detail in the corresponding section.
I would below only highlight remaining comments I have on your work.

Remarks regarding the answers:
1) In one of your responses to my comments (point 3 of the general comments) regarding the time scaled displayed you said ’we opted to include the 10-year time series as it provided a more robust and broader-scale dataset’. I am not sure what you mean here, the 20 years would be more robust since you would have a longer time span or is it that the sampling during the 20 years is not enough spatially representative to be robust? i.e. less sites had longer time period. What I am going for here is that you could add a justification for the 10 years as being a trade-off between temporal and spatial dimensions if I understood your point correctly.
Note that I also read the response you made to Reviewer 1 which also is a good justification and information I don’t necessarily have or other readers may not have as a none specialist.

I do really appreciate the extensive discussion on the potential caveats!

Small suggestions:
Introduction:
line31-33: ‘One common limitation is low temporal resolution. This limitation typically results from opportunistic data collection, or monitoring constraints of funding, time, and personnel’. -> These sentences seem to cut the flow of the paragraph due to the repetition. A subjective change (not compulsory) would be / or something similar: ‘A common limitation is low temporal resolution which can raise from opportunistic data collection, or monitoring constraints of funding, time, and personnel.’

line 34-35: I found this statement somehow misleading: ‘such as just two or three sampling years per site7,8,10,14’. In particular, I think this does not reflect necessarily the truth from the papers cited there. If checking Dornelas paper, in the supplementary the table summarizing the time series data, most of them appear to have quite many years of sampling. Thus, I would suggest removing this highlighted part about 2 or 3 sampling years per site or to make it not as generalisable as what is currently written and using these references.
Results:
Paragraph starting line 177: This paragraph is a mix of results and discussion aspects. I suggest move certain sentences that are not descriptive to the discussion (For example lines 177-179 and lines 187-189.

Figure 4, panel a: I was wondering when highlight in yellow direction matches and in red magnitude matches, how would be represented a match in both direction and magnitude? is it just separate points? I might be missing the point so please correct me or disregard if so. Otherwise the magnitude could be the point outside colour (although this would probably not be very easy to read as well.
Lastly the legend does not indicate what is the trend line. This is only element on that figure that is not explained in the legend so for consistency this would be a good addition.

Discussion:
Line 287: Is the first time in the discussion that your study model appears river invertebrates (and also across Europe) even if you mention river sites in paragraph 3. I think a mention should come earlier in the discussion. Especially because already in paragraph 2 you comment on the existing result with numbers.

Code:
Overall, I think these codes are not user friendly and could benefits from a new organization (within the script), the actual csv files and a readme file to give the details, information that are on the excels file, version of R/Rstudio, packages and their version used) etc..

(Remarks on code availability)
Overall, I think these codes are not user friendly and could benefits from a new organization (within the script), the actual csv files and a readme file to give the details, information that are on the excels file, version of R/Rstudio, packages and their version used) etc..
Simulation code:
- the first csv file does not exist since the site file you provided is a xlsx document (multiple sheets). I understand that it is easy for everyone to correct this but for simplicity of running the analyses, it would be beneficial for the reader.
- I have to say that a script of 3700+ lines is very long to check and to follow. Please consider dividing per topics and providing smaller simulation sets as an example.
- I run into an error line 2462, running this r.squaredGLMM(mod)
Error in model.matrix(object)[, ok, drop = FALSE] :
(subscript) logical subscript too long
It might be working for you or be a version incompatibility of packages or else but please revise if needed the scripts and precise the version of R and packages that you are using.
- I ran into another error later on but could no longer keep track since my computer crashed when running this big script (even if I run it for a smaller sample of sites). Again, knowing if this analysis was run all together or not, making separate files and or indicating if remote desktop or super computer or else was used would be useful.

ERITS vs EEA code:
- Same comment regarding the file extension

**Open Access** This Peer Review File is licensed under a Creative Commons Attribution 4.0 International License, which permits use, sharing, adaptation, distribution and reproduction in any medium or format, as long as you give appropriate credit to the original author(s) and the source, provide a link to the Creative Commons license, and indicate if changes were made.
In cases where reviewers are anonymous, credit should be given to 'Anonymous Referee' and the source.
The images or other third party material in this Peer Review File are included in the article's Creative Commons license, unless indicated otherwise in a credit line to the material. If material is not included in the article's Creative Commons license and your intended use is not permitted by statutory regulation or exceeds the permitted use, you will need to obtain permission directly from the copyright holder.
To view a copy of this license, visit https://creativecommons.org/licenses/by/4.0/

**Reviewer Comments and Responses**

**Manuscript ID:** NCOMMS-25-39065

**MS title:** Robust estimates of biodiversity change require high-resolution time series

Bold comments are the reviewer comments with line numbers from the previous version of the manuscript (termed ‘Original’). Author comments are in non-bold text, with line numbers from the revised version of the manuscript (termed ‘Current’). Comments in italics are the relevant sections directly quoted from the manuscript text. We also highlighted all major edits in yellow both here and in the main text to aid with the review process.

**Reviewer 1**

**Reviewer comments for “Robust estimates of biodiversity change require high-resolution timeseries”**

**<u>Summary</u>**
**Cortés-Guzmán, Sinclair, and others use a large riverine time series biodiversity database to understand the impact of temporal data resolution on biodiversity trends. The authors found that less temporal resolution (both duration and frequency of sampling) often led to incorrect conclusions on trends, but could often still be used to detect changes in biodiversity direction. Higher resolution time series were needed to detection changes in both direction and magnitude. The authors also include a nice comparison of two time series and provide specific ideas for how sampling may affect trend assessments and management. I have a few suggested comments below, but in general, I think this is an excellent paper.**

**<u>Major comments</u>**

**I like the figure 1 conceptual explanation of the work**

**I appreciated the open data and open code associated with the project**

**I think the authors do a good job of setting up the implications of the work and applied examples related to time series bias in the introduction. However, I believe a more detailed treatment of the theoretical work on the topic (mostly around paragraphs 2+3) would be helpful. As the reviewers note, there is established literature on the topic, but it is broader and includes both theory and work with other biodivevrsity databases. Here are just a few examples, but I am biased towards some of the work more closely connected to my own. I’m not suggested the authors need to add all, or any, of these references, but I think discussing past work in the introduction, or discussion, more connected to the theoretical treatment of the topic might be helpful.**

- **Chades, I., T. G. Martin, S. Nicol, M. A. Burgman, H. P. Possingham, and Y. M.**

**Buckley. 2011. "General Rules for Managing and Surveying Networks of Pests, Diseases, and Endangered Species." Proceedings of the National Academy of Sciences 108 (20): 8323–28. https://doi.org/10.1073/pnas.1016846108.**

- **White, Easton R. 2019. "Minimum Time Required to Detect Population Trends: The Need for Long-Term Monitoring Programs." BioScience 69 (1): 40–46. https://doi.org/10.1093/biosci/biy144.**
- **Bennett, Joseph R., Sean L. Maxwell, Amanda E. Martin, Iadine Chadès, Lenore Fahrig, and Benjamin Gilbert. 2018. "When to Monitor and When to Act: Value of Information Theory for Multiple Management Units and Limited Budgets." Journal of Applied Ecology 55 (5): 2102–13. https://doi.org/10.1111/1365-2664.13132.**
- **Bennett, Joseph R., Kathleen M. Rühland, and John P. Smol. 2016. "No Magic Number: Determining Cost-Effective Sample Size and Enumeration Effort for Diatom-Based Environmental Assessment Analyses." Canadian Journal of Fisheries and Aquatic Sciences 74 (2): 208–15. https://doi.org/10.1139/cjfas-2016-0066.**
- **Bennett, Joseph R., Danielle R. Sisson, John P. Smol, Brian F. Cumming, Hugh P. Possingham, and Yvonne M. Buckley. 2014. "Optimizing Taxonomic Resolution and Sampling Effort to Design Cost-Effective Ecological Models for Environmental Assessment." Journal of Applied Ecology 51 (6): 1722–32. https://doi.org/10.1111/1365-2664.12312.**
- **Brashares, Justin S, and Moses K Sam. 2005. "How Much Is Enough? Estimating the Minimum Sampling Required for Effective Monitoring of African Reserves." Biodiversity and Conservation 14: 2709–22. https://doi.org/10.1007/s10531-005-8404-z.**
- **Field, Scott A, Patrick J O Connor, Andrew J Tyre, and Hugh P Possingham. 2007. "Making Monitoring Meaningful." Austral Ecology 32: 485–91. https://doi.org/10.1111/j.1442-9993.2007.01715.x.**
- **Legg, Colin J, and Laszlo Nagy. 2006. "Why Most Conservation Monitoring Is, but Need Not Be, a Waste of Time." Journal of Environmental Management 78: 194–99. https://doi.org/10.1016/j.jenvman.2005.04.016.**
- **Seavy, Nathaniel E., and Michelle H. Reynolds. 2007. "Is Statistical Power to Detect Trends a Good Assessment of Population Monitoring?" Biological Conservation 140 (1–2): 187–91. https://doi.org/10.1016/j.biocon.2007.08.007.**
- **Tulloch, Ayesha I. T., Iadine Chadès, and Hugh P. Possingham. 2013. "Accounting for Complementarity to Maximize Monitoring Power for Species Management: Complementary Monitoring Indicators." Conservation Biology 27 (5): 988–99. https://doi.org/10.1111/cobi.12092.**

We agree with your suggestion and have expanded on the topics we discuss in the second and third paragraphs of the Introduction (Current Lines 29–48 and 49–55), which required the addition of about 260 words and 17 references. These changes now better "set the stage" for readers in terms of the background literature regarding what we know of how temporal resolution may affect biodiversity trends.

Original Lines 29–36: "*Biodiversity data often suffer from a variety of limitations, with one common limitation being a low temporal resolution. This limitation can result from opportunistic data collation or logistical monitoring constraints. Consequently, many broad-scale biodiversity*

*studies quantify trends using data with a low sampling frequency or duration, such as just two or three sampling years per site[6,7,9,13]. Given that communities exhibit natural interannual variability, using such sparse time series can produce the appearance of a trend where there is none or vice versa[22,23] (Fig. 1). These biases could lead to incorrect conclusions and misguided management recommendations.*"

Edited to:

Current Lines 29–48: "*Biodiversity data often suffer from a variety of limitations that could lead to disagreement and unreliability in trend estimates, including spatial biases and heterogeneity in data characteristics[16,23]. One common limitation is low temporal resolution. This limitation typically results from opportunistic data collection, or monitoring constraints of funding, time, and personnel. Consequently, many broad-scale biodiversity studies quantify trends using data with a low sampling frequency or duration, such as just two or three sampling years per site[7,8,10,14]. The common use of such sparse time series could bias biodiversity trends in two principal ways. First, temporal trends quantified from a low number of samples have low statistical power, defined in this context as a low probability of detecting the correct trend value[24,25]. Second, because communities exhibit natural interannual variability, using low-resolution time series can produce trends that reflect stochastic fluctuations rather than real change[19,26] (Fig. 1a, b). These two sources of trend bias have important implications because they may accumulate when trends are aggregated across multiple sites (Fig. 1c, d), inflating error rates and leading to incorrect conclusions about biodiversity change that waste limited conservation resources and misinform management recommendations[20,27,28]. Various strategies have been developed to counteract these challenges, including statistical methods to address uncertainties and reduce biases arising from low resolution data (e.g., gap filling[29]). However, such approaches may only partly compensate for low temporal data resolution, particularly given that obtaining accurate temporal trends can require consistent monitoring for at least a decade[30,31] or even longer[28,32–35].*"

Original Lines 37–40: "*Despite these concerns, several studies have shown that generally consistent insights can still be obtained using temporally limited or irregularly sampled data. For instance, some biodiversity studies have revisited previous results to determine the influence of data limitations on estimated trends and found no substantial effect[24,25].*"

Edited to:

Current Lines 49–55: "While collecting higher resolution data would likely reduce bias in biodiversity trend estimates, this does not necessarily mean that conclusions drawn from low resolution data are incorrect. For instance, several studies show that trends quantified from lower-resolution time series can still broadly match those from higher-resolution data[36–38], and can detect general differences among sites[39] and periods[40]. Additionally, some biodiversity studies have revisited their results to determine the influence of data limitations on estimated trends and found no substantial effect[41,42]."

**Why use the shorter time series of ~10 years in the study? I think it is hard to get a sense if the trend from the 10 year time series is a fair "true trend" comparison.**

We set the requirement for our time series to span at least a decade because freshwater invertebrates are mostly short-lived (commonly <1 year life cycle), so a decade allows for several generations of change. We now clarify and reference this point for readers in the section of our Methods that describes our time series requirements (Current Lines 526–529).

However, as you highlight, a decade can still be too short to provide an accurate perspective of biodiversity change for many sites. We originally addressed this concern in a supplementary analysis of time series with 20 or more years of data, although we restricted this to our duration simulations only. We have now expanded these supplementary analyses to include the frequency and frequency/duration simulations. The results are generally the same as our main analyses, although we now note that the $R^2$ results (i.e., how well the simulations reflect variation in the complete trends) appear more robust to data gaps in longer time series. We added new text to our Results to more clearly point readers towards these additional analyses and to better outline the reasons for doing them (Current Lines 177–189), which was originally only partly described in our Methods.

Original Lines 403–404: "*...(2) at least seven individual years of data were available spanning at least a decade;*"

Edited to:

Current Lines 526–529: "*...(2) at least seven individual years of data were available spanning at least a decade, with a decade typically encompassing several generations of change in most freshwater invertebrates (which have life cycles ranging from days to years*[74]*, but a majority are less than 1 year*[75]*);*"

Current Lines 177–189 (added): "*An important caveat to the above results is that we restricted our dataset to time series spanning a decade or more (detailed in our Methods). However, even a decade can be insufficient for obtaining accurate trend estimates*[23,28,34,35]*. To test whether longer time series are more robust to data loss, we repeated our analyses for a subset of 255 sites out of 1,353 total with at least 20 years of data and expanded the duration simulations to 2–19 years in comparison to the first 20 years. We found the same overall results (Supplementary Figs. 3–5), indicating our conclusions were generally consistent whether shorter or longer time series were used. A notable exception was the lower sensitivity of $R^2$ to declines in frequency and duration. Specifically, changes in $R^2$ tended to mirror changes in the magnitude matches in our full analyses (Fig. 2); however, when limited to time series with at least 20 years of data, $R^2$ did not decline as sharply as magnitude (Supplementary Figs. 3, 4). These patterns suggest that, while trend magnitudes are still sensitive to data loss, longer time series may better preserve trend variation among sites even at lower sampling frequencies and durations.*"

**Relatedly, I'm not sure how the results starting on line 88 can make sense if there was sampling once every 6 years if there was a 10-year time series**

Depending on whether the simulation started in the first or second year, a ten-year time series sampled every six years provided data on years 1 and 7, or years 2 and 8, thus only offering two

years of data for temporal trend analyses. Such low temporal data resolution is not uncommon in biodiversity research, as mentioned in the Introduction, and we added some text to our Methods to make the amount of data clearer for readers.

Original Lines 432–433: "*We simulated different sampling frequencies by only including data from every 2–6 years for each site.*"

Edited to:

Current Lines 600–603: "*We simulated different sampling frequencies by only including data from every 2–6 years for each site (see Table 1 for a summary of analyses). Given we set the minimum time series duration to ten years, these frequencies meant that each simulation included at least two years of data to quantify trends.*"

**Early on (perhaps in the last paragraph of the introduction), I think it is important to clarify what you mean by "trend analysis" or "trends". A lot of folks use this to mean simple linear trends, which I believe is what was done here, but that doesn't have to be the case. I would just clarify that is what you are doing. In the discussion, I would then note as a limitation that you only looked at linear trends over time and how that could be the avenue of future work. There is already theoretical literature on linear vs nonlinear time series ideas related to biodiversity.**

We now clarify in the last paragraph of our Introduction that our focus is on linear biodiversity trends (Current Lines 72–74). We also added some text to our limitations paragraph to highlight the importance of considering non-linear trends (Current Lines 288–294).

Original Lines 53–54: "*We then compared how these changes altered site-level and across-site estimates of biodiversity trends.*"

Edited to:

Current Lines 72–74: "*We then compared how these changes altered site-by-site and across-site estimates of linear biodiversity trends.*"

Original Lines 225–229: "*This comparison does not mean that annual sampling is always optimal, nor are we suggesting that annual time series are the best representation of biodiversity change across taxa and ecosystems. However, annual data collected across at least a decade still provides a benchmark that is typically of a higher resolution compared to a variety of biodiversity datasets and monitoring schemes that often use lower resolution time series.*"

Edited to:

Current Lines 288–294: "*This approach uses a benchmark that is typically of a higher resolution compared to a variety of broad-scale biodiversity datasets and monitoring schemes. However, depending on the taxonomic group and ecosystem in question, biodiversity change may be better represented by non-linear dynamics (e.g., in ecosystems that experienced a regime shift*[55,56]*), annual sampling may not be necessary*[36]*, and multiple decades of monitoring may be needed to fully represent biodiversity change*[31,34,35]*.*"

**For future work (it feels like too much for this paper), it may be interesting to try and understand the why of time series robustness. For example, the authors could see if trends were more robust to temporal resolution dependent on the taxa, location, etc.**

This is a great idea and we had actually initially designed our study with these types of comparisons in mind, specifically comparing the frequency/duration results for stationary communities versus those undergoing directional change. Unfortunately, as you suggest, these additional comparisons turned out to be too much for a single study and so had to be shelved for later. We do, however, plan to explore these ideas in future work and to compare data needs across different taxonomic groups (e.g., invertebrates, fish, and macrophytes) and European regions.

**Line 202: I'd be careful about noting what your paper says versus previous literature as the current work doesn't say anything about specific management actions. I think some simple rephrasing here might be helpful**

We rephrased this section as suggested to focus more on what the biodiversity patterns suggest for overall management, rather than discussing specific management actions.

Original Lines 201–207: "*The former dataset suggests European river management efforts, which typically focus on controlling organic pollution*[32,33]*, successfully maintained biodiversity since the 2010s, although other stressors—such as micropollutants and climate change—likely need to be addressed to foment further improvements*[26]*. The latter dataset suggests that river management actions have broadly failed to prevent biodiversity loss, and additional measures are, therefore, critical for halting further declines.*"

Edited to:

Current Lines 263–268: "*For example, site-level ecological quality data reported as part of the EU Water Framework Directive (WFD) provides periodic updates on progress towards achieving a 'good' ecological status for European waterbodies. Within this framework where site-level assessments feed into regional and continental evaluations, trend discrepancies risk misdirecting local interventions and biasing broader status reports.*"

**Line 224-230: Referring to the literature I referenced above, I think this paragraph could be better grounded in the literature**

We edited this section to address this concern, and to incorporate some of the new references added in our similar edits to the Introduction.

Original Lines 224–229: "*Our study addresses how annual biodiversity trends compare to those with lower temporal resolution. This comparison does not mean that annual sampling is always optimal, nor are we suggesting that annual time series are the best representation of biodiversity change across taxa and ecosystems. However, annual data collected across at least a decade still provides a benchmark that is typically of a higher resolution compared to a variety of biodiversity datasets and monitoring schemes that often use lower resolution time series.*"

Edited to:

Current Lines 287–294: "*Our study addresses how linear trends in river invertebrate time series collected annually over at least a decade compared to those with lower temporal resolution. This approach uses a benchmark that is typically of a higher resolution compared to a variety of broad-scale biodiversity datasets and monitoring schemes. However, depending on the taxonomic group and ecosystem in question, biodiversity change may be better represented by non-linear dynamics (e.g., in ecosystems that experienced a regime shift[55,56]), annual sampling may not be necessary[36], and multiple decades of monitoring may be needed to fully represent biodiversity change[31,34,35].*"

**Line 241: You note the work provides a "rule-of-thumb", but I'm not sure what you are getting at. I think you might be trying to note that low resolution datasets can provide information on direction while higher resolution datasets are needed for other questions, but I'm not sure.**

Apologies for our inarticulate wording. We simply meant that our results can be used to guide monitoring and have re-phrased this section to clarify this point.

Original Lines 240–250: "*From a practical standpoint, our study provides a rule-of-thumb guide for planning the temporal component of global monitoring efforts (e.g., BioTime[41], GBiOS[42], or see elter-ri.eu), which must balance accurate trend estimation with realistic constraints in funding, time, and personnel. Such planning requires considering the needs and predicted limitations of a given monitoring scheme. Relying on data with a low temporal resolution allows for a broad-scale overview of trend direction, which reduces effort and cost but may come at the expense of trend value accuracy. In contrast, obtaining accurate, year-to-year estimates of the degree of biodiversity change across a decade or more, and ensuring, for example, > 70% trend value accuracy across sites, requires higher resolution information, such as gathering data annually or every two years across at least 80% of the desired time frame…*"

Edited to:

Current Lines 304–310: "*Our findings have broader practical implications for future monitoring and general implications for our ecological understanding of biodiversity change. From a practical standpoint, our study can help inform and design temporal global monitoring efforts (e.g., GBiOS[59], or see elter-ri.eu), which must balance accurate trend estimation with realistic constraints in funding, time, and personnel. For example, our results suggest that programs limited to low-resolution monitoring can still obtain an accurate, broad-scale overview of trend*

*directions, but may fail to accurately estimate the rate of biodiversity change over time.*"

**In the discussion, I would come back to the limitation of this being a particular set of taxa in a particular set of systems.**

We now remind readers in the limitations section of our Discussion that our analyses focus on river invertebrates, and that the best ways to represent biodiversity change can vary among taxonomic groups and ecosystem types (edits shown above in our response to your comment for Line 224-230).

**Minor comments**

**Figure 2: Perhaps use different shapes for the "Type of match". They were hard to distinguish in black and white**

We opted to use different colors because using shapes would require a separate legend for each SD size for each shape (e.g., small, medium, and large circles, squares, and triangles). This ends up being confusing because the SDs for direction and magnitude are generally narrow, so the larger symbol sizes for these metrics in the legend would not actually appear in the panels. Instead, to address your concerns, we improved the color contrast for the points, making them more distinct in greyscale, and added the three colors to the SD portion of the legend. The three colors can now be easily distinguished in black and white:

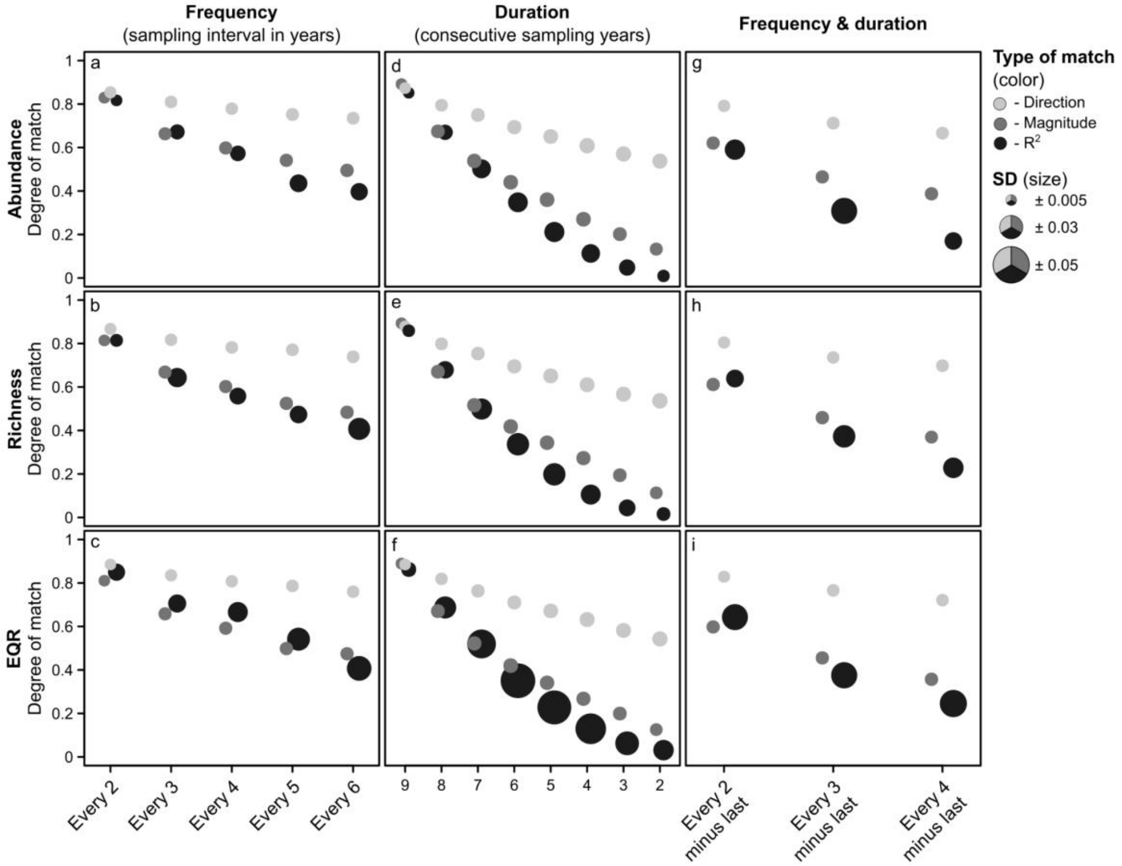

**Figure 2: I found the y-axis initially confusing. Perhaps it would be better to have "Degree of match" closer to the axis and the words Abundance, Richness, EQR to be bold and further to the left (more akin to figure 3)**

We have switched the axis labels as suggested (edits shown in the figure above).

**Figure 2: Why use size to represent SD instead of simple error bars?**

We initially tried using bars, but often the errors were so small the bars didn't extend beyond the points themselves, making it seem like these points had no error. Using point size avoids this problem.

**Reviewer 2**

**Cortés-Guzmán et al. evaluate the effects of variability in time series duration and sample frequency on biodiversity metrics using large aquatic invertebrate datasets. The main result of their analyses is that trends of estimated measures of biodiversity (abundance, richness, EQR) do not always agree when original time series were degraded by removing samples from those time series. They evaluate these changes using slope direction, magnitude, and R2 values, all taken from generalized linear mixed models. In their first analysis, time series are degraded using a simulation approach to repeatedly truncate time series durations or remove a subset of samples. In their second analysis, similar (invertebrate time series collected in proximity to each other) but separately collected (ERITS vs. EEA) time series are compared based on their differences in sample coverage. Based on their findings that time series with lower sampling frequencies and shorter durations (whether simulated or real) show different patterns, the authors advocate for the importance of annual sampling and assert that these findings can improve conservation practice.**

**The significance of this work that is highlighted by the authors is in the potential to affect conservation decisions and sampling strategies. For example, they discuss the Water Framework Directive and its recommendation for sampling every 3 – 6 years. The results of the study suggest that sampling at this frequency may yield a different biodiversity trend than if sampling was conducted more frequently. Whereas the manner in which this conclusion is reached is interesting (e.g., using real data), it is not a particularly novel insight in and of itself. Researchers have been making these arguments for decades, with considerable discussion on the value and challenges of maintaining long-term ecological research and monitoring (e.g., Likens, 1989; Lindenmayer et al., 2012, and references therein). Putting that aside, the authors may also wish to consider the literature on sampling theory, as the outcome of the analyses here are intuitive with respect to sample coverage. Simply put, it is a natural conclusion that better sampling coverage over the duration of interest will more faithfully reflect the ecological reality. Though this reduces the "novelty" and "sensationalism" often required by journals like Nat Comms, it might serve the authors to acknowledge the long history of these topics.**

**Likens GE (ed) (1989) Long-term studies in ecology: approaches and alternatives. Springer, New York**

**Lindenmayer DB, Likens GE, Andersen A et al (2012) Value of long-term ecological studies. Austral Ecol 37:745–757**

We agree that the importance of long-term studies has been recognized before. We revised the following sections of the Introduction (Current Lines 35–48) and Discussion (Current Lines 233–235) to better acknowledge this literature:

Original Lines 33–36: "*Given that communities exhibit natural interannual variability, using such sparse time series can produce the appearance of a trend where there is none or vice versa*[22,23] *(Fig. 1). These biases could lead to incorrect conclusions and misguided management*

*recommendations.*"

Edited to:

Current Lines 35–48: "*The common use of such sparse time series could bias biodiversity trends in two principal ways. First, temporal trends quantified from a low number of samples have low statistical power, defined in this context as a low probability of detecting the correct trend value*[24,25]*. Second, because communities exhibit natural interannual variability, using low-resolution time series can produce trends that reflect stochastic fluctuations rather than real change*[19,26] *(Fig. 1a, b). These two sources of trend bias have important implications because they may accumulate when trends are aggregated across multiple sites (Fig. 1c, d), inflating error rates and leading to incorrect conclusions about biodiversity change that waste limited conservation resources and misinform management recommendations*[20,27,28]*. Various strategies have been developed to counteract these challenges, including statistical methods to address uncertainties and reduce biases arising from low resolution data (e.g., gap filling*[29]*). However, such approaches may only partly compensate for low temporal data resolution, particularly given that obtaining accurate temporal trends can require consistent monitoring for at least a decade*[30,31] *or even longer*[28,32–35]."

Original Lines 172–175: "*Researchers, managers, and policymakers using biodiversity data and designing monitoring schemes must, therefore, ensure sufficient temporal resolution for assessing biodiversity status, making informed conservation decisions, and prioritizing effective actions.*"

Edited to:

Current Lines 233–235: "*The importance of accurate estimates, and their applications, underscores the value of high-resolution temporal data and echoes repeated calls for more long-term ecological research*[31,33]."

Furthermore, while we agree the general principle of "more data is better" is well established, part of the unique contribution of our work is to examine "how much data is enough" to obtain reliable biodiversity trends, thereby linking theoretical expectations with empirical evidence and providing practical guidance. We made some changes to our Introduction to more clearly emphasize this aspect of our work and the background literature.

Original lines (37–38): "*Despite these concerns, several studies have shown that generally consistent insights can still be obtained using temporally limited or irregularly sampled data*."

Edited to :

Current Lines 49–53: "*While collecting higher resolution data would likely reduce bias in biodiversity trend estimates, this does not necessarily mean that conclusions drawn from low resolution data are incorrect. For instance, several studies show that trends quantified from lower-resolution time series can still broadly match those from higher-resolution data*[36–38]*, and can detect general differences among sites*[39] *and periods*[40]."

Original lines (42–43): "*These points suggest that certain data limitations may be acceptable*."

Edited to:

Current Lines 57–58: "*These points suggest that certain data limitations may be acceptable without biasing biodiversity trend estimates*."

Original lines (58–60): "*Our results provide new insights into how common temporal data limitations affect biodiversity trend estimates.*"

Edited to:

Current Lines 77–79: "*Our results show how infrequent or short-duration sampling can misrepresent biodiversity trends, providing empirical evidence of the value of long-term, high-resolution data.*"

**The data and analyses in the manuscript do support some of the claims made by the authors but I am not yet convinced on all conclusions. The first set of analyses (comparing raw and degraded time series data) is compelling. The authors clearly show that reducing sample frequency and duration has a substantial effect on the estimated biodiversity metrics. That said, I would have enjoyed more dissection of these results by the authors. For example, only reducing frequency had a smaller effect on estimates. To me, this was good news! Being able to reliably evaluate the direction of a metric is useful and, from an applied perspective, could mean that certain variables or systems need to be evaluated less frequently. In a world where conservation scientists must often perform triage, that could be a helpful, practical insight (a case-by-case scenario). Similarly, the pattern for Duration has an interesting SD pattern (Figure 2) and the longer time series in the supplement show more complicated degradation relationships. These are the aspects that would set this work apart for me, but their discussion is absent.**

You raise a good point about the smaller effect of reducing frequency versus duration. We were initially hesitant to directly compare these results because the duration simulations were restricted to 10 years, whereas the frequency simulations can include longer time series (10, 15, 20+ years). This means the total amount of data per simulation is not always comparable. For example, a 10-year time series sampled every 2 years provides 5 years of data (comparable to the 5-year duration simulations), but a 20-year time series sampled every 2 years provides 10 years of data, which has no equivalent in the duration simulations. That said, you are correct that some generalizable differences remain. For example, even the lowest frequency simulations (e.g., every 6 years), which provide very few years of data, still have a higher degree of match compared to the duration simulations with a similar quantity of data. We added some text to the Discussion to outline these results for readers (Current Lines 308–317).

Regarding the SD patterns, the more complex degradation mainly arises from low variation as the matches approach 0%. SD is small for simulations with more data because they generally match the annual time series, then the SD increases at intermediate data loss as the simulations

become more variable, then SD decreases with further data loss as all simulations now have a consistently low match around 0%, resulting in little to no variance. We added some text to our Results to better explain this for readers (Current Lines 136–144).

Current Lines 136–144 (added): "*Reducing sampling frequency, duration, or both also tended to increase the* $R^2$ *standard deviation (SD), whereas the SD for direction and magnitude showed minimal variation (Fig. 2). Higher SD indicates less consistency among simulations, meaning the degree to which the simulated trends captured the same, among-site variation as the 'complete' trends (i.e., annual or 10-year) varied more among simulations compared to trend directions and magnitudes. This effect was most obvious for EQRs at the lowest sampling frequencies (e.g., every 5–6 years; Fig. 2c), and at intermediate durations (e.g., 4–6 years; Fig. 2f). Note, however, that the* $R^2$ *SDs declined at the shortest durations because these simulations consistently explained close to 0% variation.*"

Original Lines 245–251: "*Relying on data with a low temporal resolution allows for a broad-scale overview of trend direction, which reduces effort and cost but may come at the expense of trend value accuracy. In contrast, obtaining accurate, year-to-year estimates of the degree of biodiversity change across a decade or more, and ensuring, for example, > 70% trend value accuracy across sites, requires higher resolution information, such as gathering data annually or every two years across at least 80% of the desired time frame (e.g., 8 out of 10 years, Fig. 2; or 17 out of 20 years, see Supplementary Fig. 1).*"

Edited to:

Current Lines 308–317: "*For example, our results suggest that programs limited to low-resolution monitoring can still obtain an accurate, broad-scale overview of trend directions, but may fail to accurately estimate the rate of biodiversity change over time. We also found that trend estimates were less affected by declines in frequency than duration, as evidenced by higher site-by-site matches, higher explained variances, and lower differences compared to the annual trends. This result likely reflects the greater sensitivity of linear temporal trends to the initial and final years of a time series*[26,60]*. Reducing frequency still maintains coverage of both the early and late years of a time series, whereas reducing duration excludes either the earlier or later years entirely. Thus, reducing sampling frequency may be a cost-saving measure that provides higher trend accuracy compared to reducing sampling duration.*"

**I struggled somewhat with the ERITS across-site biodiversity estimates. It is not clear how the data were compiled to allow for this analysis (e.g., at what taxonomic level was biodiversity evaluated? Where there issues in harmonizing across countries? Were data summed by year? Or averaged?). It also becomes worrisome when considering the breadth in data sources. Perhaps this is covered in the papers where the data were originally published but they need some coverage in this manuscript so that it can stand on its own. The section describing these results (lines 118 – 133) was one of the most challenging of the manuscript for me. The text gives multiple sequences of percentages with a mix of raw output values and "overestimates." Many of these differences are also very small, <0.05% change, suggesting relatively good fidelity.**

We made several edits to address the concerns regarding across-site trend estimates, particularly for: a) taxonomic identification level, b) the potential need for harmonization across countries, and c) the treatment of annual data. We now clarify that the across-site estimates represent the average of all individual, site-level slopes, each expressed as % change per year (Results, Current Lines 146–151). Percent change is directly comparable across sites without the need to harmonize the taxonomic level or abundance units, and the data is not summed or averaged prior to slope estimation. We also revised our Methods to better outline these points (Current Lines 578–580).

Furthermore, we acknowledge the challenge for readers in determining the biological relevance of over- or under-estimating the across-site trends by a certain percentage. While we originally included this context in the Discussion, your comment highlighted that it is important to provide it where readers first encounter these values. We therefore added text in the Results to help contextualize the relevance of the estimation errors along with some references (Current Lines 157–162).

Original Lines 113–116: "*Lower sampling frequency and duration also led to greater error in across-site estimates of overall biodiversity change, with error represented as the average difference in the across-site trend estimate for the complete time series (or the first 10 years for duration) versus the same estimates from the simulated monitoring schemes (Fig. 3).*"

Edited to:

Current Lines 146–151: "*Lower sampling frequency and duration also led to greater error in biodiversity trend estimates, represented as the average of the absolute difference between the simulated versus complete trend estimates (i.e., % change per year in abundance, richness, or EQR). This comparison assesses, across all sites, exactly how much the simulated trends deviated from those derived from the complete time series, complementing the direction, magnitude, and $R^2$ results presented above.*"

Original Lines 123–126: "*This degree of error increased as sampling frequency decreased, with sampling every 6 years resulting in higher respective overestimates of +1.44%, +0.18%, and +0.20% year$^{-1}$.*"

Edited to:

Current Lines 157–162: "*However, the degree of error became more pronounced as sampling became less frequent, with sampling every 6 years corresponding to average differences of 3.80%, 1.10%, and 0.64% year$^{-1}$. To help contextualize these errors, overall biodiversity change across realms or taxonomic groups often falls within just 0–2% per year[10,14,43], meaning deviations of even 1% can translate to a large misestimate of the overall degree of biodiversity change.*"

Original Lines 445–447: "*All slopes were converted to percent change per year by log-transforming the metrics prior to modeling, then exponentiating the slopes and subtracting 1. This conversion ensured that slope units were comparable among sites and metrics.*"

Edited to:

Current Lines 578–580: “All slopes were converted to percent change per year by log-transforming the metrics prior to modeling, then exponentiating the slopes and subtracting 1. This conversion ensured that slopes were comparable among sites regardless of differences in abundance units, taxonomic resolution, or total richness.”

**The final analysis in the manuscript, “Comparing trends from two European datasets,” is where I have the most concern. To start, I think this is a cool idea and is the most novel part of the manuscript. This section goes beyond what I would consider to be expected outcomes of sample degradation, which characterizes the first analysis. That said, I would have greater confidence in the analysis if the authors spent more time convincing the reader that these samples are comparable. For example, within a site pair, it is not clear if the samples from ERITS and EEA were actually taken over the same period of time. There is vague text about timeframes for ERITS and EEA, generally, but dataset-wide averages are not all that compelling in this case (e.g., what if the ERITS site was collected 2010 – 2017 but EEA was 2017 – 2023? Can you rule this out?). Likewise, the authors compare sites within 1km of each other, with a mean distance of 3.1m. Admittedly, my work is mostly with marine invertebrates, but such ecosystems are characterized by patchiness across the landscape, including at the meter scale. So, can the authors convince the reader that riverine habitats are not patchy? A mean of 3.1m separation seems solid, but the described limit of 1km suggests at least some sites could have been rather far apart. There is some discussion of this in the methods section but without the code or the model outputs, it is not possible to evaluate further.**

To address your concerns, we now clarify that, for each site pair, the EEA sampling years always fell within the ERITS sampling years, supporting the validity of our comparisons (Current Lines 201–204).

Regarding the distance between sites, we apologize for the confusion caused by our poor presentation of these details. The 1 km threshold is not the maximum distance, but rather a cutoff value we used to initially identify a subset of sites in both datasets to check. The maximum distance between site pairs is actually ~115 m, which we considered close enough to be the same section of river. We added the actual range of distances between site pairs to clarify these points for readers (Current Lines 626–629). We also originally conducted a test of the influence of site distance (Original Lines 510–517), which was not significant. We made some changes to this section to make this information easier for readers to find by placing it in a separate paragraph and adjusting to clearer wording (Current Lines 645–653).

Original Lines 146–148: *"We ensured the EEA sites were spatially proximate and in the same rivers as their paired ERITS sites."*

Edited to:

Current Lines 201–204: "*We ensured that the paired ERITS and EEA sites were located in the same rivers, were spatially proximate (average distance of 3.1 m), and that the sampling years for the EEA sites always fell within the sampling years of their paired ERITS sites.*"

Original Lines 495–497: "*...that were sampled from the same rivers and were within at least 1 km of each other (average distance of 3.1 m; Supplementary Fig. 2b).*"

Edited to:

Current Lines 626–629: "*To determine the site pairs, we first used a cutoff distance threshold of 1 km to identify sites in the same geographic area. We then selected only site pairs that were the closest to one another and within the same waterbody (average distance between pairs of 3.1m; range 1.2–114.6 m; Supplementary Fig. 6b).*"

Original Lines 510–517: *"This model included the distance between site pairs as an interaction term to determine whether the relationship depended on site proximity, and a random intercept term for 'country'. From this model, we extracted the marginal $R^2$ to assess how well the slopes from the ERITS sites (i.e., the reference sites) predicted those from the EEA sites. We also tested the significance ($P < 0.05$) of the interaction with distance by dropping the interaction from the model and comparing the model with versus without the interaction using a likelihood ratio test. The interaction term was not significant ($P = 0.10$)."*

Edited to:

Current Lines 645–653: "*Lastly, we tested the influence of distance on the modeled relationship between the EEA and ERITS trends, given the site pairs exhibited a range of distances between one another which could affect the relationship between their slopes. To test this, we included an interaction term for distance (m) between site pairs in the above GLMM. We then determined the significance ($P < 0.05$) of the interaction and the individual distance term by comparing models with and without these terms using Likelihood Ratio (LR) tests. The interaction was marginally non-significant (LR test, $L = 3.73$, $df = 1$, $P = 0.053$), and the individual distance term was also non-significant (LR test, $L = 1.01$, $df = 1$, $P = 0.31$), indicating that controlling for the distance between site pairs would not substantially affect our results, so we excluded the distance term from the final model.*"

**Assuming the data are comparable (which I am willing to believe, I would just like to see the authors explain this better), my single largest criticism of the manuscript is in the comparison of EQR between the EEA and ERITS datasets. The results shown in Figure 4 show that EQR trends for both datasets are indistinguishable from zero, with a slightly negative estimate for EEA and slightly positive for ERITS. This difference is interpreted as**

**a meaningful result showing the pitfalls of worse sample coverage. I would strongly encourage the authors to revisit this interpretation. In the absence of an actual statistical test, the values of 0.003 and -0.010 appear similar. Considering also that both have confidence intervals that substantially overlap zero and each other, I would expect a statistical test to show that these are not meaningfully different from each other, or zero. The authors go on to discuss this result as meaningful to their overall conclusion, which is concerning (unless I missed something in this analysis).**

We thought the overall trend differences would be the simplest for readers to grasp, but as you point out they are not that different so our focus on this point was a mistake. What we should have focused on are the mismatches in the individual, site-level trends and their implications for local management. To do so, we now show the absolute differences in the trend estimates between site pairs in Fig. 4b, which mirrors a similar change we made to our simulation analyses to address a comment from another reviewer. Showing the absolute differences better highlights the degree to which the ERITS vs EEA trends differ from one another. The average, across-site trend ends up similar between datasets because, although the site-level trends differ, these differences are somewhat equal in both positive and negative directions, resulting in similar averages. We also edited the ERITS vs EEA Results (Current Lines 211–222) and Discussion (Current Lines 257–268) to acknowledge the similarity in the overall estimates while focusing more on the individual, site-level differences, which could lead to opposing recommendations for local management.

New Fig. 4 and caption:

***Figure 4. Comparison of estimated EQR trends between two datasets that differ in temporal resolution.*** *Differences in temporal resolution between the EEA and the ERITS sites influenced: (**a**) matches between the direction and magnitude of site-level ecological quality trends (EQR change year$^{-1}$), and the $R^2$ of their relationship (based on a GLMM); (**b**) trend estimates, represented as the absolute differences between the EEA and ERITS slope values; and (**c, d**) across-site estimates of ecological quality change. Density plots in (**c, d**) show the probability distribution of trend estimates for the 485 sites, dots and error bars represent the mean and standard deviation, and the upper boxes show the median and distributions for 50%, 75%, and 95% of the data. Red dots show data outside 50%.*

Original Lines 155–161: *"Additionally, the overall, across-site estimate of biodiversity change differed between the two datasets (Fig. 4b, c). The ERITS dataset indicated a slight improvement through time in ecological quality (average across-site trend estimate of 0.003 ± 0.024). In contrast, the EEA dataset indicated degradation (average of -0.010 ± 0.187). In addition, the ERITS estimates exhibited less variation than the EEA estimates (note the narrower scale in the x-axes and the smaller size of the interquartile ranges in Fig. 4b versus c)."*

Edited to:

Current Lines 211–222: "*Furthermore, trend estimates differed between site pairs by an average of 0.063 year$^{-1}$ (Fig. 4b). This difference translates to 0.6 over a decade, which is over half the total EQR range of 0 to 1, reflecting a substantial potential to mischaracterize the degree to*

*which a site has degraded or recovered compared to reference conditions. The density distribution of the EEA trends was also noticeably wider compared to the ERITS trends (note the narrower scale in the x-axes and the smaller size of the interquartile ranges in Fig. 4c versus d), indicating a wider range and greater variation in trend values in the EEA dataset. Additionally, while the average, across-site estimate of biodiversity change was similar between datasets (0.0030 ± 0.024% year$^{-1}$ for ERITS versus -0.010 ± 0.19% year$^{-1}$ for EEA), their signs differed, with the positive average trend in the ERITS dataset indicating slight temporal improvement in ecological quality, whereas the negative average trend in the EEA dataset indicated slight degradation.*"

Original Lines 196–207: "*Moreover, the lower temporal resolution of the EEA sites resulted in higher variability and opposite estimates of overall biodiversity change compared to the ERITS dataset. These differences led to the ERITS dataset indicating a slight overall improvement in the invertebrate-based ecological quality of European rivers from 2010 to 2023, whereas the EEA dataset indicated overall degradation. Such contrasting perspectives of biodiversity change translate to substantially different management recommendations. The former dataset suggests European river management efforts, which typically focus on controlling organic pollution[32,33], successfully maintained biodiversity since the 2010s, although other stressors—such as micropollutants and climate change—likely need to be addressed to foment further improvements[26]. The latter dataset suggests that river management actions have broadly failed to prevent biodiversity loss, and additional measures are, therefore, critical for halting further declines.*"

Edited to:

Current Lines 257–268: "*The lower temporal resolution of the EEA sites also resulted in an opposite estimate of the direction of overall change compared to the ERITS dataset, although both estimate values were similar and close to zero, likely due to high variability, particularly in the EEA trends. While differences in the overall outcomes were minimal, their contrasting directions and the generally differing perspectives of change between individual site pairs indicated the potential for data temporal resolution to influence local-scale management recommendations. For example, site-level ecological quality data reported as part of the EU Water Framework Directive (WFD) provides periodic updates on progress towards achieving a 'good' ecological status for European waterbodies. Within this framework where site-level assessments feed into regional and continental evaluations, trend discrepancies risk misdirecting local interventions and biasing broader status reports.*"

**Overall, the methods section and provided data would allow for approximate but not complete reproduction of the results. Most methodological choices are described but only vaguely. For example, 18 EQR methods are mentioned (in the supplement) but not the specifics of their equations, which may be meaningful given variable treatment and weighting of abundance, richness, etc. in those indices. The R code is conspicuously absent, which limits the transparency of the methods. Similarly, the authors have not provided model fits or equations for the various tests they describe in the methods section. These should be included, minimally in the supplement.**

Unfortunately, we cannot provide the R code used to produce the EQRs because we did not calculate them in R, but rather they were provided by our co-authors using country- and river-specific methods and software (e.g., RIVPACS in the UK; https://www.ceh.ac.uk/data/software-models/rivpacs-reference-database; or Perlodes in Germany; https://gewaesser-bewertung-berechnung.de/index.php/perlodes-online.html). Additionally, there is often no one equation that encapsulates the indices used as they are adapted to each country and river to ensure each site is being compared to the most suitable indices and reference ecosystems. Navigating this process requires specialized knowledge of the assessment system in each country, which is not feasible for an average reader to replicate without involving many different specialists, which is partly why we have so many co-authors because their aid is essential to completing the EQR calculations. We added a little extra detail to our Methods to clarify this point (Current Lines 564–566). Moreover, the EQR results were similar to those for abundance and richness, supporting the robustness of the EQR calculations.

Regarding our models, the only modeling we perform are the GLMMs used to produce the $R^2$ values for each simulation, the overall $R^2$ for the EEA vs. ERITS comparison, and a test of the influence of site distance in the EEA vs. ERITS model. The $R^2$ values reflect the goodness-of-fit of these models and are provided in the results and figures, but we have expanded the detail provided for the distance tests to provide more information about these models (edits shown in our response to your 4$^{th}$ comment above).

Furthermore, we wholeheartedly agree it is important to be able to easily reproduce as much of our results as possible. As mentioned, the EQR calculations cannot be reproduced without specialist assistance, but the analyses of these values can, as can our $R^2$ models. We therefore now provide both the data and code necessary to reproduce these analyses through the link in our data and code availability statements.

Original Lines 425–427: "...and (3) ecological quality, measured as the Ecological Quality Ratio (EQR) of each invertebrate community using country-specific methods (detailed in Supplementary Table 2)."

Edited to:

Current Lines 564–566: "*Ecological quality was measured as the Ecological Quality Ratio (EQR) of each invertebrate community, calculated using country- and river-specific methods and software (detailed in Supplementary Table 2).*"

**A few other comments:**

**- The authors appear to have written this for a general audience, which is appreciated. That said, the text reads as somewhat "arm wavy" in a few places. For example, lines 58 – 63 talk about the conservation implications, claiming new insights and sweeping implications but do little to support this grandiose language with evidence or specifics. This is something that carried on throughout the manuscript when discussing conservation implications: sweeping, broad statements about new insights into sampling strategy, with little consideration for on the ground consequences. I suggest either toning these statements**

**down or adding specifics to show concrete implementation. Otherwise, this is a classic case of academics dictating actions to practitioners (e.g., see literature on the knowing-doing gap, research-implementation gap, etc.).**

We edited several sections of our text to either remove vague statements regarding implications for conservation and monitoring, or to be more specific about how our results relate to these aspects.

Original Lines 58–64: "*Our results provide new insights into how common temporal data limitations affect biodiversity trend estimates. These findings guide efforts to estimate biodiversity change in freshwater, terrestrial, and marine ecosystems. Additionally, our results help guide authorities managing broad-scale monitoring programs (e.g., as part of the KM GBF or EU Water Framework Directive) by identifying whether additional investments of resources are essential to improve confidence in biodiversity change estimates*."

We edited the wording in this section to more closely match the content of our Discussion:

Current Lines 77–82: "*Our results show how infrequent or short-duration sampling can misrepresent biodiversity trends, providing empirical evidence of the value of long-term, high-resolution data. We further discuss the implications of these findings for broad-scale biodiversity monitoring (e.g., as part of the KM GBF or EU Water Framework Directive), which must consider how best to allocate limited resources to obtain accurate perspectives of biodiversity change.*"

Original Lines 172–175: "*Researchers, managers, and policymakers using biodiversity data and designing monitoring schemes must, therefore, ensure sufficient temporal resolution for assessing biodiversity status, making informed conservation decisions, and prioritizing effective actions.*"

The first paragraph of our Discussion contained the above broad statement about how our results inform monitoring. We removed this statement and replaced it with a more accurate one regarding how our results highlight the value of data with a high temporal resolution:

Current Lines 233–235: "*The importance of accurate estimates, and their applications, underscores the value of high-resolution temporal data and echoes repeated calls for more long-term ecological research*[31,33]*.*"

Original Lines 201–207: "*The former dataset suggests European river management efforts, which typically focus on controlling organic pollution*[32,33]*, successfully maintained biodiversity since the 2010s, although other stressors—such as micropollutants and climate change—likely need to be addressed to foment further improvements*[26]*. The latter dataset suggests that river management actions have broadly failed to prevent biodiversity loss, and additional measures are, therefore, critical for halting further declines.*"

Following your comments and those from another reviewer, we edited this section to be less hand-wavy about river management actions and more specific about how our results indicate the

potential for poor temporal resolution to lead to inaccuracies in determining the ecological status of individual rivers.

Current Lines 260–268: "*While differences in the overall outcomes were minimal, their contrasting directions and the generally differing perspectives of change between individual site pairs indicated the potential for data temporal resolution to influence local-scale management recommendations. For example, site-level ecological quality data reported as part of the EU Water Framework Directive (WFD) provides periodic updates on progress towards achieving a 'good' ecological status for European waterbodies. Within this framework where site-level assessments feed into regional and continental evaluations, trend discrepancies risk misdirecting local interventions and biasing broader status reports.*"

Original Lines 243–251: "*Such planning requires considering the needs and predicted limitations of a given monitoring scheme. Relying on data with a low temporal resolution allows for a broad-scale overview of trend direction, which reduces effort and cost but may come at the expense of trend value accuracy. In contrast, obtaining accurate, year-to-year estimates of the degree of biodiversity change across a decade or more, and ensuring, for example, > 70% trend value accuracy across sites, requires higher resolution information, such as gathering data annually or every two years across at least 80% of the desired time frame (e.g., 8 out of 10 years, Fig. 2; or 17 out of 20 years, see Supplementary Fig. 1).*"

Edits to this section were detailed in a response above, but we also highlight it here to further illustrate how we edited to focus more on our results, rather than general statements about monitoring:

Current Lines 308–317: "*For example, our results suggest that programs limited to low-resolution monitoring can still obtain an accurate, broad-scale overview of trend directions, but may fail to accurately estimate the rate of biodiversity change over time. We also found that trend estimates were less affected by declines in frequency than duration, as evidenced by higher site-by-site matches, higher explained variances, and lower differences compared to the annual trends. This result likely reflects the greater sensitivity of linear temporal trends to the initial and final years of a time series[26,60]. Reducing frequency still maintains coverage of both the early and late years of a time series, whereas reducing duration excludes either the earlier or later years entirely. Thus, reducing sampling frequency may be a cost-saving measure that provides higher trend accuracy compared to reducing sampling duration.*"

**- Interpolation of missing values with the mean of the neighboring values: This is artificially going to increase the apparent quality of the "raw" data. Biological trend data always have noise, which will reduce the goodness of fit for a line and increase the variation around the slope. Interpolation likely decreases the variability inherent to the data in a time series. Reducing sample frequency and time series duration increases variability (such as in the simulations). This may not overturn the overall outcome of the analysis, but it is worth noting, especially given that the authors are claiming this analysis is grounds for influencing continent-wide monitoring protocols. My hunch is that the results would be in stronger agreement (perhaps only moderately) with non-interpolated data (i.e., take the data as it is, no artificial corrections) or by choosing a random value to fill gaps (e.g., from**

**the range for the time series or within the bounds of neighboring points). The main text does not inform a reader that the time series being used as the basis of comparison (vs. simulated data) has been modified and this is somewhat misleading.**

We now make sure readers are aware of the gap filling at the start of the Results section (Current Lines 93–96), and we still detail how this was performed in our Methods. Furthermore, in the Discussion we now highlight the potential errors that can arise from gap-filling methods (Current Lines 321–325).

Original Lines 74–76: "*We compared trend estimates for the above metrics from the annual time series to those from simulated monitoring schemes with lower sampling frequency, lower duration, or both lower frequency and duration.*"

Edited to:

Current Lines 93–96: "*We quantified trends in the above metrics for 1,353 European river sites using annual data derived from nearly annual sampling with some gap filling. We then compared these trends to those from simulated monitoring schemes with lower sampling frequency, lower duration, or both lower frequency and duration.*"

Current Lines 321–325 (added): "*Additionally, if logistical or financial constraints preclude higher resolution monitoring, then complementary statistical approaches may help mitigate these issues, including gap filling[29] (as we used) or model-based methods to improve trend estimates from sparse data (e.g., hierarchical[68] or state-space[69] models). However, these approaches can also inflate error in some trend estimates[70].*"

**- Alternative approaches to manage low data coverage: The authors focus on a single recommendation for how to address the data degradation effects, which is to increase sampling frequency. I, and I imagine just about every other researcher, would be thrilled to see this become a reality but it is an unlikely outcome (broadly, at least). So, what other strategies can be used? What about more advanced statistical modeling options? Relating to the comment about interpolation, as well, the authors could consider approaches that account for missing samples and zeros in the data:**
**Blasco-Moreno, A., M. Pérez-Casany, P. Puig, M. Morante, and E. Castells. 2019. What does a zero mean? Understanding false, random and structural zeros in ecology. Methods in Ecology and Evolution 10:949–959.**
**Martin, T. G., B. A.Wintle, J. R. Rhodes, P. M. Kuhnert, S. A. Field, S. J. Low-Choy, A. J. Tyre, and H. P. Possingham. 2005. Zero tolerance ecology: improving ecological inference by modeling the source of zero observations. Ecology Letters 8:1235–1246.**

We agree that we can do a better job of acknowledging the statistical methods that have been developed to deal with data gaps and other biases. We now do so both in the Introduction (Current Lines 43–45) and Discussion (edits shown in our response to your previous comment).

Current Lines 43–45 (added): "*Various strategies have been developed to counteract these challenges, including statistical methods to address uncertainties and reduce biases arising from*

*low resolution data (e.g., gap filling[29]).*"

**- Another area absent from the manuscript is the ever-growing literature on how to measure biodiversity (e.g., work by Dr. Anne Chao and colleagues). Abundance as a measure is fraught and tightly linked to sampling effort. To a lesser but still substantial degree, so is richness. As a function of abundance and richness, EQR metrics have the potential to amplify biases inherent to these measures (but this is hidden by the lack of transparency on what the metric formulations entail – it is not reasonable to expect readers to go hunting for 18 different formulations in the literature). The authors give a general statement that they do not think these types of biases matter, but the treatment is unconvincing. This circles back to a theme, which is a general lack of specificity on many of the topics covered in the manuscript.**

We apologize for appearing to dismiss biases in the biodiversity metrics we used, which we believe was caused by our statement on Original Lines 270–272 that our results may apply to other biodiversity metrics. We edited this statement to clarify that we are only suggesting this may be the case and that further work would be required to confirm this is true (Current Lines 340–342).

Furthermore, to address your concerns about biases in abundance, richness and EQRs, we added an additional set of analyses to the supplementary whereby we repeated our simulations for coverage-based richness. We found no substantial differences compared to richness, which provides support for the conclusions from the other three biodiversity metrics. The details for this metric are now provided in Current Lines 86–90, 96–98, 558–564, and in Supplementary Figs. 1–2.

Original Lines 68–76: "*We examined the influence of lower sampling frequency and duration on trends in three broadly used community metrics that can indicate change in diversity or composition: (1) abundance (total number of individuals); (2) richness (total number of taxa); and (3) ecological quality, quantified as the Ecological Quality Ratio (EQR). Ecological quality is a widely used European measure of anthropogenic impacts on freshwater communities[27,28] based on the compositional similarity of sensitive and tolerant taxa to those from least-impacted reference communities in each region. We compared trend estimates for the above metrics from the annual time series to those from simulated monitoring schemes with lower sampling frequency, lower duration, or both lower frequency and duration.*"

Edited to:

Current Lines 86–90: "*We examined the influence of lower sampling frequency and duration on trends in four metrics that can indicate change in community diversity or composition: (1) abundance (total number of individuals); (2) richness (total number of taxa); (3) coverage-based richness (here the expected number of species if 80% of each community was sampled[45]); and (4) ecological quality, quantified as the Ecological Quality Ratio (EQR).*"

Current Lines 96–98 (added): "*We present results for abundance, richness, and EQRs in the main text, whereas those for coverage-based richness are provided in Supplementary Figs. 1–2*

*because they consistently mirrored the richness results.*"

Original Lines 270–272: "*This lower sensitivity was consistent across metrics of abundance, richness, and ecological quality, suggesting it is generalizable across studies that use different measures of biodiversity or ecological status.*"

Edited to:

Current Lines 340–342: "*This lower sensitivity was consistent across metrics of abundance, richness, coverage-based richness, and ecological quality, suggesting it may apply to other measures of biodiversity or ecological status, although this requires further confirmation.*"

Original Lines 424–427: "*We represented biodiversity for each site and year based on three community metrics: (1) total community abundance (number of individuals); (2) taxon richness (number of taxa); and (3) ecological quality, measured as the Ecological Quality Ratio (EQR) of each invertebrate community using country-specific methods (detailed in Supplementary Table 2).*"

Edited to:

Current Lines 558–564: "*We represented biodiversity for each site and year based on four community metrics: (1) abundance (total number of individuals); (2) richness (total number of taxa); (3) coverage-based richness; and (4) ecological quality. Coverage-based richness accounts for potential biases in richness caused by differences in abundance, such as sites or periods with greater abundance requiring greater sampling effort to collect the same proportion of taxa as communities with lower abundance[45]. We calculated coverage-based richness using the iNEXT package in R[76,77] with coverage set to 80% and $q = 0$.*"

**- Lines 29 – 36 are vague and fail to acknowledge the many ways that people try to counteract these challenges with sampling bias. This paragraph also fails to consider that many processes happen at scales beyond several years or a decade. Ironically, paleobiologists write these exact same sentences to critique biological time scales and justify the need for an even more extensive temporal perspective (an issue that at least a few of the example studies do acknowledge, e.g., Dornelas et al. 2014 on the topic of shifting baselines).**

We thank the reviewer for this comment, which aligns with a similar comment raised by Reviewer 1. We revised this paragraph to better acknowledge existing strategies to counteract sampling bias and to clarify that ecological processes often occur at temporal scales longer than a decade.

Original Lines 29–36: "*Biodiversity data often suffer from a variety of limitations, with one common limitation being a low temporal resolution. This limitation can result from opportunistic data collation or logistical monitoring constraints. Consequently, many broad-scale biodiversity studies quantify trends using data with a low sampling frequency or duration, such as just two or three sampling years per site[6,7,9,13]. Given that communities exhibit natural interannual*

*variability, using such sparse time series can produce the appearance of a trend where there is none or vice versa*[22,23] *(Fig. 1). These biases could lead to incorrect conclusions and misguided management recommendations.*"

Edited to:

Current Lines 29–48: "*Biodiversity data often suffer from a variety of limitations that could lead to disagreement and unreliability in trend estimates, including spatial biases and heterogeneity in data characteristics*[16,23]*. One common limitation is low temporal resolution. This limitation typically results from opportunistic data collection, or monitoring constraints of funding, time, and personnel. Consequently, many broad-scale biodiversity studies quantify trends using data with a low sampling frequency or duration, such as just two or three sampling years per site*[7,8,10,14]*. The common use of such sparse time series could bias biodiversity trends in two principal ways. First, temporal trends quantified from a low number of samples have low statistical power, defined in this context as a low probability of detecting the correct trend value*[24,25]*. Second, because communities exhibit natural interannual variability, using low-resolution time series can produce trends that reflect stochastic fluctuations rather than real change*[19,26] *(Fig. 1a, b). These two sources of trend bias have important implications because they may accumulate when trends are aggregated across multiple sites (Fig. 1c, d), inflating error rates and leading to incorrect conclusions about biodiversity change that waste limited conservation resources and misinform management recommendations*[20,27,28]*. Various strategies have been developed to counteract these challenges, including statistical methods to address uncertainties and reduce biases arising from low resolution data (e.g., gap filling*[29]*). However, such approaches may only partly compensate for low temporal data resolution, particularly given that obtaining accurate temporal trends can require consistent monitoring for at least a decade*[30,31] *or even longer*[28,32–35]*.*"

**A few comments on figures:**
**Figure 1 – I like the schematic to help with interpretation. Perhaps add comparable visuals for the analytical metrics: direction, magnitude, R2? Though it may vary from reader to reader, the existing version of Figure 1 includes the components I found easiest to understand (time series sampling duration and frequency).**

Good idea! This would greatly help readers prepare for the upcoming results they are about to see. We now include both a "within-site" set of examples for frequency and duration, which are the original panels from Fig. 1, and we added two new panels to demonstrate how these within-site trend biases could scale up to multiple site-by-site trend mismatches (Fig. 2) and error in across-site trend estimates (Fig. 3).

Additionally, we complemented Table 1 in the Methods, which listed the different analyses, with a new Fig. 5 that provides a visual guide to our simulation methods. A clearer explanation of how the direction & magnitude matches, and $R^2$ calculations, were performed helps readers better link the new panels in Fig. 1 to our analytical methods.

New Fig. 1:

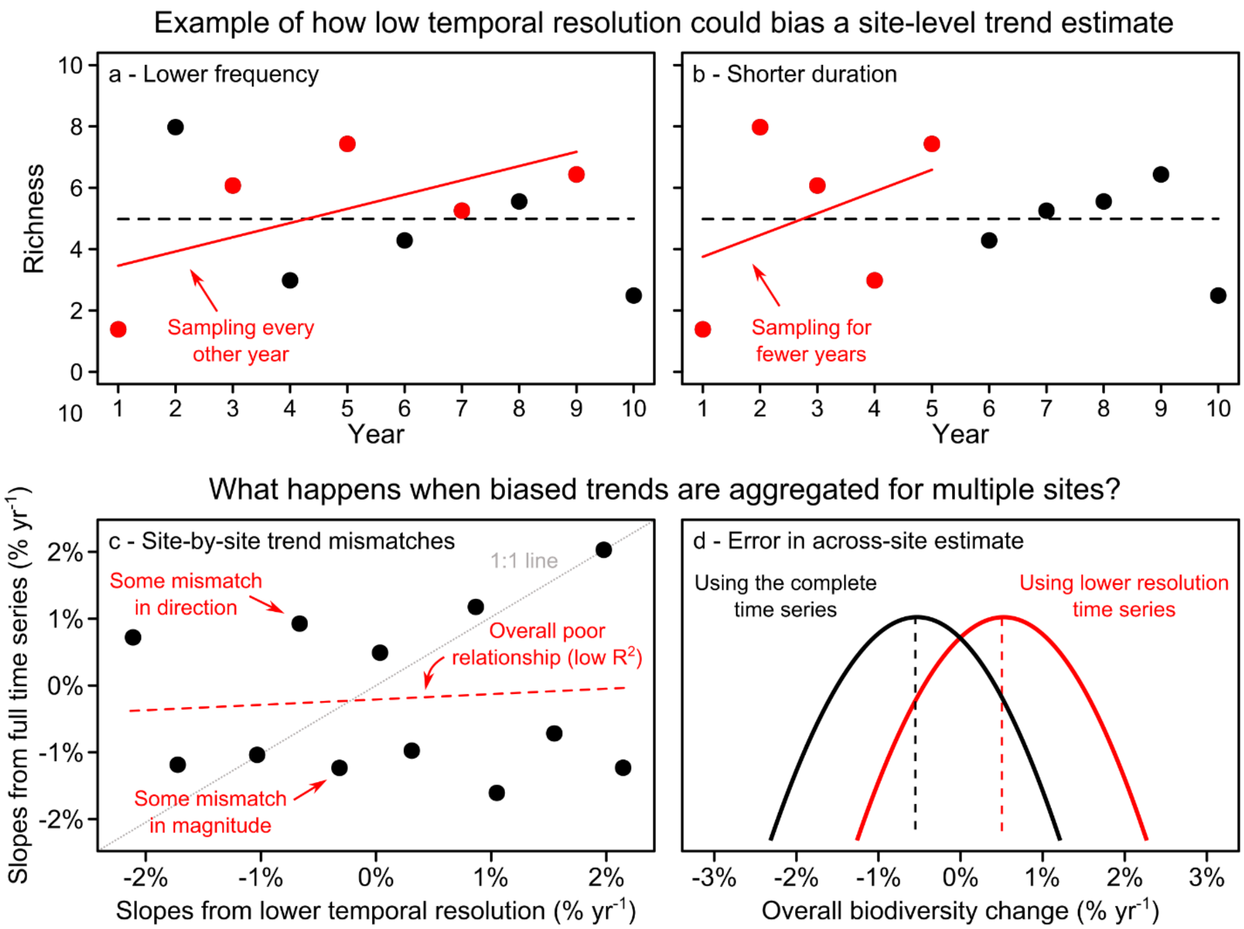


***Figure 1 Theoretical examples of how temporal data limitations may affect biodiversity trends.*** *Reducing the* ***(a)*** *sampling frequency or* ***(b)*** *sampling duration of a time series can cause estimated trends (red lines) to deviate markedly from those quantified using a higher temporal*

*resolution (black, dashed lines). These biases could lead to* ***(c)*** *an accumulation of numerous trend direction and magnitude errors when trends are aggregated among multiple sites, driving* ***(d)*** *differences in across-site estimates of biodiversity change (e.g., the average and distribution of trend values).*

New Fig. 5:

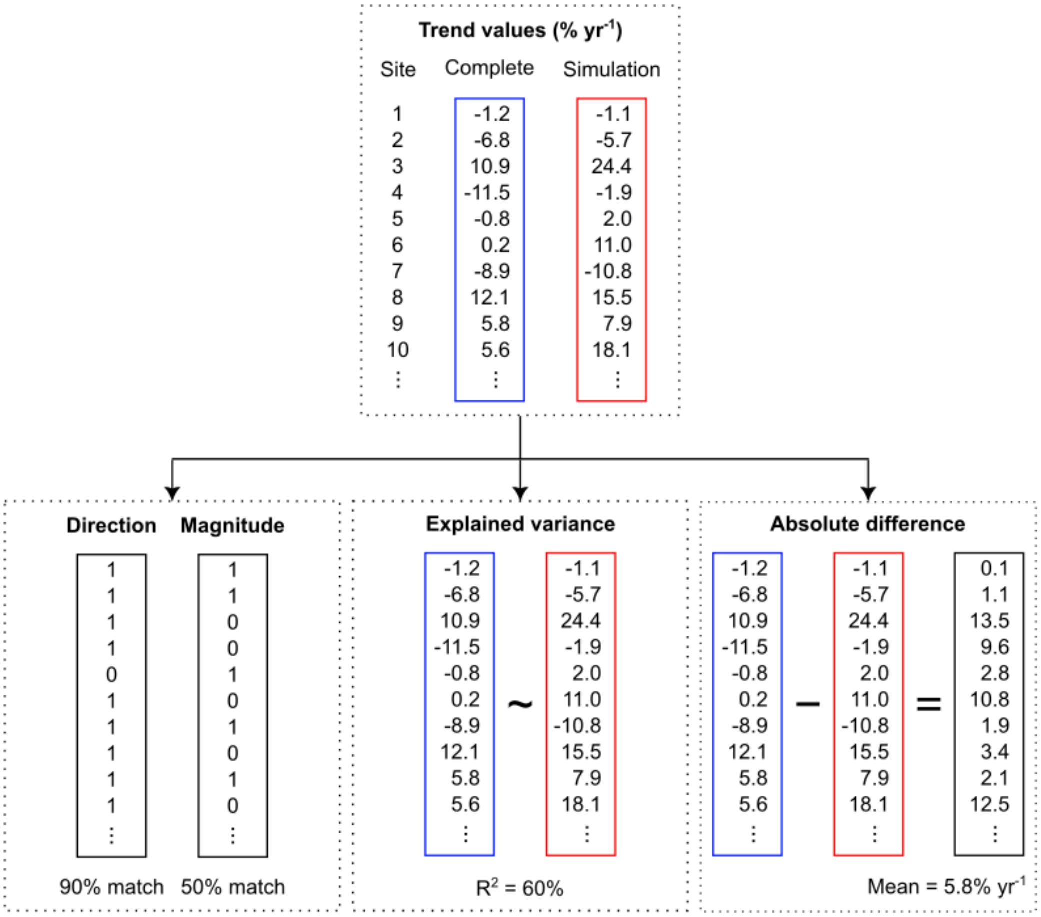


***Figure 5. Illustrative example of how the simulation results were produced.*** *Methods used to quantify the trend direction and magnitude matches,* $R^2$*, and differences for each of 1,000 simulations for the different frequency, duration, and frequency & duration simulations. Direction matched (0 = no match, 1 = match) if the 'complete' trends (i.e., trends from the annual time series or the first 10 years for duration alone) and simulated trends had the same*

*direction. Magnitudes matched if the simulated trends fell within the standard error. $R^2$ reflects the proportion of trend variance captured by the simulations, quantified using GLMMs. Differences represent the average deviation in the simulated trend values, quantified as the across-site geometric mean of the absolute differences.*

**Figure 2 – This is a nice, visually pleasing figure. In the legend, perhaps change the color of the SD circles. Because they are black, and because R2 is also black and shows the largest values, it appears as if SD only applies to R2. Changes in SD for direction and magnitude (assuming there are any) are washed out. Size of points also is not mentioned in the caption, but colors are, so it might be worth adding. In this figure, SD shows a pattern of reaching max values at intermediate Duration levels. This does not appear to have been mentioned by the authors but may be worth some interpretation. I also was surprised to see a more complicated relationship (sigmoidal?) in the longer time series included in the supplement. This also was not discussed, which seems like a missed opportunity.**

We added the point colors to the SD legend in Fig. 2 as suggested, and we added associated text to the caption. Regarding the SD patterns, our response to your second comment should address this concern. The somewhat sigmoidal relationship is primarily driven by the generally poor matches (and poor $R^2$) across all simulations once the amount of data has declined to a certain point (e.g., beginning around 2–4 years in the 10-year duration simulations, or 4–6 years in the 20-year simulations). There is also an indication of a possible asymptote at higher duration values in the 20-year EQR results, but this does not occur in the other metrics so we did not focus on this result in the text.

**Figure 3 – Again, a visually appealing figure, however, I found the data challenging to evaluate because of the differences in the scales on the x-axis. Scales go from tenths to tens, with everything in between. I understand (or assume) that the point is to show reduced agreement with sample degradation but it is hard to judge the extent to which this is meaningful when neighboring plots show one value dropped by 0.05% and another by 50%, but visually they appear identical. A log-transformation could help with this. More minor: consider removing the interspersed y-axis labels (frequency, duration, frequency & duration). This would match figure 2 and improve the readability.**

We made several modifications to this figure to more clearly display the differences between the trend estimates derived from the complete time series (annual or 10-year) and those from the simulations. First, we now present the *absolute* differences in the trend estimates, rather than the

raw differences. Using raw values sometimes masked the magnitude of the differences because opposing positive and negative errors could offset each other, making the overall difference appear smaller than it actually was. Second, we have unified the scales of the x-axes where possible. Unfortunately, not all panels can use the same scale, even when log-transformed as they are currently, due to wide variation in difference values between abundance and richness/EQRs. However, the richness and EQR panels now share the same x-axis scales in the same columns. We also removed the y-axis labels as suggested. Lastly, we now contextualize the meaning of the differences readers are seeing when these results are first mentioned (Current Lines 159–162). This change clarifies how a difference of, for example, 0.2% versus 2% should be understood.

Current Lines 159–162 (added): "*To help contextualize these errors, overall biodiversity change across realms or taxonomic groups often falls within just 0–2% per year*[10,14,43]*, meaning deviations of even 1% can translate to a large misestimate of the overall degree of biodiversity change.*"
New Fig. 3:

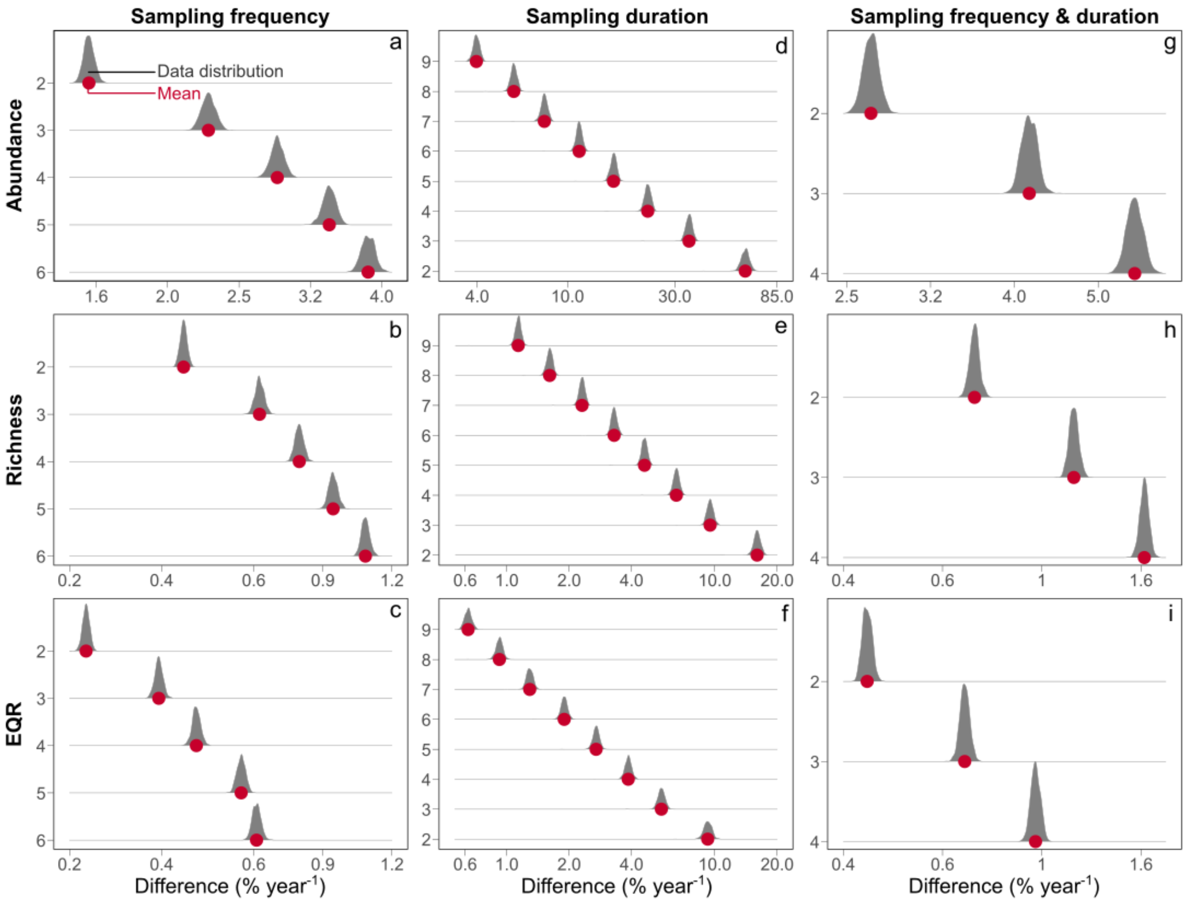


***Figure 3. Influence of lower sampling frequency and duration on differences in biodiversity trends.*** *Lower (**a–c**) sampling frequency, (**d–f**) sampling duration, and (**g–i**) a combination of lower frequency and duration tended to increase the differences in trend values between the simulated monitoring schemes and those from the complete time series (i.e., annual or the first*

*10 years for duration alone). Trend differences are shown using density plots of the average, across-site absolute difference for each simulation (shaded polygons) and the average of these differences across simulations (filled circles). Note that all x-axes are on the $log_{10}$-scale and that the range of this scale varies across panels.*

**Figure 4 – Panel (a) would benefit from more explanation. Taken independently, I am not sure what I am supposed to take from this part of the figure.**

Panel a shows the same direction and magnitude matches, and $R^2$ of the relationship of the trend estimates, as shown in Fig. 2. We edited the caption to clarify these details.

Original Fig. 4 caption: "***Comparison of estimated EQR trends between two datasets that differ in temporal resolution.*** *Differences in temporal resolution between the EEA and the ERITS sites influenced (**a**) the percentage of matches in site-level trend estimates and the across-site data distribution for the (**b**) ERITS and (**c**) EEA sites. The density plots show the probability distribution of trend estimates for the 485 sites each, dots and error bars represent the mean and standard deviation, and the upper boxes show the median, 50, 75, and 95% of the data. Red dots show data out of the 50% percentile.*"

Edited to:

Current Fig. 4 caption: "***Comparison of estimated EQR trends between two datasets that differ in temporal resolution.*** *Differences in temporal resolution between the EEA and the ERITS sites influenced: (**a**) matches between the direction and magnitude of site-level ecological quality trends (EQR change year$^{-1}$), and the $R^2$ of their relationship (based on a GLMM); (**b**) trend estimates, represented as the absolute differences between the EEA and ERITS slope values; and (**c, d**) the average, across-site estimates of ecological quality change. Density plots in (**c, d**) show the probability distribution of trend estimates for the 485 sites, dots and error bars represent the mean and standard deviation, and the upper boxes show the median and distributions for 50%, 75%, and 95% of the data. Red dots show data outside 50%.*"

**Overall, I enjoyed this manuscript and look forward to seeing it published. A few of the analyses could use some little fine tuning (especially EQR trends for EEA vs ERITS) and I think the manuscript (including methods and supplement) would benefit from more concrete language, specifically in the introduction and discussion. That said, the manuscript is well written and logically structure, and the authors have processed an impressive amount of data. Critiques aside, I whole-heartedly agree with the message in the manuscript! - Jansen Smith**

**Reviewer 3**

**The manuscript entitled 'Robust estimates of biodiversity change require high-resolution time series' presents an extensive collection of freshwater time series to investigate the impact of two temporal sampling biases on biodiversity measures assessment: frequency and duration. The author demonstrates the impact of such biases using extensive simulation analyses from the existing time series they collected. Additionally, they compared biodiversity trends between some of the sites from their time series with those of the EEA. The authors also make a bridge beyond their research work to connect to decision-making bodies that depend on such research work by providing guidelines and uncertainty considerations.**

**Overall, I found the article well-organized and well-written. It highlights very well existing temporal data biases in biodiversity surveying that can heavily impact the conclusions of time series analyses. I believe that such work provides a clear and good structure in the analysis of freshwater data, which could open perspectives for other realms.**

**I have first general comments or questions that focus on the general understanding and the method, given that I am not familiar with freshwater ecology:**

**1) This is a remark for consideration: I agree with you and with your work questioning the reliability of data. I think there are times where the authors could have been slightly more cautious in their statement because not all papers showing no trends or discrepancies use the same methodology or the data in the same way. There is complexity in statistical analyses as well as in the context where the study is done (spatio-temporal extent, for example), see Boennec et al (2024).**

**Boënnec, M., Dakos, V. and Devictor, V. (2024), Sources of confusion in global biodiversity trends. Oikos, 2024: e10732. https://doi.org/10.1111/oik.10732**

We agree there are a number of factors that can influence biodiversity trend estimates, and we edited the opening of the second paragraph of our Introduction to clarify that our study only investigates one of these many factors (temporal resolution).

Original Lines 29–30: "*Biodiversity data often suffer from a variety of limitations, with one common limitation being a low temporal resolution.*"

Edited to:

Current Lines 29–31: "*Biodiversity data often suffer from a variety of limitations that could lead to disagreement and unreliability in trend estimates, including spatial biases and heterogeneity in data characteristics*[16,23]*. One common limitation is low temporal resolution.*"

**2) Another aspect that I wished the authors had pointed out is the difference between realms. I agree that the generalization is also important, but thinking about the differences (collection, identification of species difficulties) and the lack of global studies on freshwater,**

**this is something that could have been discussed more and thus highlighting the novelty of the extensive work done here. If possible, the authors could consider adding a few sentences in the introduction and discussion about this.**

You raise a good point that would help highlight the novelty of our work. We added some text to the Introduction to better outline our contribution to understanding freshwater ecosystems (Current Lines 64–70). We also modified the section of the Discussion to clarify that our results may differ among taxonomic groups and realms and how our dataset helps minimize uncertainties that affect other biodiversity research, thus better highlighting its novelty (Current Lines 296–303).

Original Lines 48–51: "*This dataset presents a unique opportunity to investigate the effect of temporal limitations on biodiversity trend estimates, given the general scarcity of biodiversity data that combines community-level information across a broad spatial scale with high temporal resolution.*"

Edited to:

Current Lines 64–70: "*This dataset presents a unique opportunity to investigate the effect of temporal limitations on biodiversity trends, given the general scarcity of biodiversity data that combines community-level information across a broad spatial scale with high temporal resolution. Additionally, freshwater ecosystems tend to be underrepresented in global biodiversity datasets (with some exceptions[14,43]), and conservation in general[44], thus our study provides insights into how temporal limitations affect biodiversity assessments in a generally underrepresented realm.*"

Original Lines 232–238: "*Indirect uncertainties can arise for numerous reasons, such as datasets aggregating information from incomparable study designs or sampling methods[15–17], or combining taxonomic groups with substantially different population growth patterns[19,39,40]. We did not focus on these issues because they are not a factor in our data, given that we examined the same taxonomic groups collected using comparable methods across sites (i.e., invertebrates sampled from the river bottom). However, we acknowledge the need to evaluate and control indirect uncertainties in biodiversity research and monitoring.*"

Edited to:

Current Lines 296–303: "*Indirect uncertainties can arise for numerous reasons, such as datasets aggregating information from incomparable study designs or sampling methods[16–18,23], or combining taxonomic groups with different population growth patterns[20,26,58]. Our dataset provided a valuable opportunity to minimize these issues because sites used similar methods to sample the same taxonomic group from the same ecosystem type (i.e., invertebrates sampled from river bottoms). However, we acknowledge the need to evaluate and control indirect uncertainties in other datasets, taxonomic groups and realms, and for biodiversity research and monitoring in general.*"

**3) All main analyses are done on an extensive number of sites, but testing is only gradually**

**done every 2 to 6 years, 9 to 2 consecutive years of sampling, but is this done only for 10-year time series. This is only mentioned in the method section (lines 480-482), which is a bit late, I think. Why choose this 10-year extent and not 20 years (even if it meant fewer sites)? The 10-year extent is one of the limitations since previous work from the literature mentions that longer time spans are needed to highlight temporal changes. This is not discussed, if I recall as well.**

We considered different potential time frames, but our results were generally the same whether we included shorter time series (1,353 sites with at least 10 years) versus only using longer time series (255 sites with 20 years or more; shown in Supplementary Figs. 3–5), so we opted to include the 10 year time series as it provided a more robust and broader-scale dataset.

To address your concerns, we moved the section that mentions the 20-year results into the Results (rather than the Methods as before), so it comes much earlier in the text (Current Lines 177–189). We also now highlight the value of longer time series in both the Introduction (Current Lines 45–48), the now added section of the Results, and the Discussion (Current Lines 290–294).

Current Lines 45–48 (added): "*However, such approaches may only partly compensate for low temporal data resolution, particularly given that obtaining accurate temporal trends can require consistent monitoring for at least a decade*[30,31] *or even longer*[28,32–35]."

Current Lines 177–189 (added): "*An important caveat to the above results is that we restricted our dataset to time series spanning a decade or more (detailed in our Methods). However, even a decade can be insufficient for obtaining accurate trend estimates*[23,28,34,35]*. To test whether longer time series are more robust to data loss, we repeated our analyses for a subset of 255 sites out of 1,353 total with at least 20 years of data and expanded the duration simulations to 2–19 years in comparison to the first 20 years. We found the same overall results (Supplementary Figs. 3–5), indicating our conclusions were generally consistent whether shorter or longer time series were used. A notable exception was the lower sensitivity of* $R^2$ *to declines in frequency and duration. Specifically, changes in* $R^2$ *tended to mirror changes in the magnitude matches in our full analyses (Fig. 2); however, when limited to time series with at least 20 years of data,* $R^2$ *did not decline as sharply as magnitude (Supplementary Figs. 3, 4). These patterns suggest that, while trend magnitudes are still sensitive to data loss, longer time series may better preserve trend variation among sites even at lower sampling frequencies and durations.*"

Current Lines 290–294 (added): "*However, depending on the taxonomic group and ecosystem in question, biodiversity change may be better represented by non-linear dynamics (e.g., in ecosystems that experienced a regime shift*[55,56]*), annual sampling may not be necessary*[36]*, and multiple decades of monitoring may be needed to fully represent biodiversity change*[31,34,35]."

**4) The term 'across sites' is extensively used for part of the analyses I wonder if changing it to regional or national, since it is country-wise, would make more sense. This will also help the authors to highlight results at two spatial scale, local (site) and national (across sites).**

We appreciate your suggestion highlighting the strengths of our approach. However, we chose to

not frame our different analyses as “local-scale” versus “broad-scale” (e.g., local versus national, continental, regional, etc.) because all are broad-scale in some sense. For example, the direction and magnitude results are derived from local, site-by-site comparisons, but the overall percentage match reflects a broad-scale result. We considered the “site-by-site” and “across-site” terms as more appropriate because they are closer to describing the methods of how the different metrics were calculated.

To help readers develop a more intuitive understanding of this wording, we now present it earlier in the text in a modified version of Fig. 1, which introduces readers to what we mean by site-by-site comparisons versus across-site estimates. We also edited two sections of the Results to clarify that “across-site” refers to when an average is calculated across sites (Current Lines 140–145 and 196–198). Lastly, we added an illustrative example of how the different results were produced in the Methods in a new Fig. 5 to more clearly outline for readers how the direction & magnitude matches, $R^2$, and trend differences were calculated through a combination of local site-by-site comparisons that produce an overall, broad-scale value.

Original Lines 113–116: “*Lower sampling frequency and duration also led to greater error in across-site estimates of overall biodiversity change, with error represented as the average difference in the across-site trend estimate for the complete time series (or the first 10 years for duration) versus the same estimates from the simulated monitoring schemes (Fig. 3).*”

Edited to:

Current Lines 146–151: “*Lower sampling frequency and duration also led to greater error in biodiversity trend estimates, represented as the average of the absolute difference between the simulated versus complete trend estimates (i.e., % change per year in abundance, richness, or EQR). This comparison assesses, across all sites, exactly how much the simulated trends deviated from those derived from the complete time series, complementing the direction, magnitude, and $R^2$ results presented above.*”

Original Line 148: “*We compared EQR trends during 2010 to 2023.*”

Edited to:

Current Lines 196–198: “*We compared trends between paired, proximate sites based on direction matches, magnitude matches, $R^2$, and differences as above, and based on the density distribution and the across-site average and SD of their trend values.*”

New Fig. 5:

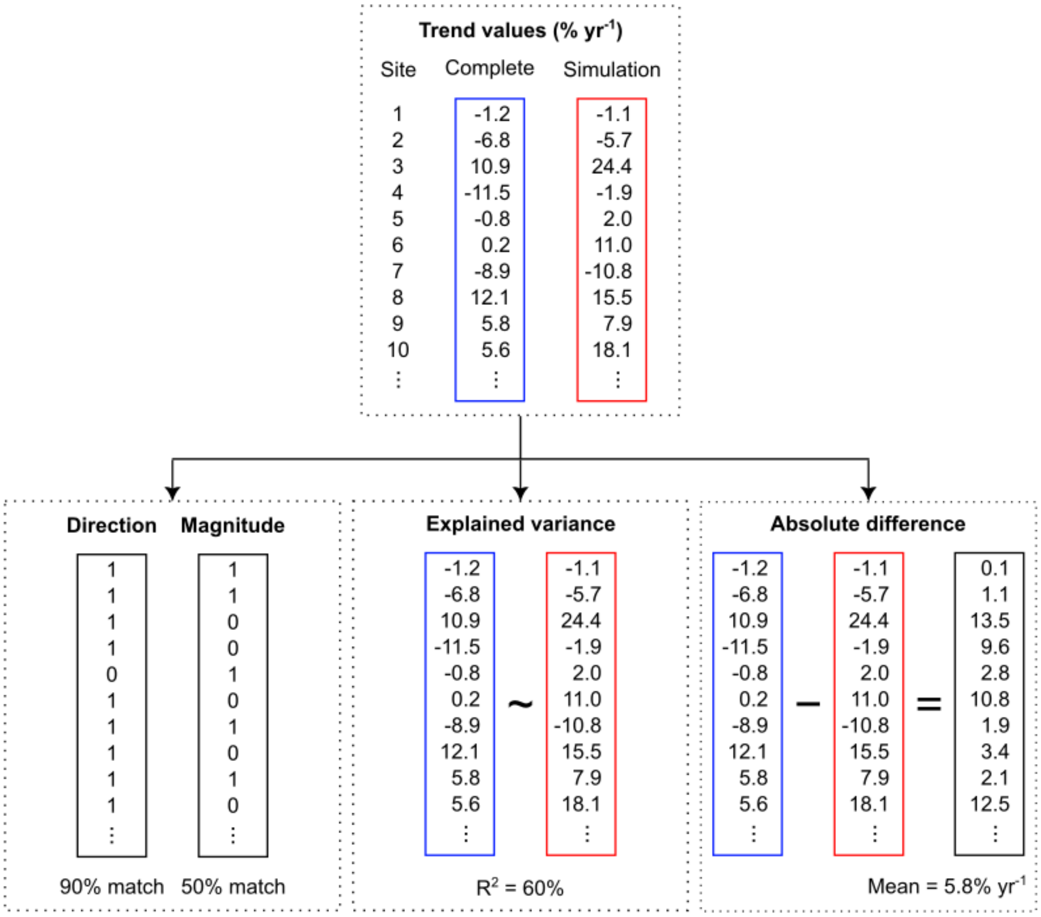

Trend values (% yr-1)
Site
Complete
Simulation
1 -1.2 -1.1
2 -6.8 -5.7
3 10.9 24.4
4 -11.5 -1.9
5 -0.8 2.0
6 0.2 11.0
7 -8.9 -10.8
8 12.1 15.5
9 5.8 7.9
10 5.6 18.1
Direction
Magnitude
1 1 1 1 0 1 1 1 1 1
1 1 0 0 1 0 1 0 1 0
90% match
50% match
Explained variance
-1.2 -6.8 10.9 -11.5 -0.8 0.2 -8.9 12.1 5.8 5.6
~
-1.1 -5.7 24.4 -1.9 2.0 11.0 -10.8 15.5 7.9 18.1
R2 = 60%
Absolute difference
-1.2 -6.8 10.9 -11.5 -0.8 0.2 -8.9 12.1 5.8 5.6
−
-1.1 -5.7 24.4 -1.9 2.0 11.0 -10.8 15.5 7.9 18.1
=
0.1 1.1 13.5 9.6 2.8 10.8 1.9 3.4 2.1 12.5
Mean = 5.8% yr-1

***Figure 5. Illustrative example of how the simulation results were produced.*** *Methods used to quantify the trend direction and magnitude matches, $R^2$, and differences for each of 1,000 simulations for the different frequency, duration, and frequency & duration simulations. Direction matched (0 = no match, 1 = match) if the 'complete' trends (i.e., trends from the annual time series or the first 10 years for duration alone) and simulated trends had the same direction. Magnitudes matched if the simulated trends fell within the standard error. $R^2$ reflects the proportion of trend variance captured by the simulations, quantified using GLMMs. Differences represent the average deviation in the simulated trend values, quantified as the across-site geometric mean of the absolute differences.*

**5) Finally, did the author also consider other biodiversity metrics such as taxa turnover? The results shown in the current manuscript could be even more dramatic for such metric. Another reason for this question is that for readers not familiar with freshwater realms, EQRs are very uncommon diversity measures.**

We settled on abundance, richness, coverage-based richness (added per Reviewer 2's suggestion), and EQRs because they encompass different aspects of community diversity and composition, and are compatible with our simulation approach. We did initially consider other metrics, including temporal turnover, but this metric poses several challenges. Our simulations extract values for selected frequencies or durations from each time series (i.e., what would the values be if we only had data from those years?). Turnover cannot be handled this way because it requires data from a previous year to calculate, which changes in each simulation. This presents two problems. First, many time series simulations only have two years of data, but at least three years are required for turnover because the first year selected for the simulation also requires compositional values from a previous year. Second, even if we only calculated turnover for simulations that have enough data, these calculations still require compositional data for each year. However, some years were gap filled and so lack these data. Gap filling compositional data requires gap filling abundances for sometimes dozens of taxa, which would introduce much larger biases than interpolating single values of total abundance, richness, or EQRs. For these reasons, we elected to not include turnover as a metric in our study.

**I have some small suggestions that might help to understand part of the work below. Note that some are suggestions and do not require strict changes if you disagree. There is a part of subjectivity in the terminology and the interpretation we have of them; I am only highlighting the ones that stood out for me.**

**<u>Abstract:</u>**

**line 3: 'poor availability' has a very negative and strong connotation to me that goes beyond the capabilities and the limitations that could exist when researchers collect data (or if they are actually open access). I encourage the reviewers to find an alternative term or to revise the sentence to highlight more temporal biases rather than the availability.**

You are right. The term "poor availability" may sound overly negative. We altered it to "temporal data limitations".

Original Lines 3–4: "*However, questions remain about whether the poor availability of high-resolution time series compromises trend accuracy.*"

Edited to:

Current Lines 3–4: "*However, common temporal data limitations may compromise trend accuracy.*"

**lines 7-10: Compared to the real example, the summary of the simulation results is quite vague. I suggest having a comparable statement or giving a similar level of detail.**

We modified the abstract to provide more even and informative results from both the simulations and the dataset comparison.

Original Lines 6–13: "*We simulated lower sampling frequencies, shorter durations, and combinations of both, then compared these trends to the complete, annual time series. Here we show that accurate estimates of both the direction and magnitude of biodiversity change require high-resolution time series, whereas lower resolution data may only provide reliable direction estimates. As a real-world example, we compared 485 sites from our dataset with lower resolution monitoring data from the European Environment Agency and found a 53% match in trend directions but only a 12% match in trend magnitudes. These differences would result in opposite management actions.*"

Edited to:

Current Lines 6–12: "*We simulated reduced sampling frequencies and durations and compared these trends to the complete time series. Reducing frequency from annual to every 2–6 years resulted in 87–74% of sites matching in trend directions, but only 82–48% matching in trend magnitudes. Reducing duration from 10 to 9–2 years resulted in 88–53% direction matches and 89–12% magnitude matches. Similar results were observed for longer time series (≥ 20 years). Additionally, we compared two real-world monitoring datasets with different temporal resolutions and found 53% of sites matched in direction, but only 12% in magnitude.*"

**line 16: Why local scale management only, you also have evaluated more global biodiversity impact.**

Our intention was to distinguish between global conservation targets and local conservation actions, which require different management decisions. To address your concern, we moved this sentence to the start of the abstract and now we just refer to conservation and management in general.

Original Line 2: "*Addressing ongoing global biodiversity loss requires reliable trend assessments.*"

Replaced with:

Current Lines 2–3: "*Robust estimates of biodiversity change are essential to inform management and conservation, and to address global biodiversity loss.*"

**Introduction:**

**lines 23-25, I believe this statement could be stronger with references, even if it is linked to the previous sentence.**

We moved the references from the previous sentence to this sentence. The growing number of biodiversity studies can be taken as a given, whereas the sentence that follows would benefit from the citations, as you point out.

Original Lines 22–25: "*...and by the growing number of broad-scale (i.e., global or continental) studies of biodiversity change in terrestrial, marine, and freshwater ecosystems*[6–14]*. However, such studies commonly report finding an approximately equal mixture of neutral, increasing, and decreasing site-level biodiversity trends.*"

Edited to:

Current Lines 22–25: "*...and by the growing number of broad-scale (i.e., global or continental) studies of biodiversity change in terrestrial, marine, and freshwater ecosystems. However, such studies commonly report finding an approximately equal mixture of neutral, increasing, and decreasing site-level biodiversity trends*[6–15]."

**line 30: 'collation' to be changed to 'collection'**

This change has been made (Current Line 32).

**line 42: correct the citation: 'refs. 13 versus 26'**

This change has been made (Current Lines 56–57).

**Results**

**line 70: In this result section the author mentions (1) abundance (total number of individuals). I think for a better understanding and to avoid any questions before going to the method section, it should be stated the same way as it is in the method: (1) total community abundance (number of individuals).**

**The same goes for the second measures: in results: (2) richness (total number of taxa) and in the method section: (2) taxon richness (number of taxa).**

These phrasings now match between the Results and Methods, which are:

"*...abundance (total number of individuals); (2) richness (total number of taxa)...*"

**line 71: As mentioned above, I am not familiar with EQR, but other measures of community composition change could have been considered here. Turnover has been, for example, extensively used in that context for other taxa and realms.**

Our response to your comment #5 above should address this concern.

**There is some overlap between the results presented in the second and third paragraphs. You could potentially simplify and or combine these. This is a suggestion to improve the readability of the results.**

We consolidated these two paragraphs into one as suggested. We outline these edits only using the new, single paragraph for brevity:

<u>Current Lines 102–121</u>: "*Across all community metrics, decreasing sampling frequency increased the site-level trend 'error rate', meaning a decrease in the degree to which estimated trend values for each site matched those obtained from annual sampling frequencies for the same sites. For example, decreasing sampling from annual to once every 2–6 years resulted in trends that matched the direction (i.e., positive or negative) of the annual trends in, respectively, 87–74% of sites (percentages reflect the average across metrics; Fig. 2a–c). Compared to the direction matches, error rates increased more severely for the magnitude matches, determined as whether simulations fell inside the standard error of the annual trends, with matches decreasing from 82 to 48%. Likewise, the $R^2$ of the relationship between the simulated and annual trends (i.e., whether the simulated trends accurately captured among-site trend variation) also declined substantially as frequency declined, from 0.83 to 0.40. These values reflect the potential to mischaracterize biodiversity change in many individual sites, even for sampling frequencies that exhibited the least error. For example, the highest match percentage (87% for direction at a frequency of every 2 years) still corresponds to obtaining the wrong trend direction for 176 sites out of 1,353 total. Additionally, we can infer that the more severe increases in magnitude error rates (and declines in $R^2$) as frequency declined were driven by large magnitude shifts that were in the same direction as the annual trends, given that trend directions were less affected, meaning the magnitude errors were generally not caused by positive trends shifting to negative or vice versa. The magnitude shifts also had to be large to fall outside the standard errors of the annual trends.*"

**line 130: I am not sure adding the estimate per decade in addition to the ones per year is necessary.**

We removed the decadal estimates as suggested.

<u>Original Lines 118–121</u>: "*For example, the average, across-site estimates of biodiversity change from the complete annual time series were 0.37%, 0.79%, and 1.1% year$^{-1}$ for abundance, richness, and EQR, respectively (corresponding to 3.7%, 7.9%, and 11% per decade).*"

Edited to:

<u>Current Lines 153–155</u>: "*For example, the average, across-site estimates of biodiversity change*

*from the annual time series were 0.37%, 0.79%, and 1.11% year$^{-1}$ for abundance, richness, and EQR, respectively.*"

Original Lines 127–130: "*For example, the average, across-site trend estimates for the first 10 years were 1.6%, 1.1%, and 1.5% year$^{-1}$ for abundance, richness, and EQRs, respectively (corresponding to 16%, 11%, and 15% per decade).*"

Edited to:

Current Lines 164–166: "*For example, the average, across-site trend estimates for the first 10 years were 1.56%, 1.14%, and 1.44% year$^{-1}$ for abundance, richness, and EQRs, respectively.*"

**line 147: how spatially proximate? (Maybe this information could be summarized from the method section, for example, less than 1km)**

We now provide the average distance in the Results, and the average and range in the Methods. We used 1 km as an initial selection threshold to identify sites in the same area, but no site pairs were actually this far apart from each other. Our added detail now clarifies this in the Methods.

Original Lines 146–148: "*We ensured the EEA sites were spatially proximate and in the same rivers as their paired ERITS sites.*"

Edited to:

Current Lines 198–201: "*We ensured that the paired ERITS and EEA sites were located in the same rivers, were spatially proximate (average distance of 3.1 m), and that the sampling years for the EEA sites always fell within the sampling years of their paired ERITS sites.*"

Original Lines 495–497: *"...that were sampled from the same rivers and were within at least 1 km of each other (average distance of 3.1 m; Supplementary Fig. 2b)."*

Edited to:

Current Lines 616–619: "*To determine the site pairs, we first used a cutoff distance threshold of 1 km to identify sites in the same geographic area. We then selected only site pairs that were the closest to one another and within the same waterbody (average distance between pairs of 3.1m; range 1.2–114.6 m; Supplementary Fig. 6b).*"

**Discussion**

**line 180: If considering a terminology change, I mention in the method section (across site to regional or national), it will help highlight the multi-scale aspect of this study.**

The edits shown in our response to your comment #4 above should address this concern.

**line 224: I think it is good that you have acknowledged some potential limitations, consider**

**adding the one I have mentioned as well in this review, and add them here or later in the discussion.**

Based on comments from all three reviewers, we substantially revised this paragraph to provide a more comprehensive account of our limitations. The changes relevant to your concern are shown in our response to your comment #2 above.

**Method:**

**Generally, a couple of method parts are presented but not the results themselves (see below).**

**line 400: If possible, add the number of time series.**

We added the total number of time series from which we made our selections.

Original Lines 400–401: "*We obtained time series data on European river invertebrate communities from refs. 48 and 49, and from further requests to ecologists and environmental managers.*"

Edited to:

Current Lines 527–528: "*We obtained a total of 4,050 time series of European river invertebrate communities from refs. 72 and 73, and from further requests to ecologists and environmental managers.*"

**lines 406-408: Does this mixed identification resolution also impact the result? This could be added to the discussion.**

Good point! To address your concern, we now partly test for this issue in a new supplementary analysis that excludes sites with taxa identified only to the family-level or higher. We found the same results as our full analysis, suggesting our results are unlikely to change even if we had data with a finer taxonomic resolution. This is not a perfect test of course since none of our datasets are entirely at the genus- or species-level, which is extremely rare in invertebrate research, but it provides some support for the robustness of our conclusions.

We now provide the details for these new analyses in our Methods (Current Lines 540–549).

Original Lines 421–423: "*We hereafter refer to this subset of 1,353 sites as the ERITS annual time series (see Supplementary Table 1 for further site information).*"

Edited to:

Current Lines 548–557: "*We hereafter refer to this subset of 1,353 sites as the ERITS annual time series (see Supplementary Table 1 for further site information). Taxa in 56% of these sites (761 out of 1,353) were identified only to the family-level or higher, with 44% identified to a*

*mixed resolution, generally a combination of families, genera, and species, although the exact resolution can vary among countries. Such mixed-level identifications are common in invertebrate research where many taxa cannot be readily identified to the species or even genus level. Excluding sites with only family-level or higher identifications did not influence our results (Supplementary Figs. 7–8), indicating our findings were consistent whether we did or did not restrict our dataset to sites with only finer-scale taxonomic information.*"

**lines 428-429: although this is a sensible way to do it, this could be a problem and disregard extreme changes that could happen (such as a heat wave, for example). Again, this could be discussed as a limitation.**

We now highlight in the limitations section of our Discussion that gap filling can introduce error into some trend estimates.

Current Lines 321–325 (added): "*Additionally, if logistical or financial constraints preclude higher resolution monitoring, then complementary statistical approaches may help mitigate these issues, including gap filling[29] (as we used) or model-based methods to improve trend estimates from sparse data (e.g., hierarchical[68] or state-space[69] models). However, these approaches can also inflate error in some trend estimates[70].*"

**line 460-465: Maybe I missed this or misunderstood, but I don't see these results for the simulation.**

These models are used to produce the $R^2$ values presented in the frequency and duration simulations sections. We made some modifications to Fig. 1 to help readers understand how these values are produced, and we now direct readers to the relevant panel of that figure in this section to help make this link more clearly. Our addition of Fig. 5 (detailed in our response to your comment #4 above) also now more clearly outlines where these models fit in our analyses and results.

Original Lines 460–462: "*In addition to direction and magnitude matches, we related all site-level annual slope values to the simulated slope values to determine the degree to which the annual slopes could be predicted from the simulated slopes.*"

Edited to:

Current Lines 580–582: "*In addition to direction and magnitude matches, we related all the complete, site-level slopes to the simulated slopes to determine the degree to which variation in the annual slopes was reflected in the simulated slopes (i.e., the $R^2$ relationship shown in Fig. 1c and Fig. 5).*"

**This is just a subjective but the paragraphs 'Influence of reduced sampling frequency' and 'Influence of reduced sampling' are quite repetitive and could potentially be reorganized.**

We merged the two sections into a single section titled "*Influence of reduced sampling*". In this section, we first explain the general approach for linear models, simulations, and criteria for

comparisons, which applies to both frequency and duration, as you correctly point out. We then describe the details specific to frequency, duration, and both combined in separate paragraphs. For brevity, we provide here only the revised paragraph structure, which should now avoid repetition.

Current Lines 560–618:

"*Influence of reduced sampling*

*To evaluate how reducing sampling affected biodiversity trend estimation, we simulated time series with lower frequency, shorter duration, or a combination of both, and compared them to the complete time series. We calculated trends by fitting linear models of abundance, richness, coverage-based richness, or EQRs in relation to year. We initially tested for significant ($P < 0.05$) temporal autocorrelation between successive years within sites using the DHARMa package[78] but only found evidence for autocorrelation in 6.9–8.6% of sites (depending on the metric), so we did not control for this in our final models. All slopes were converted to percent change per year by log-transforming the metrics prior to modeling, then exponentiating the slopes and subtracting 1. This conversion ensured that slopes were comparable among sites regardless of differences in abundance units, taxonomic resolution, or total richness.*

*We first compared the complete versus simulated slopes using a vote-counting method whereby we compared the proportion of sites for which the simulated trends matched the direction or magnitude of the complete trends (Fig. 5). We considered the directions as matching if the complete versus simulated slopes had the same sign (i.e., positive or negative). Magnitudes matched if the simulated slope was within the standard error of the complete slope, which allowed for slight changes in direction to still match in magnitude. We could not compare slope significance because many frequency simulations provided only two years of data.*

*In addition to direction and magnitude matches, we related all the complete, site-level slopes to the simulated slopes to determine the degree to which variation in the annual slopes was reflected in the simulated slopes (i.e., the $R^2$ relationship shown in Fig. 1c and Fig. 5). We did so using generalized linear mixed models (GLMM) performed with the glmmTMB package[79]. All models included a random intercept term for 'country' to control for potential non-independence among slopes within the same countries, and we extracted the marginal $R^2$ of these relationships using the MuMIn package[80]. Furthermore, we compared differences between the complete versus simulated slope estimates by taking the absolute value of the difference for each site in each simulation and calculating the geometric mean of these differences across sites. We used the geometric mean to reduce the influence of a small number of sites with extreme trend estimates.*

*We simulated different sampling frequencies by only including data from every 2–6 years for each site (see Table 1 for a summary of analyses). Given we set the minimum time series duration to ten years, these frequencies meant that each simulation included at least two years of data to quantify trends. We chose these frequencies because they span a range commonly used in broad-scale monitoring programs (e.g., the EU Water Framework Directive recommends minimum sampling frequencies of every 3–6 years). For each site, we randomly selected whether sampling began in the first or second year of each time series and repeated this process 1,000 times.*

*We simulated shorter sampling durations by quantifying trends for the first 10 years in each annual time series, then re-quantifying these trends using only 2–9 years of the same time series. We focused on the first 10 years because this is the maximum comparable duration across*

*all time series, all of which spanned at least a decade. For each site, we randomly selected the starting year from all possible options within the first 10 years and repeated this 1,000 times.*

*Lastly, we examined the influence of combining reduced sampling frequency with reduced duration by repeating our frequency simulations but removing the last year of data in each, thus reducing duration as well. This approach restricted the simulations to frequencies of only every 2–4 years to ensure all time series provided at least two years of data.*
*For all above simulations, we compared slope direction, magnitude, $R^2$, and trend differences, ensuring that comparisons were always made using slopes quantified from the same starting years.*"

**line 496-497: 1km maximum of distance does not seem big, but out of curiosity (which could be of the reader as well), what does it mean for invertebrates? Is it big for invertebrate communities, or is there some reference that could back up his choice?**

Our edits shown in our response to your comment about line 147 should address this concern. The actual maximum distance was only ~115 m.

**line 516-517: Mentioning the interaction term, maybe you should conclude that you did not keep it in the final model? Was this also tested for the method part on the simulation data (or for consistency, either stated for both or not at all, maybe)?**

We revised this section to detail that we excluded the interaction from the final model. We also clarify that this test was only performed for the EEA vs. ERITS dataset comparison. It cannot be performed for the simulations because those comparisons are always performed within the same sites (i.e., a time series with more data compared to a time series from the same site with less data), so there is no role distance could play in these comparisons.

Original Lines 510–517: "*This model included the distance between site pairs as an interaction term to determine whether the relationship depended on site proximity, and a random intercept term for 'country'. From this model, we extracted the marginal $R^2$ to assess how well the slopes from the ERITS sites (i.e., the reference sites) predicted those from the EEA sites. We also tested the significance ($P < 0.05$) of the interaction with distance by dropping the interaction from the model and comparing the model with versus without the interaction using a likelihood ratio test. The interaction term was not significant ($P = 0.10$).*"

We split this text into a new paragraph to make it more obvious for readers and edited the wording:

Current Lines 645–653: "*Lastly, we tested the influence of distance on the modeled relationship between the EEA and ERITS trends, given the site pairs exhibited a range of distances between one another which could affect the relationship between their slopes. To test this, we included an interaction term for distance (m) between site pairs in the above GLMM. We then determined the significance ($P < 0.05$) of the interaction and the individual distance term by comparing models with and without these terms using Likelihood Ratio (LR) tests. The interaction was marginally non-significant (LR test, $L = 3.73$, $df = 1$, $P = 0.053$), and the individual distance term was also*

*non-significant (LR test, L = 1.01, df = 1, P = 0.31), indicating that controlling for the distance between site pairs would not substantially affect our results, so we excluded the distance term from the final model.*"

We thank the editor and reviewers for their help with improving our manuscript (NCOMMS-25-39065A) and for the opportunity to respond to their questions and concerns. We edited our text to provide the additional requested information, including added citations, clarification of our methods, improvements to our provided code/data, and further detail on our results. These changes have improved the quality and clarity of our manuscript and have not altered our principal results nor conclusions.

Point-by-point responses to the reviewer and editor concerns with the manuscript are listed below with line numbers. All data and R code needed to repeat our analyses are now publicly available from https://doi.org/10.6084/m9.figshare.28920428.

Thank you again for your continued assistance and consideration,

Sincerely and on behalf of our co-authors,

Daniela Cortés-Guzmán and James S. Sinclair

**Reviewer Comments and Responses**

**Manuscript ID:** NCOMMS-25-39065A

**MS title:** Robust estimates of biodiversity change require high-resolution time series

Bold comments are the reviewer comments with line numbers from the previous version of the manuscript (termed ‘Original’). Author comments are in non-bold text, with line numbers from the revised version of the manuscript (termed ‘Current’). Comments in italics are the relevant sections directly quoted from the manuscript text. We also highlighted all major edits in yellow both here and in the main text to aid with the review process.

**Reviewer #1 (Remarks to the Author):**

**I appreciated the additional context the authors now provide in the introduction regarding the theoretical background of the work. Thanks for examining the sensitivity of the time series lengths used and providing better linkage between the main article and the supplementary material. I also read the responses to other reviewers and appreciate the overall care the authors provided at this point in the review stage. I'm happy with the manuscript at this point and believe it is an important contribution to the field.**

We greatly appreciated the reviewer’s feedback and the time and assistance they provided for improving our work.

**<u>Reviewer's 2 authors comment review:</u>**

**This report details a review of the author's response to Reviewer 2 comments. Overall, most comments have been addressed but a few modifications and or additions are still needed. I am certain the authors can achieve what is proposed below. I also hope that my understanding of the reviewer 2 comments is in line with what he expected and that the authors will consider the proposed changes acceptable as well.**

***Major point 1: On the recognition of previous literature on long term data:***

**Overall, the authors added some interesting points which help to acknowledge existing studies. Maybe one additional aspect would be about the strategies to overcome the challenges of sparse time series. They use the following sentence: 'Various strategies have been developed to counteract these challenges, including statistical methods to address uncertainties and reduce biases arising from low resolution data (e.g., gap filling29).' In this sentence since they mention various strategies, it implies that they are multiple common practices, I encourage the authors to add one or two references from their manuscript instead of only showing one. This is to support the sentence point.**

We added a citation to Nakagawa (2015) to this section to better support the variety of data imputation methods that are available to ecologists.

***Major point 2: On the discussion of the results and the study conclusion:***

**The authors have provided good justification and added some valuable conclusions on the results comparison. One aspect I think could be beneficial for readers is what they described in their answer. I would have like to see (in the discussion for example within Current Lines 308–317) something along the lines of this part of their answer:**

**'We were initially hesitant to directly compare these results because the duration simulations were restricted to 10 years, whereas the frequency simulations can include longer time series (10, 15, 20+ years). This means the total amount of data per simulation is not always comparable. For example, a 10-year time series sampled every 2 years provides 5 years of data (comparable to the 5-year duration simulations), but a 20-year time series sampled every 2 years provides 10 years of data, which has no equivalent in the duration simulations. That said, you are correct that some generalizable differences remain.'.**

**The authors don't have to write such a long paragraph but something like: 'While duration simulations were restricted to 10 years, frequency simulations could span for longer periods (10, 15, 20 years, etc), making direct comparisons challenging due to unequal data sampled from these time series—though some generalizable differences still exist.'**

**The reasoning to add that is to acknowledge the differences coming from the simulation without taking out the main conclusions which I also think are still holding.**

We added some text to make this point in the suggested section.

Original Lines 310–314: "*We also found that trend estimates were less affected by declines in frequency than duration, as evidenced by higher site-by-site matches, higher explained variances, and lower differences compared to the annual trends. This result likely reflects the greater sensitivity of linear temporal trends to the initial and final years of a time series*[26,60]."

Edited to:

Current Lines 318–325: "*We also found that trend estimates were less affected by declines in frequency than duration, as evidenced by higher site-by-site matches, higher explained variances, and lower differences in the frequency compared to the duration simulations. We acknowledge that the duration and frequency results are not always directly comparable because the former was restricted to 10 years whereas the latter could encompass longer time periods, but some generalizable differences were still apparent. These differences likely reflect the greater sensitivity of linear temporal trends to the loss of either the initial or final years of a time series*[26,60]."

***Major point 3: On the ERITS estimates:***

**I think the authors added enough information to satisfy the point made by reviewer 2.**

We thank you for taking the extra time (and work!) to go over our responses to this reviewer.

***Major point 4: On comparing trends from two European datasets:***

**I believe the authors also provided good insights to explain the methods used there. I have a small note on the one paragraph they added:**

**Current Lines 645–653: "Lastly, we tested the influence of distance on the modeled relationship between the EEA and ERITS trends, given the site pairs exhibited a range of distances between one another which could affect the relationship between their slopes."**

**I think the sentence is a little bit confusing, I would suggest rewording it for example:**

**'Lastly, given that site pairs exhibited a range of distances between one another, we tested the influence of sites distance on the modeled relationship between the EEA and ERITS trends. Thus, we included..'**

We rephrased the sentence to improve clarity.

Original Lines 645–647: "*Lastly, we tested the influence of distance on the modeled relationship between the EEA and ERITS trends, given the site pairs exhibited a range of distances between one another which could affect the relationship between their slopes.*"

Edited to:

Current Lines 500–504: "*Lastly, because site pairs spanned a range of distances between one another that could influence the relationship between EEA and ERITS trend slopes, we included an interaction term for distance (m) between site pairs in the GLMM. We then determined the significance (P < 0.05) of this interaction and the individual distance term by comparing models with and without these terms using Likelihood Ratio (LR) tests.*"

**and concerning the following:**

**Current Lines 211–222: ' Additionally, while the average, across-site estimate of biodiversity change was similar between datasets (0.0030 ± 0.024% year-1 for ERITS versus -0.010 ± 0.19% year-1 for EEA), their signs differed, with the positive average trend in the ERITS dataset indicating slight temporal improvement in ecological quality, whereas the negative average trend in the EEA dataset indicated slight degradation."'.**

**Instead of their signs differed, I would take some precautions to say 'could differ' since the value is -0.010 ± 0.19.**
**and accordingly:**

**Current Lines 257–268: "The lower temporal resolution of the EEA sites also resulted in an opposite estimate of the direction of overall change compared to the ERITS dataset..' should be changed to: The lower temporal resolution of the EEA sites could also result in an opposite estimate of the direction of overall change compared to the ERITS dataset'**

We hedged the sentence in the results, as suggested. We decided to remove the sentence in the discussion, which improves the flow of the paragraph. With a small modification, the key message is better convened in the next sentence.

Original Lines 218–220: "*Additionally, while the average, across-site estimate of biodiversity change was similar between datasets (0.0030 ± 0.024% year$^{-1}$ for ERITS versus -0.010 ± 0.19% year$^{-1}$ for EEA), their signs differed…*"

Edited to:

Current Lines 226–228: "*Additionally, while the average, across-site estimate of biodiversity change was similar between datasets (0.0030 ± 0.024% year$^{-1}$ for ERITS versus -0.010 ± 0.19% year$^{-1}$ for EEA), their signs may differ…*"

Original Lines 257–258: "*The lower temporal resolution of the EEA sites also resulted in an opposite estimate of the direction of overall change compared to the ERITS dataset, although both estimate values were similar and close to zero, likely due to high variability, particularly in the EEA trends. While differences in the overall outcomes were minimal, their contrasting directions and the generally differing perspectives of change…*"

Edited to:

Current Lines 269–270: "*While differences in the overall outcomes were minimal, their potentially contrasting directions and the generally differing perspectives of change…*"

***Regarding data and code reproducibility:***

**I think I had made some comments on the code within my own second-round review already.**

**A few other comments:**

**Writing tone and big statement: I believe the authors did modify the text according to the reviewer's comment and they also gave more specific conclusions as suggested by other reviewers.**

**Interpolation of missing values with the mean of the neighboring values: I think the changes were fair, but I suggest some additional small changes:**

**Current Lines 93–96: "We quantified trends in the above metrics for 1,353 European river sites using annual data derived from nearly annual sampling with some gap filling (add here the reference to the corresponding method part).**

**Alternative approach to low data coverage: the sentence provided by the author is good but as mentioned above (point 1) it would be better with additional reference for other general strategies which could be coming from existing literature in the manuscript.**

We added the reference to the corresponding section in the Methods.

Original Lines 93–94: "*We quantified trends in the above metrics for 1,353 European river sites using annual data derived from nearly annual sampling with some gap filling.*"

Edited to:

Current Lines 94–96: "*We quantified trends in the above metrics for 1,353 European river sites using annual data derived from nearly annual sampling with some gap filling (see Annual time series in Methods).*"

**Literature on how to measure biodiversity: I think the authors only partially answered the reviewer's comment here. I suggest they carefully consider what the reviewer said here:**

**"Abundance as a measure is fraught and tightly linked to sampling effort. To a lesser but still substantial degree, so is richness. As a function of abundance and richness, EQR**

**metrics have the potential to amplify biases inherent to these measures (but this is hidden by the lack of transparency on what the metric formulations entail – it is not reasonable to expect readers to go hunting for 18 different formulations in the literature). The authors give a general statement that they do not think these types of biases matter, but the treatment is unconvincing."**

**I believe the authors should comment/add information on how these measures differ in their potential interference with biases. Since EQR is a sort of 'black box' measure which can be calculated in different ways, readers would benefit from a few sentences reflecting on its calculation and how each biodiversity measures might be impacted differently by the biases studied here.**

We have expanded the section of our Methods that discusses biases introduced by sampling effort to measures of abundance, richness, and ecological quality (Current Lines 407–415). We now acknowledge the linkages between these metrics, and provide a new column in Supplementary Table 2 that lists all metrics involved in the ecological quality calculations in each region. These changes make ecological quality less of a "black box", better acknowledge the biases in our chosen metrics, and better outline how we partly tested for the influence of these biases.

Original Lines 558–563: "*We represented biodiversity for each site and year based on four community metrics: (1) abundance (total number of individuals); (2) richness (total number of taxa); (3) coverage-based richness; and (4) ecological quality. Coverage-based richness accounts for potential biases in richness caused by differences in abundance, such as sites or periods with greater abundance requiring greater sampling effort to collect the same proportion of taxa as communities with lower abundance*[45]."

Edited to:

Current Lines 407–415: "*We represented biodiversity for each site and year based on three community metrics: (1) abundance (total number of individuals); (2) richness (total number of taxa); and (3) ecological quality. Temporal changes in these metrics are, however, potentially affected by the need to adjust sampling effort, such as sites or periods with greater abundance requiring greater sampling effort to collect the same proportion of taxa as communities with lower abundance*[78]*. Ecological quality can suffer from similar biases as it can include metrics of abundance and richness in its calculation (detailed in Supplementary Table 2). To partly test for these issues, we also quantified coverage-based richness for each site and year, which estimates taxon diversity if each sample was collected to the same degree of completeness.*"

**Figures:**

***Figure 2 and comment:***

**The authors mention that they did not comment on the sigmoidal and potential asymptote pattern in the text because this did not appear to be the case for other measures. I think**

**this still should be mentioned briefly as Reviewer 2 suggested. I proposed something along these lines in the results or discussion:**

**'Seldomly (or Occasionally), we noted some more complex patterns for longer time series which can be driven by poor matches (and poor R2) across all simulations once the amount of data has declined to a certain threshold (e.g., beginning around 2–4 years in the 10-year duration simulations, or 4–6 years in the 20-year simulations)'.**

**However, I am also now wondering why this happening so early in the time series? Could you elaborate on that and add an explanation or change the above sentence suggested?**

We specifically did not mention the asymptote in the upper left of the 20-year EQR results (Supplementary Fig. 4c), which does not appear in the other metrics nor in Fig. 2, but you are correct that we can highlight the asymptotes in the lower right, which is common to all metrics, including the results in Fig. 2. We added a few sentences to both our frequency and duration results for Fig. 2 in the main text to address your concern and to point this result out for readers (Current Lines 122–127 and 134–137). We do not re-iterate these same points again for the 20-year time series because we state that they exhibited "the same overall results" as Fig. 2, which now includes the discussion of the asymptotes.

Regarding why this happens so early in the time series, it is not that it is only happening early, but rather when the total duration has declined to a certain point. The duration trends can be derived from any possible starting year in the time series (e.g., a 3-year trend could be obtained starting early in year 1, 2, etc or later up to year 8). Our changes to explain the asymptotes for readers shown below should now be clearer that it is the total duration that matters.

Current Lines 122–127 (added): "*However, the increase in error rate for magnitude and $R^2$ exhibited an apparent asymptote whereby error no longer increased as severely once frequency declined to around once every 6 years (e.g., compare the higher degree to which matches declined from every 4 to every 5 years versus every 5 to every 6 years in Fig. 2a–c). This pattern suggests that, after sampling frequency has declined to a certain point, further data loss will not have as strong an influence on the error rate.*"

Current Lines 134–137 (added): "*We also found a similar asymptote for the duration error rate as we observed for frequency, with error rates reaching close to 0 around a total duration of 4 years and thus being unable to decline much further when duration declined to a total of 3 or 2 years.*"

***Figure 3:***

**I also agree with Reviewer 2 that scales comparability can be an issue here and this is not solved yet. Since some scales are matching I wonder if inverting the rows and columns would help to have at least comparable y axis across each row and thus improve the readability. I would also encourage the authors to try to match the x axis for a particular bias or another solution would be to add an overview figure where scale match (but details**

**are less visible) thus figure 3 would be a zoom in of that overview to see the actual values and ranges. I hope my explanation make sense.**

Thank you. Your suggestion substantially improved the readability of the figure. We have now reversed the axes of the panels in Figure 3. With consistent x-axes across panels within each column, the patterns should now be easier to compare despite variation in the y-axes. For consistency, the same modification was applied to Supplementary Figures 2, 5, and 8.

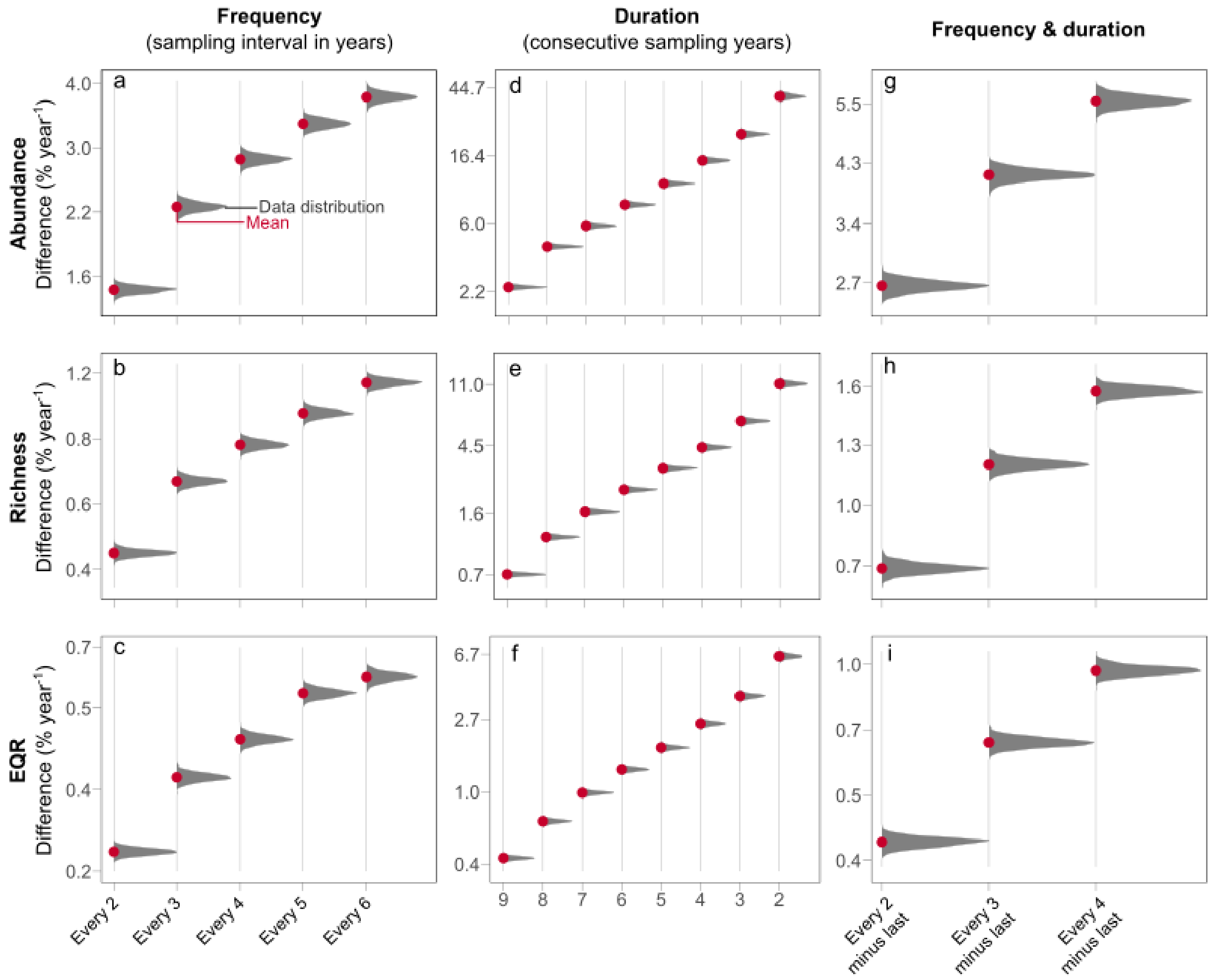


<u>New Figure 3 caption</u>: ***Influence of lower sampling frequency and duration on differences in biodiversity trends.*** *Lower (**a–c**) sampling frequency, (**d–f**) sampling duration, and (**g–i**) a combination of lower frequency and duration tended to increase the differences in trend values between the simulated monitoring schemes and those from the complete time series (i.e., annual or the first 10 years for duration alone). Trend differences are shown using density plots of the average, across-site absolute difference for each simulation (shaded polygons) and the average of these differences across simulations (red circles). Note that all y-axes are on the log-scale and that the range of this scale varies across panels.*

**<u>Reviewer #3 (Remarks to the Author):</u>**

**First of all, I would like to thank the authors for considering and answering my comments and questions. I think you have provided an extensive work to reflect and modify reviewers' comments in addition to extra analyses presented in the supplementary material. I also thank the author for providing their code, which of up most importance in our research work! I do have some comments and suggestions regarding this which I detail in the corresponding section.**

**I would below only highlight remaining comments I have on your work.**

**Remarks regarding the answers:**

**1) In one of your responses to my comments (point 3 of the general comments) regarding the time scaled displayed you said 'we opted to include the 10-year time series as it provided a more robust and broader-scale dataset'. I am not sure what you mean here, the 20 years would be more robust since you would have a longer time span or is it that the sampling during the 20 years is not enough spatially representative to be robust? i.e. less sites had longer time period. What I am going for here is that you could add a justification for the 10 years as being a trade-off between temporal and spatial dimensions if I understood your point correctly.**
**Note that I also read the response you made to Reviewer 1 which also is a good justification and information I don't necessarily have or other readers may not have as a none specialist.**

We apologize that our previous version was not clear enough, and your interpretation is correct. The subset of time series spanning at least 20 years includes substantially fewer sites and is therefore more spatially restricted than the full dataset. For this reason, we based our main analyses in the dataset of time series spanning a decade or more. We now clarify this point in the section describing the 20-year supplementary analysis:

<u>Original Lines 177–180</u>: "*An important caveat to the above results is that we restricted our dataset to time series spanning a decade or more (detailed in our Methods). However, even a decade can be insufficient for obtaining accurate trend estimates*[23,28,34,35]*. To test whether longer time series are more robust to data loss…*"

Edited to:

<u>Current Lines 187–188</u>: "*While our dataset provides broad spatial coverage, many sites are represented by time series of only ten years in length. To test whether longer time series are more robust to data loss…*"

**I do really appreciate the extensive discussion on the potential caveats!**

**Small suggestions:**

***Introduction:***

**Line 31-33: 'One common limitation is low temporal resolution. This limitation typically results from opportunistic data collection, or monitoring constraints of funding, time, and personnel'. -> These sentences seem to cut the flow of the paragraph due to the repetition. A subjective change (not compulsory) would be / or something similar: 'A common limitation is low temporal resolution which can raise from opportunistic data collection, or monitoring constraints of funding, time, and personnel.'**

We rephrased the sentence as suggested to improve flow and avoid repetition.

Original Lines 31–33: "*One common limitation is low temporal resolution. This limitation typically results from opportunistic data collection, or monitoring constraints of funding, time, and personnel.*"

Edited to:

Current 33–35: "*A common limitation is low temporal resolution, often arising from opportunistic data collection or constraints on monitoring resources, including funding, time, and personnel*."

**Line 34-35: I found this statement somehow misleading: 'such as just two or three sampling years per site7,8,10,14'. In particular, I think this does not reflect necessarily the truth from the papers cited there. If checking Dornelas paper, in the supplementary the table summarizing the time series data, most of them appear to have quite many years of sampling. Thus, I would suggest removing this highlighted part about 2 or 3 sampling years per site or to make it not as generalisable as what is currently written and using these references.**

This is a fair point. Our intention was not to suggest that the majority of time series in the cited studies are short, but rather to highlight that they can include some very short time series. We have revised the sentence to clarify our meaning.

Original Lines 33–35: "*Consequently, many broad-scale biodiversity studies quantify trends using data with a low sampling frequency or duration, such as just two or three sampling years per site*[7,8,10,14]."

Edited to:
Current 35–36: "*Consequently, many broad-scale biodiversity studies include time series with a low sampling frequency or duration, even sometimes just two or three sampling years per site*[7,8,10,14]."

*Results:*

**Paragraph starting line 177: This paragraph is a mix of results and discussion aspects. I suggest move certain sentences that are not descriptive to the discussion (For example lines 177-179 and lines 187-189.**

We removed the sections as suggested, both of which are already addressed in the Discussion.

**Figure 4, panel a: I was wondering when highlight in yellow direction matches and in red magnitude matches, how would be represented a match in both direction and magnitude? is it just separate points? I might be missing the point so please correct me or disregard if so. Otherwise the magnitude could be the point outside colour (although this would probably not be very easy to read as well.**
**Lastly the legend does not indicate what is the trend line. This is only element on that figure that is not explained in the legend so for consistency this would be a good addition.**

This is an insightful comment. In the previous version, matches in magnitude overlapped with matches in direction, preventing both from being visualized simultaneously. We therefore added a third color to Fig. 4a to represent those site pairs that match both in direction and magnitude, allowing all match types to be distinguished. We also added a description of the line representing the fitted regression predicted from the GLMM.

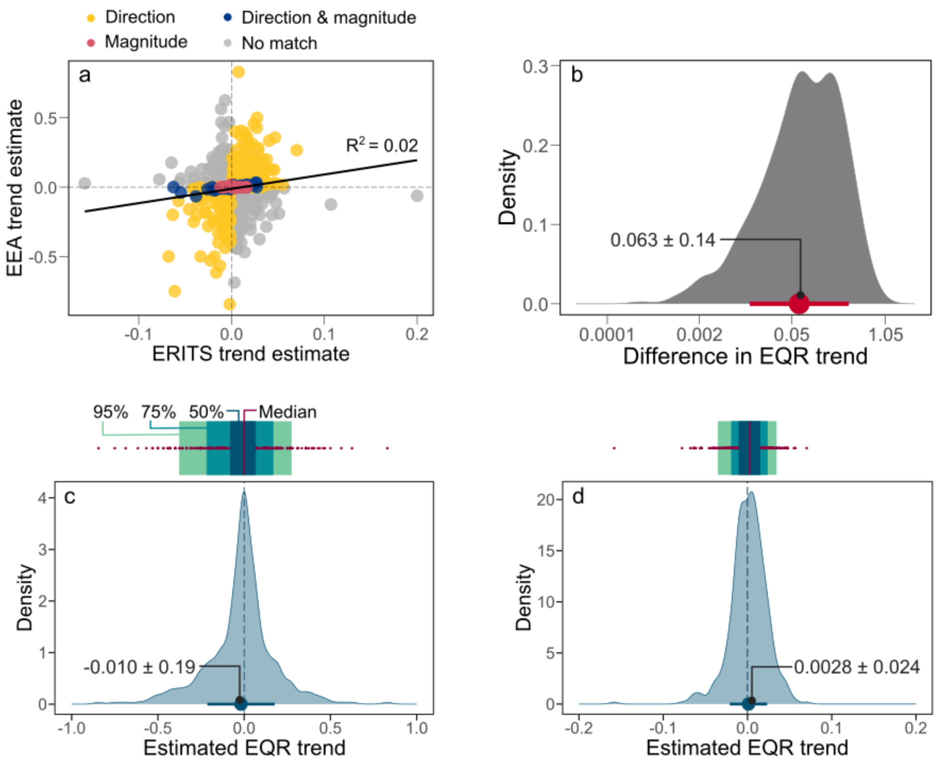


<u>New Figure 4 caption</u>: *Comparison of estimated EQR trends between two datasets that differ in temporal resolution. Differences in temporal resolution between the EEA and the ERITS sites influenced: (**a**) matches between the direction and magnitude of site-level ecological quality*

*trends (EQR change year$^{-1}$), with the fitted regression line and the corresponding $R^2$ from the GLMM; (**b**) trend estimates, represented as the absolute differences between the EEA and ERITS slope values; and (**c, d**) the average, across-site estimates of ecological quality change. Density plots in (**c, d**) show the probability distribution of trend estimates for the 485 sites, dots and error bars represent the mean and standard deviation, and the upper boxes show the median and distributions for 50%, 75%, and 95% of the data. Red dots show data outside 50%.*

***Discussion:***

**Line 287: Is the first time in the discussion that your study model appears river invertebrates (and also across Europe) even if you mention river sites in paragraph 3. I think a mention should come earlier in the discussion. Especially because already in paragraph 2 you comment on the existing result with numbers.**

We now specify the study system (European river invertebrate communities) in the second paragraph of the Discussion to provide context for the values presented there, while maintaining the generality of our findings.

Original Lines 236–237: "*We also found that even moderate limitations in temporal resolution led to incorrect trend estimates for many individual sites. For example, our least limited simulations…*"

Edited to:

Current Lines 246–248: "*We also found that even moderate limitations in temporal resolution led to incorrect trend estimates for many individual sites. In our dataset of European river invertebrate communities, for example, the least limited simulations…*"

***Code:***

**Overall, I think these codes are not user friendly and could benefits from a new organization (within the script), the actual csv files and a readme file to give the details, information that are on the excels file, version of R/Rstudio, packages and their version used) etc..**

We made the requested changes to improve the user-friendliness of the README, file structure, and code, which we detail below in response to each of your more specific comments.

***Simulation code:***

**The first csv file does not exist since the site file you provided is a xlsx document (multiple sheets). I understand that it is easy for everyone to correct this but for simplicity of running the analyses, it would be beneficial for the reader.**

We now introduce and describe each Excel file in the README, which are all now in the same, higher folder as the README and zipped code folders. We also now provide the individual CSVs needed for each R code to run within each of the zipped code folders so users no longer have to save the data tabs from the Excel files as separate CSVs themselves.

**I have to say that a script of 3700+ lines is very long to check and to follow. Please consider dividing per topics and providing smaller simulation sets as an example.**

The simulation code is just the repetition of the same general method for each metric and frequency/duration simulation. Thus, to reduce the code's length, we now provide only the code needed to run a single metric and a single frequency and duration simulation, and detail for readers how these examples can be easily repeated for the other metrics and simulations. This has reduced the code to about 10% of its original length, from 3700+ lines to just 350+ lines.

**I run into an error line 2462, running this r.squaredGLMM(mod)**
**Error in model.matrix(object)[, ok, drop = FALSE] :**
**(subscript) logical subscript too long**
**It might be working for you or be a version incompatibility of packages or else but please revise if needed the scripts and precise the version of R and packages that you are using.**

We triple checked this function on multiple machines only using the provided code and files and no errors occurred. We now include the version of R we are using in the provided code, along with the versions of each package.

**I ran into another error later on but could no longer keep track since my computer crashed when running this big script (even if I run it for a smaller sample of sites). Again, knowing if this analysis was run all together or not, making separate files and or indicating if remote desktop or super computer or else was used would be useful.**

The substantially reduced length of the simulation code should now prevent these issues from occurring. We've also confirmed that, after loading the two data files as instructed by the annotations, the code can be run all together on any machine and we now indicate this for users in the annotations.

***ERITS vs EEA code:***

**Same comment regarding the file extension**

We now provide the required CSV file in the folder alongside this code.

Reviewer comments for “Robust estimates of biodiversity change require high-resolution time series”

**Summary**
Cortés-Guzmán, Sinclair, and others use a large riverine time series biodiversity database to understand the impact of temporal data resolution on biodiversity trends. The authors found that less temporal resolution (both duration and frequency of sampling) often led to incorrect conclusions on trends, but could often still be used to detect changes in biodiversity direction. Higher resolution time series were needed to detection changes in both direction and magnitude. The authors also include a nice comparison of two time series and provide specific ideas for how sampling may affect trend assessments and management. I have a few suggested comments below, but in general, I think this is an excellent paper.

**Major comments**

- I like the figure 1 conceptual explanation of the work
- I appreciated the open data and open code associated with the project
- I think the authors do a good job of setting up the implications of the work and applied examples related to time series bias in the introduction. However, I believe a more detailed treatment of the theoretical work on the topic (mostly around paragraphs 2+3) would be helpful. As the reviewers note, there is established literature on the topic, but it is broader and includes both theory and work with other biodivevrsity databases. Here are just a few examples, but I am biased towards some of the work more closely connected to my own. I’m not suggested the authors need to add all, or any, of these references, but I think discussing past work in the introduction, or discussion, more connected to the theoretical treatment of the topic might be helpful.
    - Chades, I., T. G. Martin, S. Nicol, M. A. Burgman, H. P. Possingham, and Y. M. Buckley. 2011. “General Rules for Managing and Surveying Networks of Pests, Diseases, and Endangered Species.” *Proceedings of the National Academy of Sciences* 108 (20): 8323–28. https://doi.org/10.1073/pnas.1016846108.
    - White, Easton R. 2019. “Minimum Time Required to Detect Population Trends: The Need for Long-Term Monitoring Programs.” *BioScience* 69 (1): 40–46. https://doi.org/10.1093/biosci/biy144.
    - Bennett, Joseph R., Sean L. Maxwell, Amanda E. Martin, Iadine Chadès, Lenore Fahrig, and Benjamin Gilbert. 2018. “When to Monitor and When to Act: Value of Information Theory for Multiple Management Units and Limited Budgets.” *Journal of Applied Ecology* 55 (5): 2102–13. https://doi.org/10.1111/1365-2664.13132.
    - Bennett, Joseph R., Kathleen M. Rühland, and John P. Smol. 2016. “No Magic Number: Determining Cost-Effective Sample Size and Enumeration Effort for Diatom-Based Environmental Assessment Analyses.” *Canadian Journal of Fisheries and Aquatic Sciences* 74 (2): 208–15. https://doi.org/10.1139/cjfas-2016-0066.
    - Bennett, Joseph R., Danielle R. Sisson, John P. Smol, Brian F. Cumming, Hugh P. Possingham, and Yvonne M. Buckley. 2014. “Optimizing Taxonomic

Resolution and Sampling Effort to Design Cost-Effective Ecological Models for Environmental Assessment." *Journal of Applied Ecology* 51 (6): 1722–32. https://doi.org/10.1111/1365-2664.12312.
    - Brashares, Justin S, and Moses K Sam. 2005. "How Much Is Enough? Estimating the Minimum Sampling Required for Effective Monitoring of African Reserves." *Biodiversity and Conservation* 14: 2709–22. https://doi.org/10.1007/s10531-005-8404-z.
    - Field, Scott A, Patrick J O Connor, Andrew J Tyre, and Hugh P Possingham. 2007. "Making Monitoring Meaningful." *Austral Ecology* 32: 485–91. https://doi.org/10.1111/j.1442-9993.2007.01715.x.
    - Legg, Colin J, and Laszlo Nagy. 2006. "Why Most Conservation Monitoring Is, but Need Not Be, a Waste of Time." *Journal of Environmental Management* 78: 194–99. https://doi.org/10.1016/j.jenvman.2005.04.016.
    - Seavy, Nathaniel E., and Michelle H. Reynolds. 2007. "Is Statistical Power to Detect Trends a Good Assessment of Population Monitoring?" *Biological Conservation* 140 (1–2): 187–91. https://doi.org/10.1016/j.biocon.2007.08.007.
    - Tulloch, Ayesha I. T., Iadine Chadès, and Hugh P. Possingham. 2013. "Accounting for Complementarity to Maximize Monitoring Power for Species Management: Complementary Monitoring Indicators." *Conservation Biology* 27 (5): 988–99. https://doi.org/10.1111/cobi.12092.
- Why use the shorter time series of ~10 years in the study? I think it is hard to get a sense if the trend from the 10 year time series is a fair "true trend" comparison
    - Relatedly, I'm not sure how the results starting on line 88 can make sense if there was sampling once every 6 years if there was a 10-year time series
- Early on (perhaps in the last paragraph of the introduction), I think it is important to clarify what you mean by "trend analysis" or "trends". A lot of folks use this to mean simple linear trends, which I believe is what was done here, but that doesn't have to be the case. I would just clarify that is what you are doing. In the discussion, I would then note as a limitation that you only looked at linear trends over time and how that could be the avenue of future work. There is already theoretical literature on linear vs nonlinear time series ideas related to biodiversity.
- For future work (it feels like too much for this paper), it may be interesting to try and understand the why of time series robustness. For example, the authors could see if trends were more robust to temporal resolution dependent on the taxa, location, etc.
- Line 202: I'd be careful about noting what your paper says versus previous literature as the current work doesn't say anything about specific management actions. I think some simple rephrasing here might be helpful
- Line 224-230: Referring to the literature I referenced above, I think this paragraph could be better grounded in the literature
- Line 241: You note the work provides a "rule-of-thumb", but I'm not sure what you are getting at. I think you might be trying to note that low resolution datasets can provide information on direction while higher resolution datasets are needed for other questions, but I'm not sure.
- In the discussion, I would come back to the limitation of this being a particular set of taxa in a particular set of systems.

**Minor comments**

- Figure 2: Perhaps use different shapes for the "Type of match". They were hard to distinguish in black and white
- Figure 2: I found the y-axis initially confusing. Perhaps it would be better to have "Degree of match" closer to the axis and the words Abundance, Richness, EQR to be bold and further to the left (more akin to figure 3)
- Figure 2: Why use size to represent SD instead of simple error bars?
- Line 261: I like this very clear example of the implications of the sampling frequency findings.

**Reviewer:** Easton White

Reviewer's 2 authors comment review:

This report details a review of the author's response to Reviewer 2 comments. Overall, most comments have been addressed but a few modifications and or additions are still needed. I am certain the authors can achieve what is proposed below. I also hope that my understanding of the reviewer 2 comments is in line with what he expected and that the authors will consider the proposed changes acceptable as well.

**Major point 1:** On the recognition of previous literature on long term data:

Overall, the authors added some interesting points which help to acknowledge existing studies. Maybe one additional aspect would be about the strategies to overcome the challenges of sparse time series. They use the following sentence:

*'Various strategies have been developed to counteract these challenges, including statistical methods to address uncertainties and reduce biases arising from low resolution data (e.g., gap filling29). '*

In this sentence since they mention various strategies, it implies that they are multiple common practices, I encourage the authors to add one or two references from their manuscript instead of only showing one. This is to support the sentence point.

**Major point 2:** On the discussion of the results and the study conclusion:

The authors have provided good justification and added some valuable conclusions on the results comparison. One aspect I think could be beneficial for readers is what they described in their answer. I would have like to see (in the discussion for example within Current Lines 308–317) something along the lines of this part of their answer: '*We were initially hesitant to directly compare these results because the duration simulations were restricted to 10 years, whereas the frequency simulations can include longer time series (10, 15, 20+ years). This means the total amount of data per simulation is not always comparable. For example, a 10-year time series sampled every 2 years provides 5 years of data (comparable to the 5-year duration simulations), but a 20-year time series sampled every 2 years provides 10 years of data, which has no equivalent in the duration simulations. That said, you are correct that some generalizable differences remain.'*

The authors don't have to write such a long paragraph but something like: 'While duration simulations were restricted to 10 years, frequency simulations could span for longer periods (10, 15, 20 years, etc), making direct comparisons challenging due to unequal data sampled from these time series—though some generalizable differences still exist.'

The reasoning to add that is to acknowledge the differences coming from the simulation without taking out the main conclusions which I also think are still holding.

**Major point 3:** On the ERITS estimates:

I think the authors added enough information to satisfy the point made by reviewer 2.

**Major point 4:** On comparing trends from two European datasets:

I believe the authors also provided good insights to explain the methods used there. I have a small note on the one paragraph they added:

Current Lines 645–653: "*Lastly, we tested the influence of distance on the modeled relationship between the EEA and ERITS trends, given the site pairs exhibited a range of distances between one another which could affect the relationship between their slopes.*"

I think the sentence is a little bit confusing, I would suggest rewording it for example:

'*Lastly, given that site pairs exhibited a range of distances between one another, we tested the influence of sites distance on the modeled relationship between the EEA and ERITS trends. Thus, we included..'*

and concerning the following:

Current Lines 211–222: ' *Additionally, while the average, across-site estimate of biodiversity change was similar between datasets (0.0030 ± 0.024% year-1 for ERITS versus -0.010 ± 0.19% year-1 for EEA), their signs differed, with the positive average trend in the ERITS dataset indicating slight temporal improvement in ecological quality, whereas the negative average trend in the EEA dataset indicated slight degradation.*"'

Instead of their signs differed, I would take some precautions to say 'could differ' since the value is -0.010 ± 0.19.

and accordingly:

Current Lines 257–268: "*The lower temporal resolution of the EEA sites also resulted in an opposite estimate of the direction of overall change compared to the ERITS dataset..'*

should be changed to:

The lower temporal resolution of the EEA sites could also result in an opposite estimate of the direction of overall change compared to the ERITS dataset'

**Regarding data and code reproducibility:**

I think I had made some comments on the code within my own second-round review already.

**A few other comments:**

- **Writing tone and big statement**: I believe the authors did modify the text according to the reviewer's comment and they also gave more specific conclusions as suggested by other reviewers.

- **Interpolation of missing values with the mean of the neighboring values:** I think the changes were fair, but I suggest some additional small changes:

Current Lines 93–96: "*We quantified trends in the above metrics for 1,353 European river sites using annual data derived from nearly annual sampling with some gap filling (*add here the reference to the corresponding method part*).*

- **Alternative approach to low data coverage**: the sentence provided by the author is good but as mentioned above (point 1) it would be better with additional reference for other general strategies which could be coming from existing literature in the manuscript.

- Literature on how to measure biodiversity: I think the authors only partially answered the reviewer's comment here. I suggest they carefully consider what the reviewer said here: **Abundance as a measure is fraught and tightly linked to sampling effort. To a lesser but still substantial degree, so is richness. As a function of abundance and richness, EQR metrics have the potential to amplify biases inherent to these measures (but this is hidden by the lack of transparency on what the metric formulations entail – it is not reasonable to expect readers to go hunting for 18 different formulations in the literature). The authors give a general statement that they do not think these types of biases matter, but the treatment is unconvincing.** I believe the authors should comment/add information on how these measures differ in their potential interference with biases. Since EQR is a sort of 'black box' measure which can be calculated in different ways, readers would benefit from a few sentences reflecting on its calculation and how each biodiversity measures might be impacted differently by the biases studied here.

- **- Lines 29 – 36** corrections are ok and one part of the changed/added paragraph is already mentioned in my previews comments.

**Figures**:

Figure 2 and comment:

The authors mention that they did not comment on the sigmoidal and potential asymptote pattern in the text because this did not appear to be the case for other measures. I think this still should be mentioned briefly as Reviewer 2 suggested. I proposed something along these lines in the results or discussion:

'Seldomly (or Occasionally), we noted some more complex patterns for longer time series which can be driven by poor matches (and poor R2) across all simulations once the amount of data has declined to a certain threshold (e.g., beginning around 2–4 years in the 10-year duration simulations, or 4–6 years in the 20-year simulations)'.

However, I am also now wondering why this happening so early in the time series? Could you elaborate on that and add an explanation or change the above sentence suggested?

Figure 3: I also agree with Reviewer 2 that scales comparability can be an issue here and this is not solved yet. Since some scales are matching I wonder if inverting the rows and columns would help to have at least comparable y axis across each row and thus improve the readability. I would also encourage the authors to try to match the x axis for a particular bias or another solution would be to add an overview figure where scale match (but details are less visible) thus figure 3 would be a zoom in of that overview to see the actual values and ranges. I hope my explanation make sense.
